# Polymers and Polymer-Based Materials for the Detection of (Nitro-)explosives

**DOI:** 10.3390/ma16186333

**Published:** 2023-09-21

**Authors:** Olga S. Taniya, Albert F. Khasanov, Leila K. Sadieva, Sougata Santra, Igor L. Nikonov, Wahab K. A. Al-Ithawi, Igor S. Kovalev, Dmitry S. Kopchuk, Grigory V. Zyryanov, Brindaban C. Ranu

**Affiliations:** 1 Chemical Engineering Institute, Ural Federal University, 19 Mira Str., 620002 Yekaterinburg, Russia; olga.tania@urfu.ru (O.S.T.); a.f.khasanov@ya.ru (A.F.K.); l.k.sadieva@urfu.ru (L.K.S.); sougatasantra85@gmail.com (S.S.); rodonid93@mail.ru (I.L.N.); valitkhavi@urfu.ru (W.K.A.A.-I.); ekls85@yandex.ru (I.S.K.); dkopchuk@mail.ru (D.S.K.); bcranu@gmail.com (B.C.R.); 2 I. Ya. Postovsky Institute of Organic Synthesis of RAS (Ural Division), 22/20 S. Kovalevskoy/Akademicheskaya Str., 620219 Yekaterinburg, Russia; 3 Energy and Renewable Energies Technology Center, University of Technology-Iraq, Baghdad 10066, Iraq; 4 School of Chemical Sciences, Indian Association for the Cultivation of Science, Jadavpur, Kolkata 700032, India

**Keywords:** polymers, nitro-explosives, materials, detection methods, fluorescent materials

## Abstract

Methods for the remote detection of warfare agents and explosives have been in high demand in recent times. Among the several detection methods, fluorescence methods appear to be more convenient due to their low cost, simple operation, fast response time, and naked-eye-visible sensory response. For fluorescence methods, a large variety of fluorescent materials, such as small-molecule-based fluorophores, aggregation-induced emission fluorophores/materials, and supramolecular systems, have been reported in the literature. Among them, fluorescent (bio)polymers/(bio)polymer-based materials have gained wide attention due to their excellent mechanical properties and sensory performance, their ability to recognize explosives via different sensing mechanisms and their combinations, and, finally, the so-called amplification of the sensory response. This review provides the most up-to-date data on the utilization of polymers and polymer-based materials for the detection of nitroaromatic compounds (NACs)/nitro-explosives (NEs) in the last decade. The literature data have been arranged depending on the polymer type and/or sensory mechanism.

## 1. Introduction

In the last several decades, terroristic threats have become an actual problem worldwide, and these threats are mainly associated with the use of warfare agents or explosive devices against civilian transport and/or objects of civilian infrastructure. Therefore, approaches to the remote detection of warfare agents and explosives are in high demand. Concerning the latter, the use of detection dogs is widely considered the most effective and adaptive tool for explosive detection [1]. However, there are several disadvantages to using dogs for that purpose. First of all, dogs are unable to work by themselves, only functioning in tandem with a handler, who is fully responsible for correctly judging the dog’s behavior during the explosives search. Second, dogs cannot be worked as intensely as a piece of machinery (not more than half an hour). Third, dogs are also vulnerable to distraction by external factors, such as new surroundings, bright lights and loud noises, fatigue, and even alluring scents left behind by canine members of the opposite sex. Therefore, studies on using other mammals/rodents, such as pigs [2] or rats [3], are in progress. And thus, the olfactory systems of rats and pigs are considered promising objects for studies on explosive detection [4]. Some other studies are associated with investigating insects/insect-based biodevices for explosive detection [5,6,7,8,9]. 

Other approaches to explosive detection include using physical methods, such as mass spectrometry [10], differential mobility spectrometry [11], electrochemical methods [12], microcantilever devices [13], neutron techniques [14], nuclear quadrupole resonance methods [15], X-ray diffraction methods [16], millimeter-wave [17] or terahertz imaging [18], and, finally, laser-based detection methods [19]. The main disadvantages of these methods are the lack of portability, a need for special algorithms for transferring the sensory event to a readable signal, a noticeable time delay from the sensing event to the physical signal, the high cost of equipment, and, in most cases, the requirement for operation by highly trained personnel. In this regard, the use of colorimetric [20,21] or fluorescence methods [22] seems to be more convenient due to their low cost, simpler operation, fast response time, and naked-eye-visible sensory response. For fluorescence methods, a large variety of fluorescent materials, such as small-molecule-based fluorophores, aggregation-induced emission fluorophores/materials, and supramolecular systems, have already been reported in the literature [22]. Among them, fluorescent (bio)polymers/(bio)polymer-based materials [23,24,25] have gained wide attention due to their excellent mechanical properties and sensory performance, their ability to recognize explosives via different sensing mechanisms and their combinations, and, finally, the so-called amplification of the sensory response. 

For the evaluation and comparison of the quenching efficiency of sensors and probes, the Stern–Volmer mathematical model is usually used. If the static quenching mechanism takes place, the quenching constant (Stern–Volmer constant), *K_SV_*, should increase linearly with the quencher concentration, as described by the Stern–Volmer equation (Equation (1)):

(1) $$\frac{I_{0}}{I}=1+K_{SV}\cdot [Q]$$

where *I*_0_ and *I* are the fluorescence intensity before and after the addition of a nitroaromatic compound (quencher); [*Q*] is the concentration of the quencher, mol/L; and *K_SV_* is the value of the quenching constant, M^−1^.

According to Equation (1), the plot of *I*_0_/*I* vs. [*Q*], or the so-called Stern–Volmer plot, should give a straight line with a slope equal to *K_SV_*.

During the static quenching mechanism, the lifetime of the luminescence does not depend on the concentration of the quencher and remains unchanged as the quencher concentration increases. 

Dynamic quenching is usually associated with collisions between molecules of a sensor and an analyte, and the Stern–Volmer equation is represented as Equation (2):

(2) $$\frac{I_{0}}{I}=1+k_{q}\tau_{0}\times \left[Q\right]$$

where *k_q_* and *τ*_0_ are the bimolecular quenching constant and the lifetime of the fluorophore in the absence of a quencher, respectively.

This review provides the most up-to-date data on the utilization of polymers and polymer-based materials for the detection of nitroaromatic compounds (NACs)/nitro-explosives (NEs) in the last decade. The literature data are arranged depending on the polymer type and/or sensory mechanism.

## 2. Common Approaches to the Detection of NACs by Using Polymer-Based Materials

Conjugated polymers (**CP**s) are considered attractive materials for the fluorescence detection of various (bio)analytes since, in most cases, **CP**s have high fluorescence quantum yields, large extinction coefficients, and efficient optical signal transduction, and the synthetic versatility of **CP**s allows the incorporation of a wide selection of functional groups into the polymer backbone or into the side arms for better performance. Below, several common approaches to using polymers for explosive detection are highlighted.

### 2.1. Linear Conjugated Polymers

**Figure 1:**
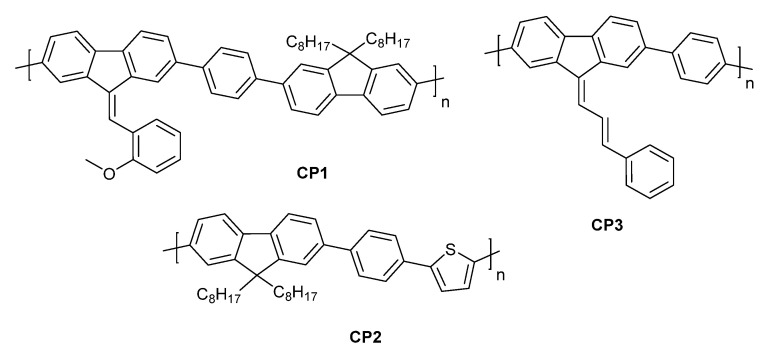
Structures of **CP1**, **CP2**, and **CP3**.

**Figure 2:**
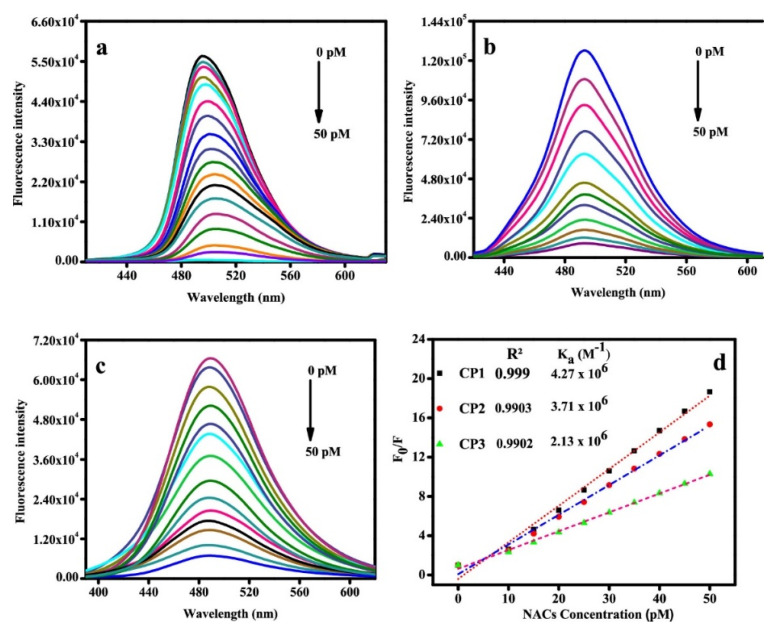
Spectral change in fluorescence emission behavior of the polymers (**a**) **CP1**, (**b**) **CP2**, and (**c**) **CP3** (10 μM) in aqueous solution (H_2_O/THF (3:2, *v*/*v*)) and (**d**) changes in the SV plot of polymers **CP1**, **CP2**, and **CP3** treated with different concentrations of TNP explosive (0–50 pM). Reproduced with permission from reference [26]. Copyright © ACS Publications 2022.

**Figure 3:**
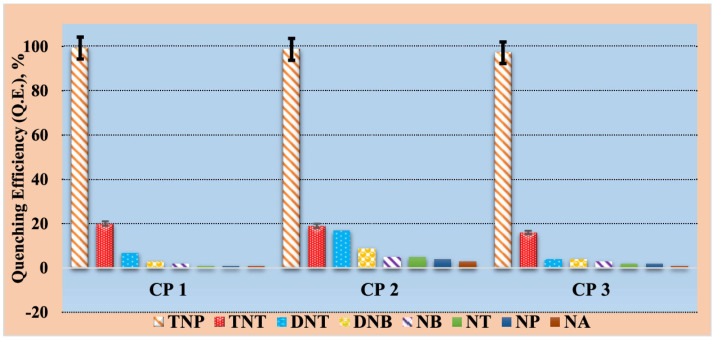
Graphical representation of quenching efficiency of polymers **CP1**, **CP2**, and **CP3** (10 μM) treated with different NACs, including TNP, TNT, DNT, DNB, NB, NT, NP, and NA. Reproduced with permission from reference [26]. Copyright © ACS Publications 2022.

**Figure 4:**
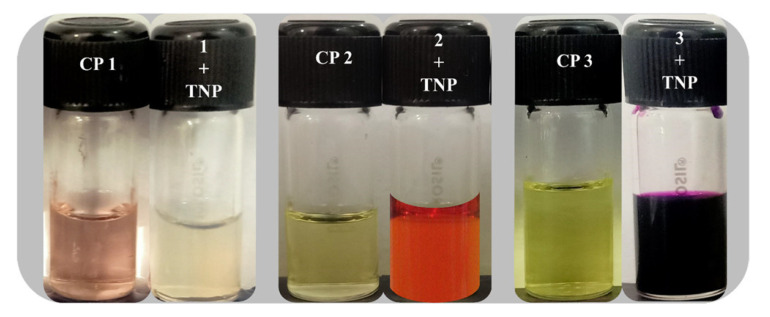
Polymers **CP1**, **CP2**, and **CP3** (10 μM) changing to different colors with the addition of 500 nM PA in daylight. Reproduced with permission from reference [26]. Copyright © ACS Publications 2022.

Batool and coworkers designed fluorene-based fluorometric and colorimetric conjugated polymers (**CP**s) (Figure 1) for the sensitive detection of PA in an aqueous medium at the picomolar (pM) level [26]. These polymers possess a high conjugation degree and electron-donating ability due to the presence of fluorene units. Therefore, these **CP**s demonstrated great sensitivity and selectivity toward picric acid (PA) over other NACs, with Stern–Volmer quenching constant, K_SV,_ values of 4.27 × 10^6^ M^−1^, 3.71 × 10^6^ M^−1^, and 2.13 × 10^6^ M^−1^ in an aqueous solution (H_2_O/THF (3:2, *v*/*v*)) for **CP1**, **CP2**, and **CP3**, respectively (Figure 2 and Figure 3). The limits of detection (LODs) for PA were calculated to be 3.2, 5.7, and 6.1 pM, respectively. For all of the **CP**s, the static quenching mechanism could be inferred from the high linearity of the Stern–Volmer plots (Figure 2d), bathochromic shifts in UV/Vis absorption spectra upon the addition of PA, and the insignificant overlap between the absorption of PA and the fluorescence emission of the **CP**s. The authors confirmed the PET mechanism by analyzing the HOMO–LUMO energy levels of **CP**s and PA. In this case, **CP1**, **CP2**, and **CP3** were characterized by higher-lying LUMO levels (−1.63, 1.73, and −1.84 eV, respectively) compared to PA (−4.56 eV), with the smallest ΔE_LUMO_ calculated for **CP1**, which agrees with the experimental data. In addition, **CP**s exhibited a naked-eye-visible response after the addition of 500 nM PA to H_2_O/THF (3:2, *v*/*v*) solutions (Figure 4).

**Figure 5:**
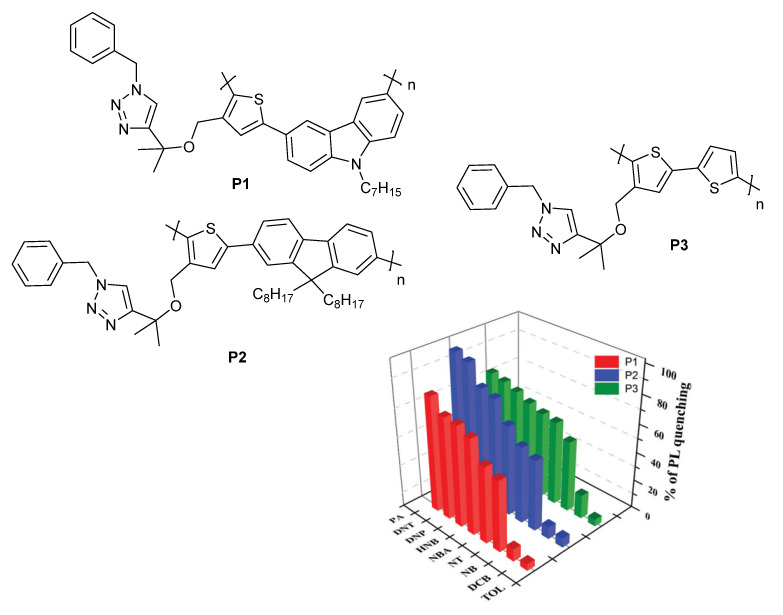
Emission-quenching percentage for polymers **P1**–**P3** toward different NACs and non-nitroaromatic compounds in chloroform. Reproduced with permission from reference [27]. Copyright © Royal Society of Chemistry 2020.

**Figure 6:**
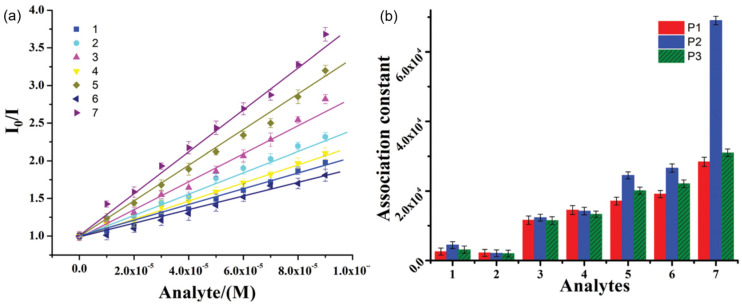
(**a**) Results in Stern–Volmer plot for polymer **P2**. (**b**) Graphical representation of the association constant comparison of polymers **P1–P3** for different nitroaromatics (1: NB; 2: NT; 3: NBA; 4: HNB; 5: DNP; 6: DNT; 7: PA). Reproduced with permission from reference [27]. Copyright © Royal Society of Chemistry 2020.

**Figure 7:**
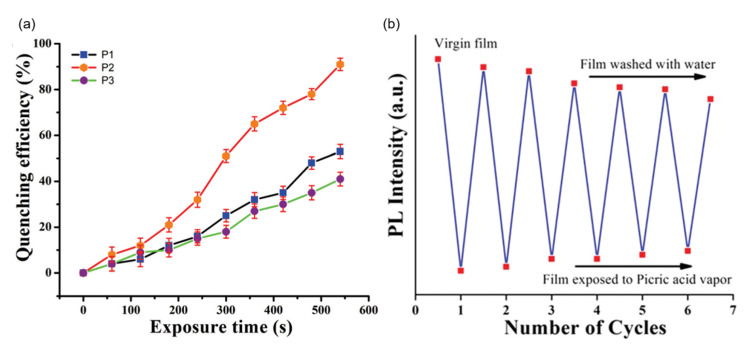
(**a**) Graphical representation of the superiority of polymer **P2** over polymers **P1** and **P3**, as indicated by the relative quenching efficiency. (**b**) Emission-quenching cycles and demonstration of reusability through recovery after exposing the probe film to PA vapor for 200 s. Reproduced with permission from reference [27]. Copyright © Royal Society of Chemistry 2020.

Giri and Patra reported 1,2,3-triazolyl-functionalized carbazole- (**P1**), fluorene- (**P2**), and thiophene-based (**P3**) A-alt-B-type π-conjugated copolymers for the selective and sensitive detection of NACs in aqueous media and the vapor phase [27]. Due to the high conjugation degree based on typical donating units, these **P1**–**P3** copolymers demonstrated highly intense fluorescence with quantum yields in the range of 18–20%. The **P1**–**P3** polymers exhibited a fluorescence-quenching response to NACs with high quenching efficiency (Figure 5). PA was found to be the most efficient quencher for all of the polymers, with the highest K_SV_ = 6.4 × 10^4^ M^−1^ for **P2** (Figure 6b). Based on the high linearity of the Stern–Volmer plot (Figure 6a) and the higher-lying LUMO levels of **P1**–**P3** compared to those of the NACs, the authors declared that static quenching occurred by the PET mechanism. **P1**–**P3**-based thin films demonstrated luminescence quenching of 91%, 53%, and 48%, respectively, after exposure for 200 s (Figure 7a). In addition, the authors demonstrated the reusability of the films based on the number of cycles of exposure and washing with water (Figure 7b), showing the applicability of the prepared probes.

**Figure 8:**
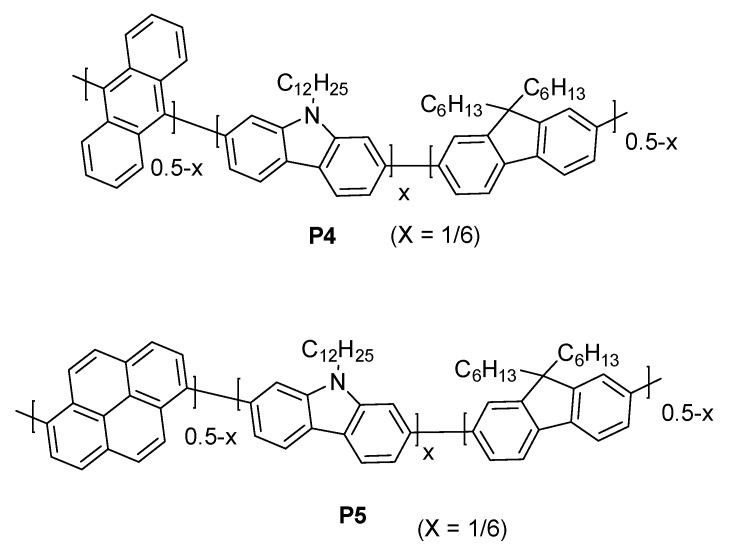
Structures of polymers **P4** and **P5**.

**Figure 9:**
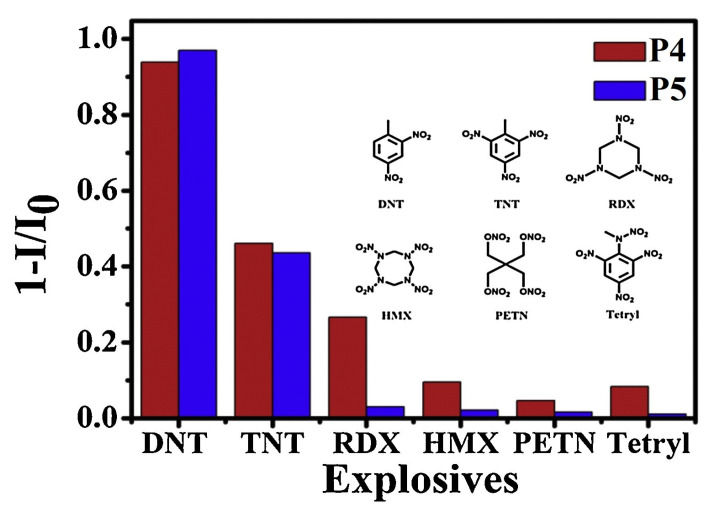
Graphical representation of different fluorescence-quenching ratios of polymers **P4** and **P5** exposed to nitro-explosives DNT, TNT, RDX, HMX, PETN, and Tetryl vapor for 5 s. Reproduced with permission from reference [28]. Copyright © Elsevier 2019.

**Figure 10:**
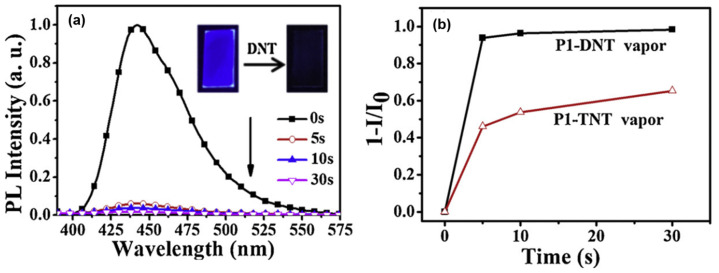
(**a**) Graphical representation of fluorescence intensity of **P1** films exposed to DNT vapor. Inset: Depiction of **P1** and **P2** films exposed to DNT vapor for 0 s and 5 s. (**b**) Graphical representation of fluorescence-quenching ratios of **P1** films to TNT and DNT. Reproduced with permission from reference [28]. Copyright © Elsevier 2019.

Liu and coworkers reported the three-component conjugated polymers **P4** and **P5** (Figure 8) and their application as super-rapid-response fluorescent probes to DNT vapor [28]. **P4** and **P5** thin films demonstrated selectivity toward DNT vapor over other common explosives (Figure 9). The authors performed fluorescence-quenching experiments in the vapor phase only. The fluorescence-quenching ratios of **P4** and **P5** to DNT reached 93% and 96% in 5 s, respectively. In addition, **P4** and **P5** thin films have comparable quenching efficiency toward DNT and TNT, while **P4** shows remarkably higher quenching efficiency toward other analytes compared to **P5**. The authors attributed such behavior to the possible stacking of pyrene units, which is easier to achieve compared to anthracene ones. Therefore, **P4** can form looser films to increase the diffusion of analyte molecules, thus enhancing the sensing performance. The higher selectivity toward DNT and TNT could be explained by the higher vapor pressure of these analytes in comparison with other less volatile explosives. **P4** and **P5** demonstrated a fast response time: that is, after 5 s of exposure to DNT vapor, 93% and 96% of the fluorescence was quenched, respectively (Figure 10a,b). The fluorescence-quenching ratios of **P4** and **P5** films to TNT were found to be only 46% and 43% in 5 s, respectively. The reusability of **P4** and **P5** films was also investigated. They demonstrated almost the same level of fluorescence-quenching performance (93% and 94% after exposure to DNT vapor for 5 s) after three cycles of quenching/recovery.

### 2.2. Conjugated Microporous Polymers 

Porous polymers have a large specific surface area, which has unique advantages in the fluorescence detection of explosives. This allows for an improved mass-transfer capability so that explosives in solution or in the gaseous state can be pre-concentrated within the polymer, increasing the concentration of nearby fluorophores. On the other hand, it can promote the diffusion of explosive molecules into the pores of the polymer and provide a full-contact interface for the interaction of explosive molecules with fluorophores, thereby improving the detection sensitivity. In this case, the determination of porosity properties, such as the Brunauer, Emmett, and Teller surface area (S_BET_), pore volume and size, micropore volume ratio (V_micro_/V_total_), etc., plays an important role in the estimation of a polymer as a sensor for any analyte, including NACs. Therefore, the control and tuning of porosity and surface parameters are of great importance and can be achieved by choosing certain building blocks and types of implemented organic reactions. In addition, porous polymers can be applied in a field of molecular separation for the selective extraction of an analyte from a mixture, followed by recyclization by washing, centrifuging, etc., with subsequent reuse, which is very practical.

**Figure 11:**
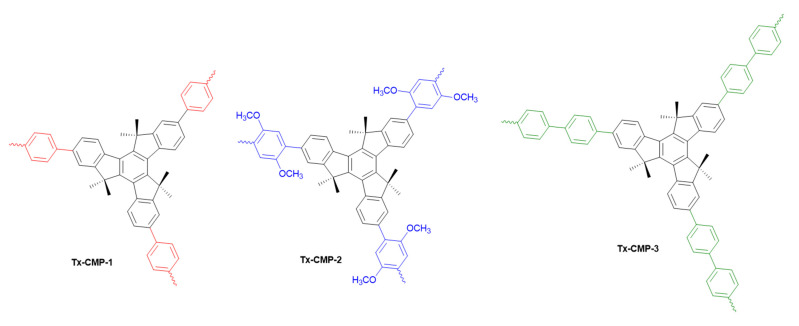
Structures of **Tx-CMPs**.

**Figure 12:**
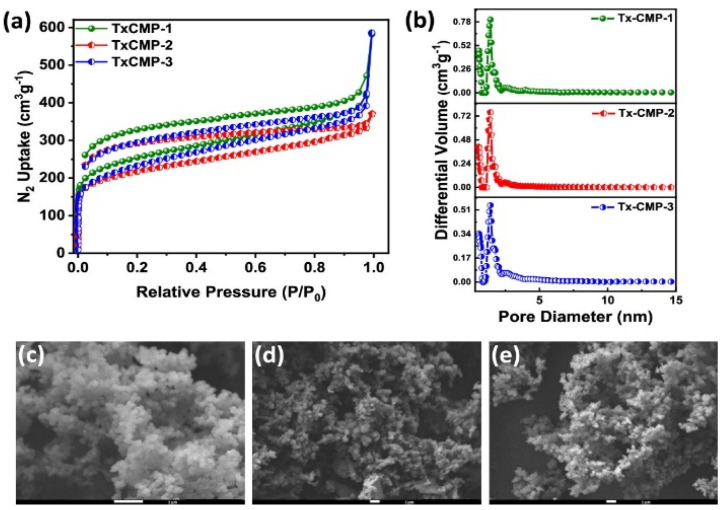
(**a**) Nitrogen adsorption and desorption isotherms of **Tx-CMPs**. (**b**) Pore-size distribution curves of **Tx-CMPs**. (**c**–**e**) FE-SEM images of **Tx-CMP-1**, **Tx-CMP-2**, and **Tx-CMP-3**, respectively. Reproduced with permission from reference [29]. Copyright © ACS Publications 2022.

Nailwal et al. designed [29] and developed three luminescent truxene-based CMPs (**Tx-CMPs**) via the Suzuki–Miyaura cross-coupling reaction (Figure 11). All **Tx-CMPs** exhibited a high BET surface area, S_BET_ = 788–915 m^2^ g^−1^ (based on nitrogen gas uptake isotherms, Figure 12a), and good thermal stability. Based on nonlocal density functional theory (NLDFT), **Tx-CMPs** mainly possess a pore size of 1.38–1.41 nm (Figure 12b) with a pore volume of 0.57–0.90 cm^3^ g^−1^, accompanied by the formation of aggregates of small globular particles with an amorphous character (Figure 12c–e). It should be noted that a negligible presence of the palladium metal residue was detected by SEM−EDS. Due to the extended aromatic conjugation system based on fluorene units and flexible aromatic linkers, **Tx-CMPs** avoid the ACQ effect and demonstrate intense luminescence. All **Tx-CMPs** were insoluble in all tested organic solvents, with the most well-dispersed suspensions of **Tx-CMPs** obtained in THF, which was chosen as the solvent for further fluorescence experiments. 

**Figure 13:**
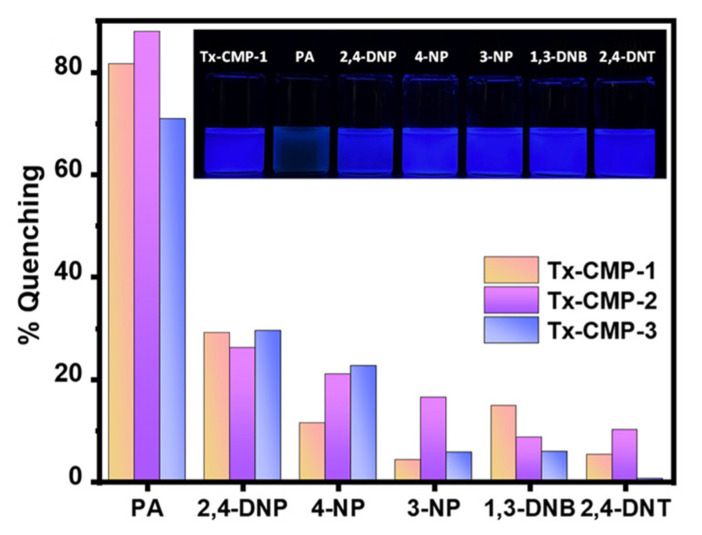
Graphical representation of fluorescence-quenching efficiency of different nitroaromatic compounds (100 μM) toward **Tx-CMP-1**, **Tx-CMP-2**, and **Tx-CMP-3** in THF. Reproduced with permission from reference [29]. Copyright © ACS Publications 2022.

Among various nitroaromatic explosives, **Tx-CMPs** demonstrated a selective luminescence turn-off sensing response to PA with *K*_SV_ values in the range of 2.39–7.35 × 10^4^ M^−1^. For comparison, K_SV_ values for other nitroaromatics (2,4-DNP, 4-NP, 3-NP, 1,3-DNB, and 2,4-DNT) were in the range of 0.48–4.15 × 10^3^ M^−1^, which is more than 10 times less, accompanied by a significantly lower quenching efficiency (Figure 13). The LODs for **Tx-CMP-1**, **Tx-CMP-2**, and **Tx-CMP-3** were calculated at 1.86, 4.05, and 2.11 nM, respectively. According to time-resolved fluorescence quenching experiments, negligible changes in the average lifetime values were observed (τ_av_ = 0.39 ns for **Tx-CMP-1**), suggesting a static quenching process in this case. Based on the HOMO–LUMO distribution for **Tx-CMPs** and PA, the authors declared that the quenching process occurred by the PET mechanism, with the largest difference between the LUMO of the sensor and the LUMO of the analyte calculated for **Tx-CMP-2**, exhibiting the most efficient quenching of luminescence.

**Figure 14:**
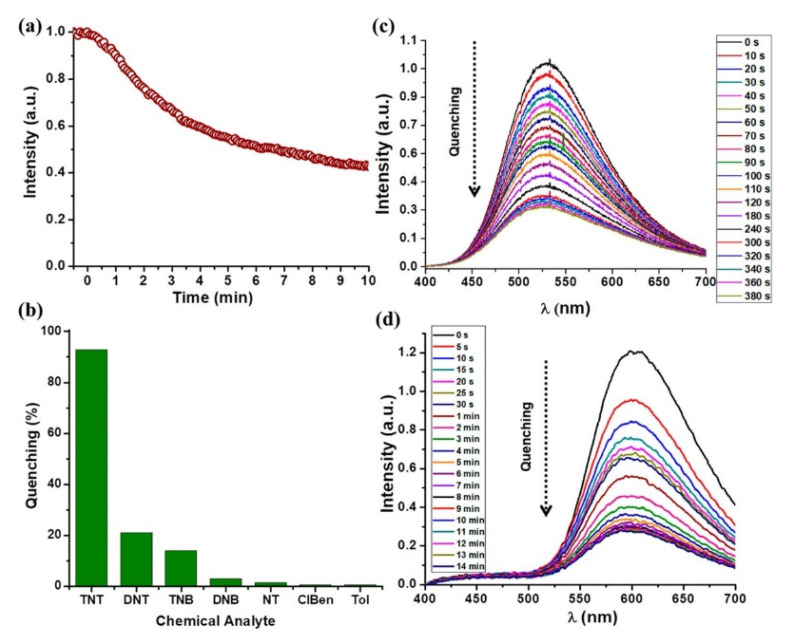
(**a**) Graphical representation of fluorescence quenching of **PTPETCz** film in presence of TNT vapor (10 ppb); (**b**) emission quenching of **PTPETCz** films after exposure to various analyte vapors: TNT (33 ppb, 3 min), TNB (10 ppb), 1,3-DNB (47 ppb), 2,4-DNT (saturated vapor), NT, chlorobenzene (ClBen), and toluene, exposure time 5 min. (**c**) Fluorescence intensity change with time in presence of TNT vapor (33 ppb) for **PTPETCz** films of ca. 61 nm thickness and (**d**) fluorescence intensity change with time in presence of TNT vapor (0.2 ppm) for **PTPEFLCz** films. Reproduced with permission from reference [30]. Copyright © ACS Publications 2018.

**Figure 15:**
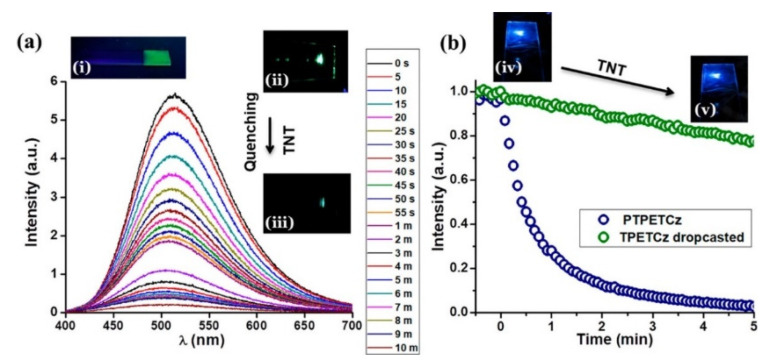
(**a**) **PTPETCz** emission decreasing during exposure to TNT vapor (33 ppb) over time. Inset: Illuminated **PTPETCz** thin films on ITO (**i**) under UV lamp and (**ii**), (**iii**) before and after exposure to TNT under a laser source, respectively. (**b**) Fluorescence quenching of **PTPETCz** films (blue) with time in presence of TNT vapor (33 ppb) compared to the fluorescence quenching of a drop-casted monomer film (**TPETCz**) when exposed to TNT vapor (0.2 ppm). (Inset) Illuminated **TPETCz** film (**iv**) before and (**v**) after TNT exposure. Reproduced with permission from reference [30]. Copyright © ACS Publications 2018.

Mothika et al. reported the electrochemical generation of three **CMP** thin films (**PTPETCz**, **PFLCz**, and **PTPEFLCz**) with high porosity and their fluorescence response to TNT vapor [30]. These three polymers exhibited different luminescence behaviors arising from the structures of the original monomers. Only the **PTPETCz** and **PTPEFLCz** polymers, which include an aggregation-induced emissive TPE core, demonstrated a sensing response to TNT vapor, with LODs of 10 ppb (Figure 14a) and 0.2 ppm (Figure 14d), respectively. **PFLCz** turned out to be irresponsive even under a higher TNT vapor pressure (0.2 ppm). The authors ascribed this contrasting behavior to the increased electron-accepting character of **PFLCz** due to its fluorenone units. In addition, **PTPETCz** appeared to be remarkably selective toward TNT compared to other analytes because of either a lower LUMO to initiate the PET process or the insufficient volatility of the nitroaromatic molecules (Figure 14b). Meanwhile, such low LOD values for TNT originated from the high microporosity of **CMP** films. S_BET_ values were calculated at 930 and 509 m^2^/g, with average pore diameters of 1.71 and 1.72 nm and total pore volumes of 0.40 and 0.22 cm^3^ g^−1^ for **PTPETCz** and **PTPEFLCz**, respectively, resulting in the better diffusion of TNT into the **CMP** matrix. The S_BET_ values of the thin films were lower than those of the corresponding bulk **CMP** powders synthesized via FeCl_3_-assisted chemical oxidation from the corresponding monomers due to the impact of the selected preparation method. However, thin films are more applicable to device and chip fabrication and demonstrate higher sensitivity to TNT vapor (Figure 15b). In this case, the authors estimated the role of film thickness and reported more efficient quenching at a lower thickness (15 nm over 61 nm).

**Scheme 1:**
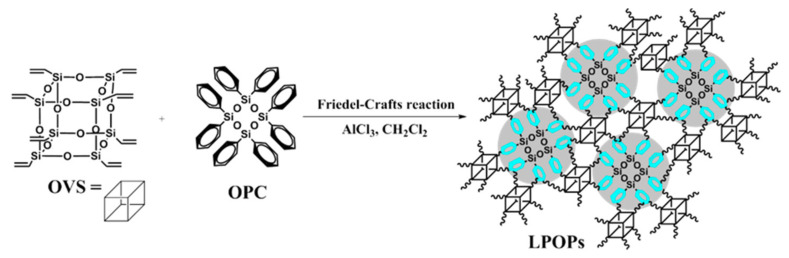
Schematic representation of the synthesis of polymers (**LPOPs**). Reproduced with permission from reference [31]. Copyright © ACS Publications 2018.

**Figure 16:**
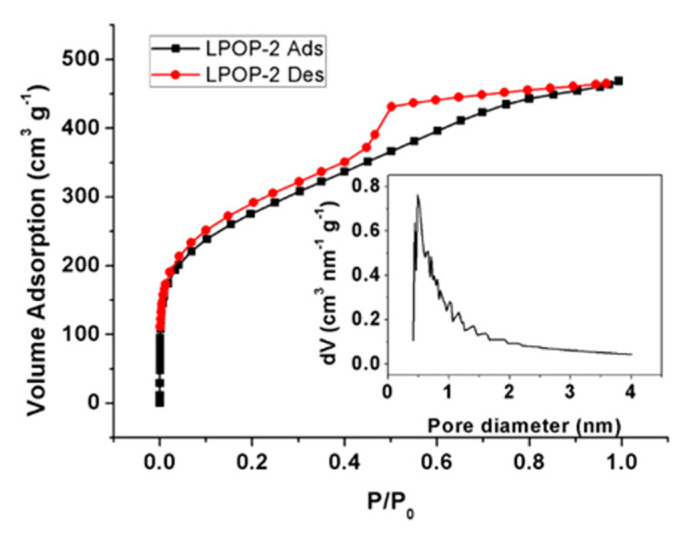
Nitrogen adsorption–desorption isotherm and pore-size distribution curve (inset) of **LPOP-2**. Reproduced with permission from reference [31]. Copyright © ACS Publications 2018.

Gou and colleagues presented siloxane-based nanoporous polymers, **LPOPs**, with a narrow pore-size distribution prepared by the Friedel–Crafts reaction of octaphenylcyclotetrasiloxane with octavinylsilsesquioxanes with different ratios (Scheme 1) [31]. **LPOPs** exhibited a narrow pore-size distribution, a monomodal micropore structure, excellent fluorescence properties, a high surface area, high thermodynamic stability, and sensing properties. According to the nitrogen adsorption–desorption isotherm and pore-size distribution (Figure 16), **LPOP-2** (the ratio of the starting reactants is 1:0.2) demonstrated a higher porosity degree with an S_BET_ of 967 m^2^g^−1^ and a corresponding micropore volume ratio (V_micro_/V_total_) of 0.94, with the monomodal nanopores centered at approximately 0.59 nm, as well as the most intense luminescence both in the solid state and in suspension at approximately 450 nm. Moreover, this intense luminescence of **LPOP-2** could be efficiently quenched by NACs, with K_SV_ constants of 2.32 × 10^4^, 1.63 × 10^4^, and 2.42 × 10^4^ M^−1^ for NT, DNT, and TNT, respectively. The authors revealed the synergetic effect of dynamic adsorption, electron transfer, and electrostatic interaction between the nanoporous materials and analytes. In addition, Gou and colleagues demonstrated the quenching of the luminescence of **LPOPs** by using paper strips. 

**Scheme 2:**
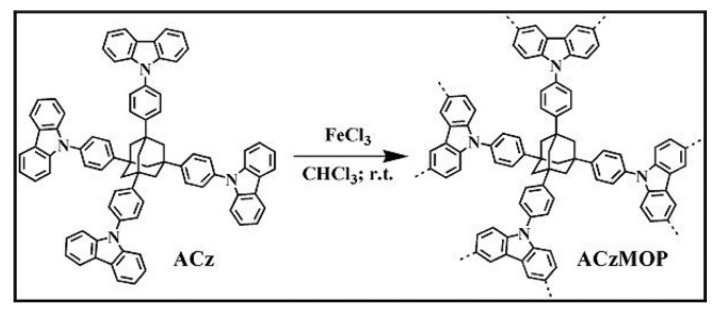
Synthetic approach for **ACzMOP**.

**Figure 17:**
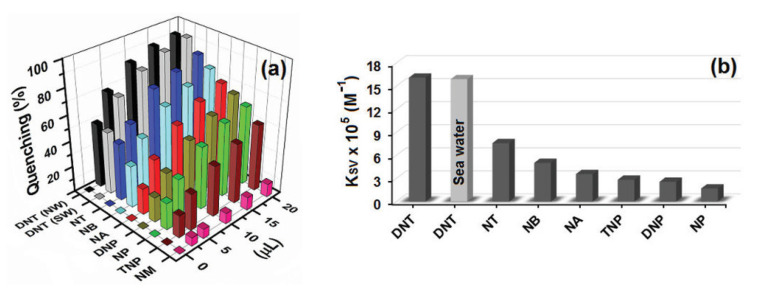
(**a**) Fluorescence quenching (%) results for different analytes. (**b**) Nitroaromatics’ Stern–Volmer quenching constant (NW = normal water; SW = sea water). Reproduced with permission from reference [32]. Copyright © Wiley 2021.

**Figure 18:**
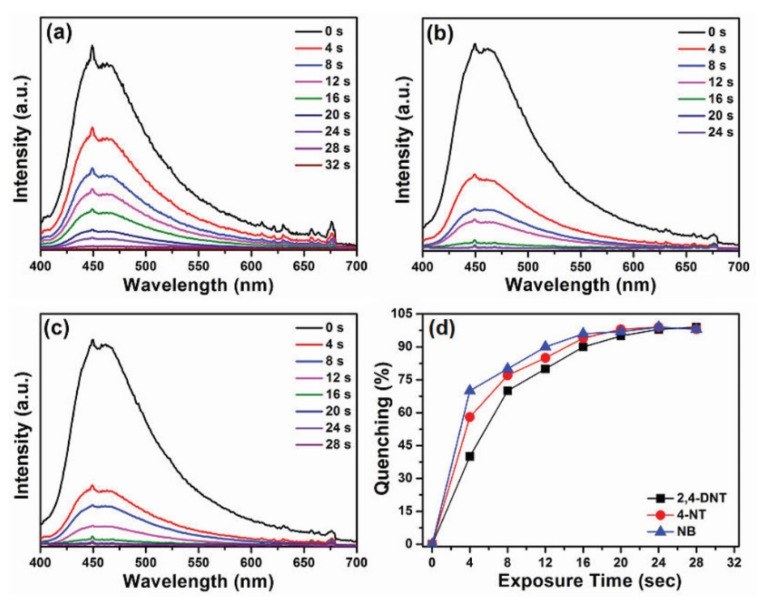
Studies of fluorescence quenching of **ACzMOP** thin layer (time-dependent) when exposed to saturated vapor of (**a**) DNT, (**b**) NT, and (**c**) NB and (**d**) fluorescence quenching (%) comparison by corresponding analytes. Reproduced with permission from reference [32]. Copyright © Wiley 2021.

Manna and co-authors developed the adamantane- and polycarbazole-based CMP **ACzMOP** for the detection of NACs in aqueous and gaseous phases (Scheme 2) [32]. **ACzMOP** demonstrated fluorescence sensitivity to NT, NB, TNP, DNP, NP, and NA analytes with high K_SV_ values, especially toward DNT, with a K_SV_ of approximately 1.6 × 10^6^ M^−1^ and a quenching efficiency of 70% (Figure 17). Moreover, these K_SV_ constants were obtained in aqueous media; K_SV_ for DNT was calculated in both normal and seawater media and indicated no influence of the type of aqueous medium (Figure 17b). The LOD was found to be 0.32 ppb for DNT, which is well below the maximum permissible limit of 2 ppb in drinking water. In addition, the authors characterized the quenching mechanism as highly dynamic in nature because of the reduction in the fluorescence lifetime upon the addition of different concentrations of DNT. With high values of S_BET_ of 1568 m^2^g^−1^, total pore volume of 1.58 cm^3^ g^−1^, and pore diameter around 1.17 nm (for the major fraction), **ACzMOP** exhibited high sensitivity to vapors of NACs such as DNT, NT, and NB, with quenching efficiencies of 95%, 99%, and 99%, respectively, after a 20 s exposure time (Figure 18). As can be seen, despite differences in vapor pressure, DNT, NT, and NB demonstrated comparable quenching efficiencies for **ACzMOP**, which led the authors to speculate about the negligible role of vapor pressure, making it possible to detect TNT vapor. The structural similarity between DNT and TNT should facilitate this as well. In addition, the process of reversible “ON-OFF” switching was also demonstrated for an **ACzMOP** thin film. After 30 min in a laboratory vacuum oven at 50 °C, the same fluorescence intensity was restored, allowing this process to repeat for many cycles. Moreover, the possibility of detecting NACs by using paper strips with **ACzMOP** was shown. In addition, the authors successfully applied **ACzMOP** for the aqueous phase separation of nitroaromatics with high efficiency due to its very high porosity. 

### 2.3. Branched Polymers 

Hyperbranched polymers are composed of three parts: dendritic links, linear links, and end links. Dendritic units and linear units are randomly distributed over the macromolecular backbone, which leads to randomly branched structures and the polydispersity of the molecular weight. At the same time, hyperbranched polymers have a large number of modifiable end groups, which can conveniently be further adjusted for the fluorescent and sensory properties of the polymers through the functionalization of the end groups. However, in this case, the polymer performance depends strongly on the nature and number of end groups, as they are not part of the polymer system. 

**Figure 19:**
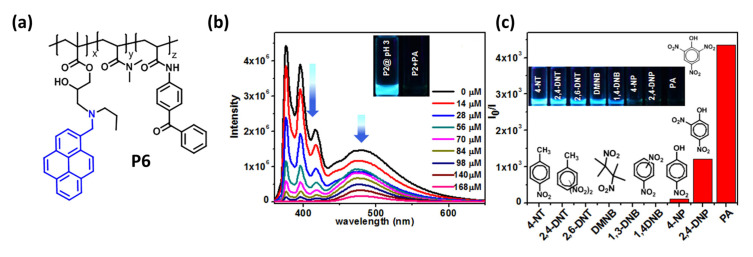
(**a**) Structure of **P6**. (**b**) Changes in emission spectra of **P6** (2.1 × 10^−7^ M pyrene units) on addition of **PA** (0–0.168 mM) at pH 3 in aqueous media and (**c**) **P6** selectivity in water with other structurally similar analytes. Reproduced with permission from reference [33]. Copyright © ACS Publications 2018.

**Figure 20:**
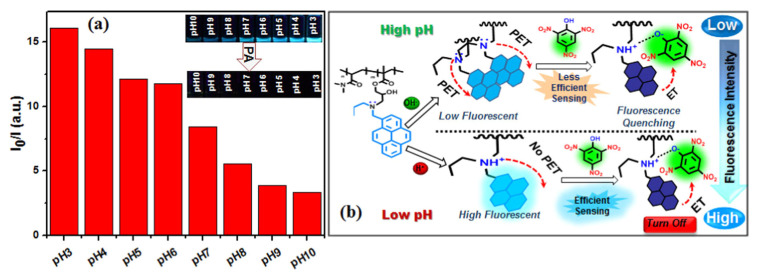
(**a**) Emission quenching extent upon PA (0.168 mM) addition at various pH; inset: **P6** (2.1 × 10^−7^ M pyrene units) before and after the addition of PA. (**b**) Mechanistic pathway of pH-tunable detection sensitivity of **P6** upon PA addition in H_2_O. Reproduced with permission from reference [33]. Copyright © ACS Publications 2018.

Gupta and Lee [33] reported recyclable polymeric thin films based on a pyrene-derived polymeric probe, **P6** (Figure 19a), for the selective detection and separation of PA with K_SV_ = 7.75 × 10^4^ M^−1^ in aqueous media (Figure 19b,c). At lower pH, trialkylamine groups could be protonated, turning off the PET process and amplifying the luminescence intensity (Figure 20). This provides a favorable way to sense PA via quenching of this amplified luminescence. Because of the nonlinear nature of the K_SV_ plot, the lower-lying LUMO energy level of PA compared to **P6**, and the overlap between the fluorescence spectra of **P6** and the absorption spectra of PA, the authors declared a combination of PET and FRET mechanisms. The LOD for PA was determined to be 56 μM. Based on the **P6** polymer, the corresponding **P2** film was fabricated with higher sensitivity to PA. The authors reported that only 11.4 attograms (10^−14^ M) was required to initiate fluorescence quenching by PA. They also attributed this amplified fluorescence quenching of the **P6** film to the π–π stacking of the pyrene units, which enabled an exciton-hopping process via intermolecular electronic coupling, facilitating long-range exciton diffusion in the well-assembled solid state. The authors demonstrated the possibility of applying the **P6** film to separate PA from a mixture of different nitroaromatics in a MeCN solution.

**Figure 21:**
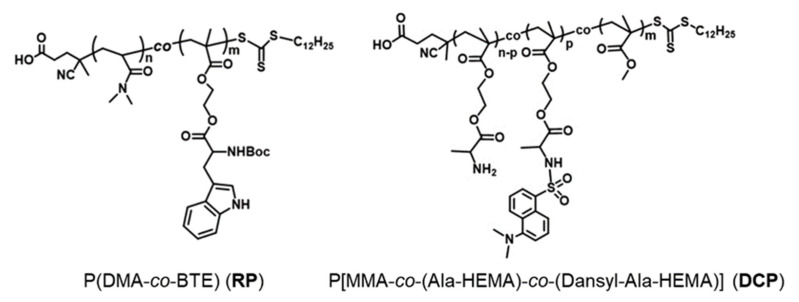
Chemical structures of copolymers **RP** and **DCP**.

**Figure 22:**
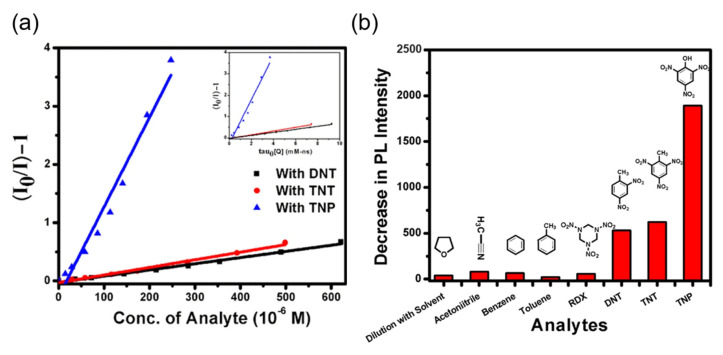
(**a**) Stern–Volmer plots for PA, DNT, and TNT. (**b**) Histogram for **DCP** selectivity test. Reproduced with permission from reference [34]. Copyright © Springer Nature 2019.

Kumar et al. reported the synthesis and multimodal sensing applications of an emissive alanine-based dansyl-tagged copolymer P(MMA-*co*-Dansyl-Ala-HEMA) (**DCP**) (Figure 21) synthesized by RAFT copolymerization [34]. This polymer demonstrated high sensitivity and selectivity toward PA, as well as toward other NAC explosives, such as DNT and TNT, with lower quenching efficiency in THF solutions (Figure 22a,b). The **DCP** polymer demonstrated K_SV_ values of 1.1 × 10^3^ M^−1^ and 1.3 × 10^3^ M^−1^ for DNT and TNT and a large value of 1.6 × 10^4^ M^−1^ for PA. The authors characterized the mechanism of quenching as a combination of static and dynamic processes based on fluorescence up-conversion and time-resolved fluorescence data. In addition, the vapor sensing of NACs by a 20 nm thin film of **DCP** was demonstrated, with quenching efficiencies in the order DNT > TNT > PA associated with the higher vapor pressure of DNT over TNT and PA. 

**Figure 23:**
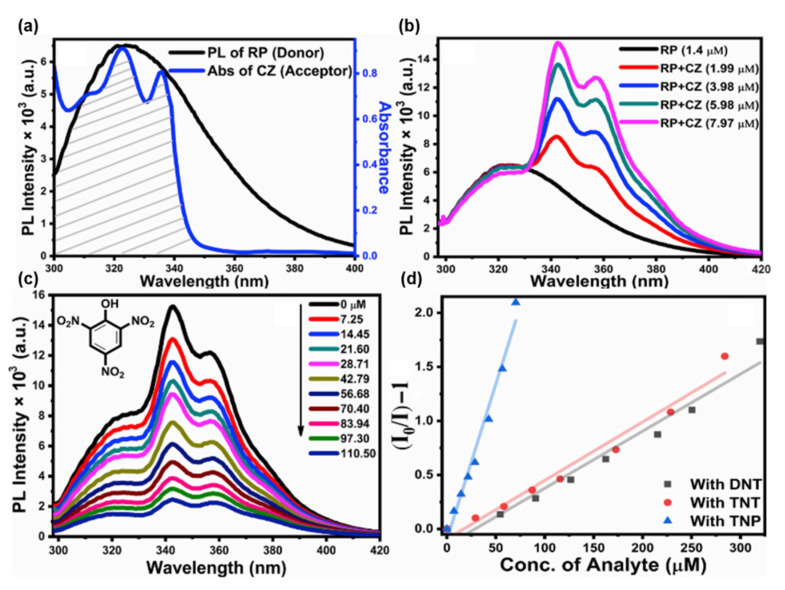
(**a**) Fluorescence of **RP** and absorption of CZ (spectral overlap); (**b**) FRET signal for donor–acceptor pair at the excitation wavelength of donor (291 nm); (**b**,**c**) fluorescence quenching of FRET pair with addition of PA. (**d**) Stern–Volmer plots for PA, DNT, and TNT. Reproduced with permission from reference [34]. Copyright © Springer Nature 2019.

In addition, they demonstrated the possibility of detecting NACs with a polymer–small-molecule donor–acceptor FRET pair, using the tryptophan copolymer poly[(N,N-dimethylacrylamide)-co-(Boc-tryptophan methacryloyloxyethyl ester)] (also known as (P-[DMA-co-(Boc-Trp-EMA)])) (**RP**) (Figure 21) as the donor and carbazole (CZ) as the acceptor. This RP:CZ system demonstrated high sensitivity to NACs, with K_SV_ = 2.9 × 10^4^ M^−1^, 5.3 × 10^3^ M^−1^, and 5.7 × 10^3^ M^−1^, as well as LOD values of 1.4 μM, 6.4 μM, and 5.9 μM for PA, DNT, and TNT, respectively. This unique and high-sensitive detection works based on the FRET process, where the FRET mixture is excited at the donor’s (**RP**) characteristic wavelength (291 nm). This excitation energy of the donor is resonantly transferred to the acceptor because of the great overlap between the fluorescence of **RP** and the excitation of CZ (Figure 23a), followed by a dramatic increase in the PL emission of CZ at 325–425 nm (Figure 23b). This emission is further weakened with the increase in the DNT, TNT, and TNP concentrations (Figure 23c for PA). A time-resolved spectroscopic study confirmed the occurrence of the FRET process with a considerably good energy transfer efficiency ϕ_E_ = 35% and a decrease in the average lifetime of PA from 1.6 to 1.0 ns. The authors also calculated the Förster critical distance *R*_0_ and the distance between donor and acceptor molecules *r* at 25.51 Å and 28.28 Å, respectively, which are in the range of 10–100 Å and applicable to the FRET process.

**Figure 24:**
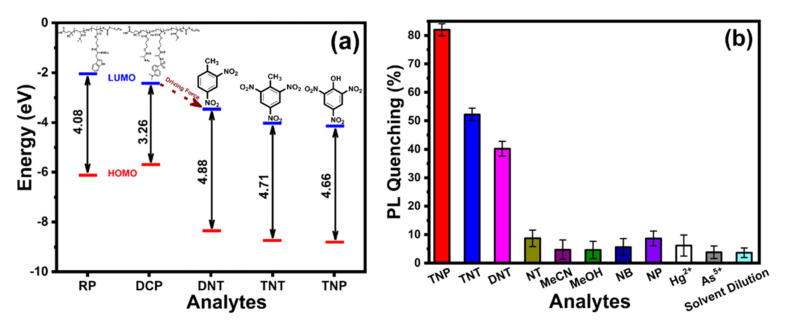
(**a**) Energy levels for **RP**, **DCP**, and the analytes and (**b**) quenching efficiency by different NACs and other analytes. Reproduced with permission from reference [35]. Copyright © ACS Publications 2021.

Moreover, in their subsequent research [35], the authors reported the polymer–polymer donor–acceptor FRET pair **RP:DCP**, with the copolymer **RP** as the donor and the copolymer **DCP** as the acceptor (Figure 21). The sensitivity of this copolymer toward NACs is based on the same FRET pair principle as **RP**:CZ, with the dominance of the PET quenching mechanism (Figure 24a). Kumar and colleagues demonstrated that changing the excitation source for **DCP** from an outer source to the FRET approach is favorable for NAC detection. The K_SV_ values appeared to be comparable to those of the **RP**:CZ FRET pair and were 2.2 × 10^4^ M^−1^, 6.3 × 10^3^ M^−1^, and 7.6 × 10^3^ M^−1^ with LODs of 0.4 μM (0.1 ppm), 5.4 μM (0.98 ppm), and 2.3 μM (0.53 ppm) for PA, DNT, and TNT, respectively. However, this system demonstrated values approximately 6 orders of magnitude higher than the reported K_SV_ values for the **DCP** polymer, indicating the key role of the FRET pair in the quenching of NACs. In addition, the authors provide deep insight from optical, time-resolved spectroscopic, and ultrafast transient absorption studies to establish the mechanistic aspects of the interactions between **PR:DCP** and NACs. They demonstrated the selectivity of the **RP:DCP** pair toward PA, DNT, and TNT over other analytes (Figure 24b).

## 3. Modern Accents/Trends in the Development/Research of Polymers/Polymer-Based Materials for the Detection of NACs

### 3.1. Polymer Nanoparticles for Detection in Aqueous Media 

One of the main disadvantages of polymer-based materials is their flat surface, which prevents the deep penetration of NACs into the polymer body, especially on hard surfaces. When π-conjugated polymers are converted into nanostructures, the properties of the polymer are enhanced by additional nanoscale characteristics, including the ability to functionalize the inside or outside of nanostructures and increase solubility in various solvents, including water [36]. Conjugated nanostructured polymers have high quantum yields, molar extinction coefficients, brightness, and photostability. Due to these properties, polymer-based nanoparticles (**PNs**) have found wide applications in optoelectronics and biomedicine [37]. Small **PNs** with high specific surface areas for maximum sensitivity are considered promising probes for detecting nitro-containing analytes.

**Scheme 3:**
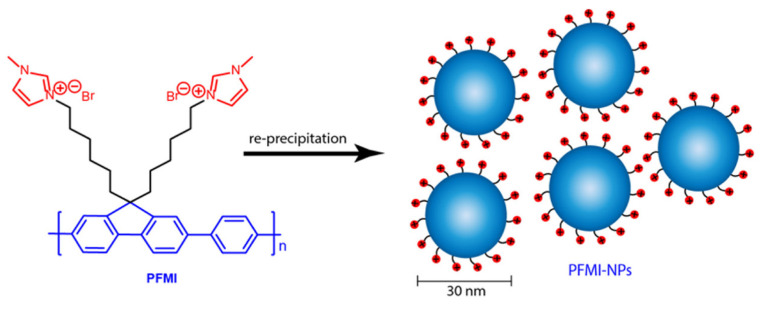
Preparation of **PFMI-NPs**.

**Figure 25:**
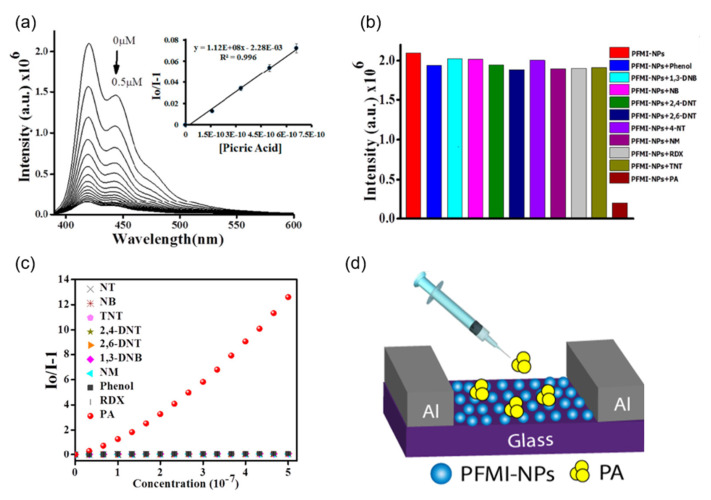
(**a**) Emission spectra of **PFMI-NPs** with variable concentrations of PA in aqueous media. Inset: Stern–Volmer plot at lower PA concentrations. (**b**) Effect of different nitro-explosives (5 × 10^−7^ M) on the emission of **PFMI-NPs**. (**c**) Stern–Volmer plots for various nitro-explosives. (**d**) Two-terminal sensor device (schematic representation). Reproduced with permission from reference [38]. Copyright © ACS Publications 2015.

Malik et al. fabricated a fast and sensitive platform based on conjugated polymer nanoparticles (**PFMI-NPs**) with an average particle size of 30 nm for the selective detection of PA (Scheme 3) [38]. With the pendant cationic imidazolium groups attached to the side chains of the PFMI polymer, it became water-soluble and obtained a specific recognition site for PA in polar solvents. This led **PFMI-NPs** to demonstrate higher sensitivity for PA over other NACs, with K_SV_ = 1.12 × 10^8^ M^−1^ (Figure 25a,b) and a LOD of 30.99 pM/7.07 ppt in water. The exceptional selectivity and sensitivity toward PA remained unchanged in the presence of a variety of cations and anions yet were reduced by 50% in a 1 M NaCl aqueous solution, demonstrating sensitivity to PA at relatively high electrolyte concentrations in aqueous media. Because of the nonlinear Stern–Volmer plot at higher concentrations of PA (Figure 25c), the authors described the quenching mechanism as a combination of the “molecular-wire effect”, electrostatic interactions, and PET and possibly RET mechanisms. Moreover, the authors fabricated a two-terminal sensor device for PA vapor detection (Figure 25d) with high sensitivity and selectivity, a high signal response (6 s), and fast recovery.

**Figure 26:**
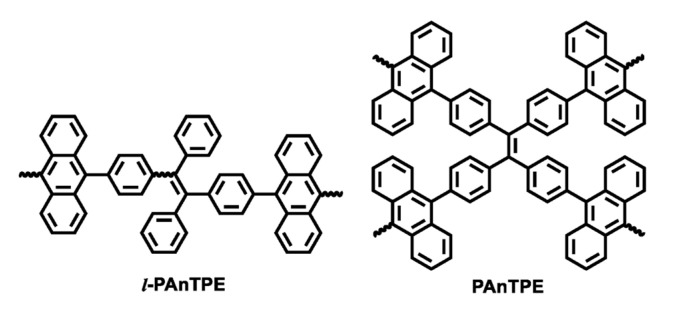
Structures of ***l*-PAnTPE** and **PAnTPE** nanoparticles.

**Figure 27:**
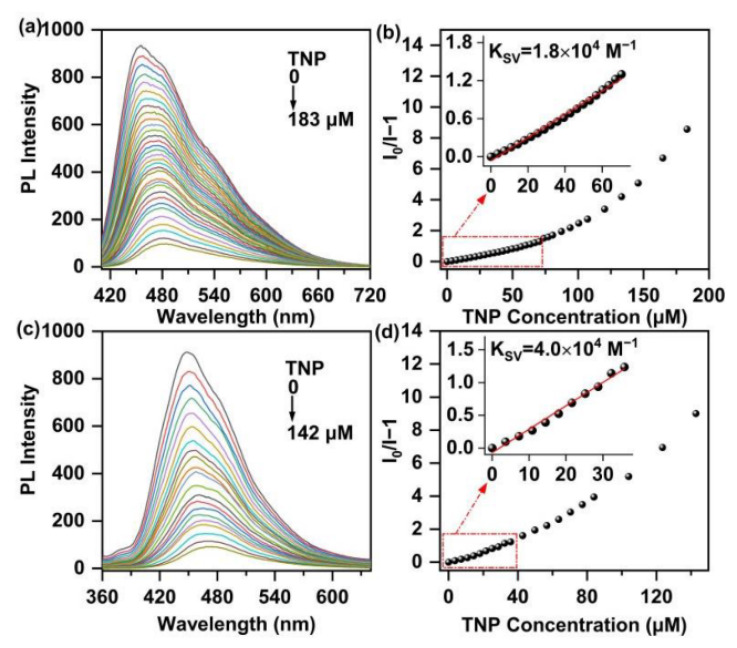
(**a**) Emission spectra of ***l*-PAnTPE**; (**b**) S–V plots of PL intensity (I_0_/I − 1) vs. TNP concentration of ***l*-PAnTPE**; (**c**) emission spectra of **PAnTPE**; (**d**) S–V plots of PL intensity (I_0_/I − 1) vs. TNP concentration of **PAnTPE**. Reproduced with permission from reference [39]. Copyright © MDPI 2022.

**Figure 28:**
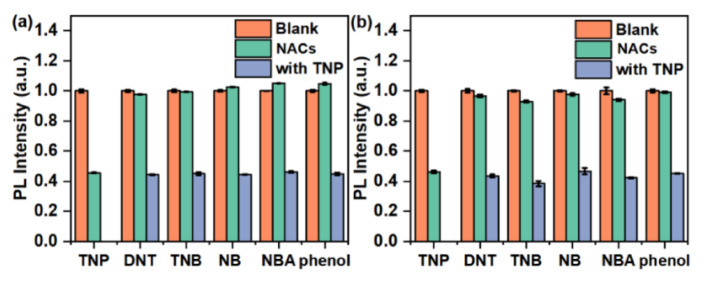
The quenching degree of ***l*-PAnTPE** (**a**) and **PAnTPE** (**b**). Reproduced with permission from reference [39]. Copyright © MDPI 2022.

Ouyang and co-authors reported ***l*-PAnTPE** and **PAnTPE** polymer nanoparticles based on anthracene and tetraphenylethene units (Figure 26) for the detection of nitroaromatics in aqueous media [39]. Despite the difference in molecular structure, both *l*-**PAnTPE** and **PAnTPE** polymers demonstrated similar fluorescence emission with maxima at 455 and 448 nm, respectively. However, **PAnTPE** exhibited higher efficacy for the quenching of PA in water, with a K_SV_ of 4.0 × 10^4^ M^−1^, compared to ***l*-PAnTPE**, with a K_SV_ of 1.4 × 10^4^ M^−1^ (Figure 27), as well as higher sensitivity, with a LOD of 439 nM compared to 663 nM. The better sensing activity of **PAnTPE** is due to its surface parameters. Both particles possessed an average size of 73 nm and spherical morphology. However, porosity with a pore size centered at ca. 3.5 nm and an S_BET_ of 49 m^2^/g for **PAnTPE** was observed, thus facilitating contact and the adsorption/diffusion of the analyte in the polymer. *l*-**PAnTPE** possessed no porosity due to its linear molecular structure. K_SV_ values for other NACs were much lower, while the presence of different cations and anions had an insignificant effect, indicating high selectivity toward PA (Figure 28). The authors revealed a static mechanism of quenching based on time-resolved fluorescence decay data, a prerequisite for the PET mechanism due to higher LUMO values of ***l*-PAnTPE** and **PAnTPE** (−2.68 and −2.53 eV, respectively, vs. −3.90 eV for PA), as well as for the FRET mechanism due to the overlaps between the absorption spectra of PA and the fluorescence spectra of ***l*-PAnTPE** and **PAnTPE**. The authors also note the possibility of the appearance of an inner-filter effect because of overlaps between the excitation spectra of PA and the fluorescence spectra of ***l*-PAnTPE** and **PAnTPE**. 

**Figure 29:**
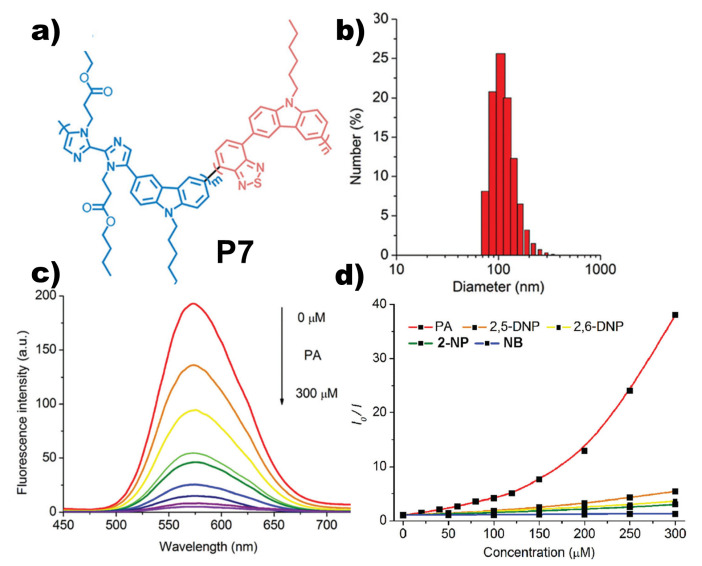
(**a**) 2,2′-Biimidazole-containing polymer **P7** (m/n = 9/1) (chemical structure); (**b**) data of dynamic light scattering (DLS) of the nanoparticles from **P7** in aqueous solution; (**c**) fluorescence spectra in presence of increasing concentrations of PA; (**d**) Stern–Volmer plots of **P1** for various nitroaromatics. Reproduced with permission from reference [40]. Copyright © Royal Society of Chemistry 2018.

Wang and colleagues reported aggregation-enhanced FRET-active conjugated polymer nanoparticles, **P7** (Figure 29a), with an average diameter of 117 nm (Figure 29b) and orangish-red fluorescence emission with a maximum at 572 nm in 1,4-dioxane-water media (f_w_ = 92%) and applied them for PA sensing in an aqueous solution [40]. **P7** exhibited high sensitivity and selectivity toward PA (Figure 29c), with K_SV_ = 3.4 × 10^4^ M^−1^, a LOD of 0.51 μM, and, according to the authors, a conjectural PET mechanism of quenching. Other NACs demonstrated much lower efficiency (Figure 29d). The presence of different cations and anions did not influence the quenching performance of **P7**.

**Scheme 4:**
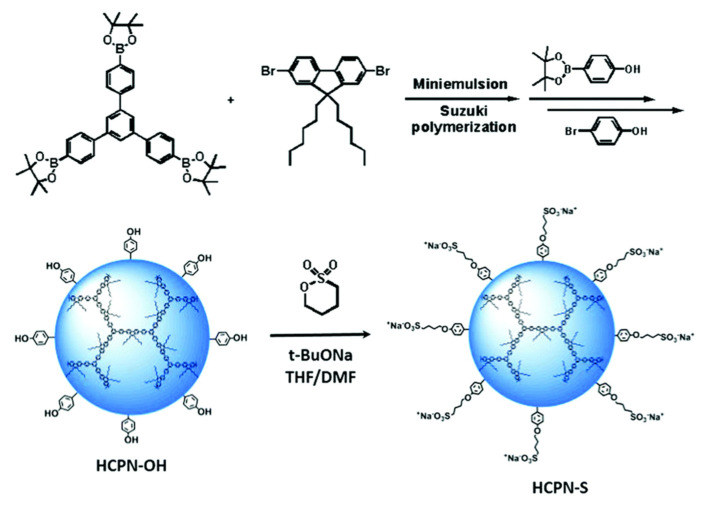
Synthetic approach for **HCPN-S**. Reproduced with permission from reference [41]. Copyright © Royal Society of Chemistry 2017.

**Figure 30:**
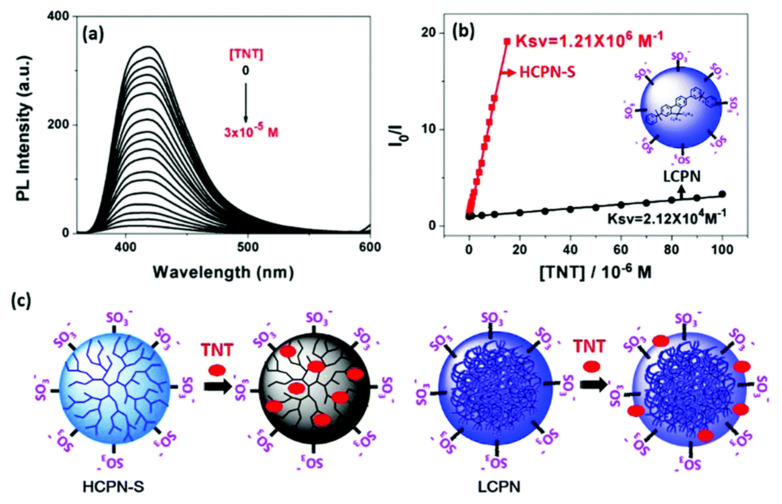
(**a**) Fluorescence response of **HCPN-S** dispersion during addition of TNT. (**b**) Stern–Volmer plots of **HCPN-S** at 419 nm and **LCPN** at 394 nm. (**c**) Proposed mechanistic pathway for different sensitivities between **HCPN-S** and **LCPN**. Reproduced with permission from reference [41]. Copyright © Royal Society of Chemistry 2017.

**Figure 31:**
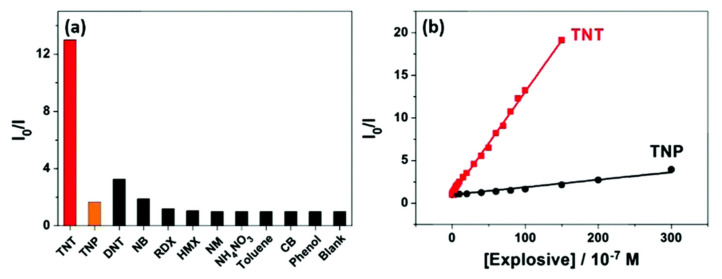
(**a**) Fluorescence response of **HCPN-S** upon addition of different analytes (concentrations: 10 μM for TNT, TNP, DNT, and NB; 100 μM for other analytes). (**b**) Stern–Volmer plots of **HCPN-S** at 419 nm. Reproduced with permission from reference [41]. Copyright © Royal Society of Chemistry 2017.

Wu and co-authors presented water-dispersible hyperbranched conjugated polymer nanoparticles with sulfonate terminal groups (**HCPN-S**) for the amplified fluorescence sensing of TNT traces in an aqueous solution (Scheme 4) [41]. This polymer was prepared by Suzuki coupling polymerization in an oil-in-water miniemulsion followed by post-functionalization with the attachment of hydrophilic sulfonate terminal groups to the hydrophobic hyperbranched conjugated polymer core. The resulting NPs had an average diameter of around 25 nm and demonstrated a high fluorescent response to TNT in aqueous media, with K_SV_ = 1.21 × 10^6^ M^−1^ and a LOD of 3.7 nM (0.8 ppb) (Figure 30a,b). **HCPN-OH**, which is the corresponding precursor of **HCPN-S** with OH groups, exhibited a significantly lower K_SV_ = 2.21 × 10^3^ M^−1^, determining the key role of sulfonated groups in the detection of TNT. In addition, the authors prepared linear conjugated polymer nanoparticles (**LCPNs**)—the linear analog of **HCPN-S**. These **LCPNs** demonstrated a much lower K_SV_ = 2.12 × 10^4^ M^−1^ and a LOD of 3.0 μM (Figure 30b), indicating the pivotal contribution of the nature of the hyperbranched core. The inner cavities of the core of **HCPN-S** resulted in an enhanced encapsulation ability, providing a large PET interface and a more efficient quenching performance (Figure 30c). The authors investigated the quenching performance of other NACs in water as well and observed high selectivity toward TNT (Figure 31a). Even PA, which is the most common NAC, exhibited a high K_SV_ value (8.39 × 10^4^ M^−1^), but it was more than 14-fold lower than that of TNT (Figure 31b).

### 3.2. Aggregates of Polymeric Probes 

At high concentrations of both small-molecule-based and polymeric fluorophores, in most cases, the fluorescence decreases dramatically due to such common phenomena as aggregation-induced quenching or aggregation quenching (AQ), which significantly hampers the application of most fluorescent materials for the fluorescence detection of NACs. In 2001, Tang et al. reported, for the first time, the emission of fluorophores, which did not appear in solution but appeared during aggregation, and this phenomenon was named “aggregation-induced emission” (AIE) (opposite of AQ) [42]. AIE-active polymer probes and materials have been successfully applied to detect explosives, and the number of published papers in this area has rapidly increased over the last 10 years [24,43]. The high fluorescence efficiency, synergistic effect, and large contact area in the aggregated state endow AIE polymers with a “super-amplification effect” and make them ideal candidates for constructing sensors with high sensitivity [44]. AIE sensors have been developed for ions, explosives, and biomolecules [45]. In the solid state, non-AIE-based sensors suffer from partial or complete emission quenching, which makes their practical application difficult [24,43]. The opposite of ACQ, the aggregation-induced emission (AIE) phenomenon, has arisen in the molecular engineering of luminescent materials and has generated curiosity among various scientific fields [46]. Luminogens with AIE characteristics (AIEgens) are used as an approach to overcome the problem connected with ACQ, the most popular of which is the tetraphenylethene (**TPE**) functional group.

This section provides a wide range of readers with updated information on current trends in the development and research of optical sensors based on polymer aggregates with a linear, branched, or porous structure for the detection of nitro-containing pollutants. This includes several reports comparing the sensitivity of ACQ, AIE, and AEE polymers as probes for detecting explosives under the same conditions, as well as sensors based on through-space conjugated polymers.

#### 3.2.1. Linear Conjugated Polymers 

**Figure 32:**
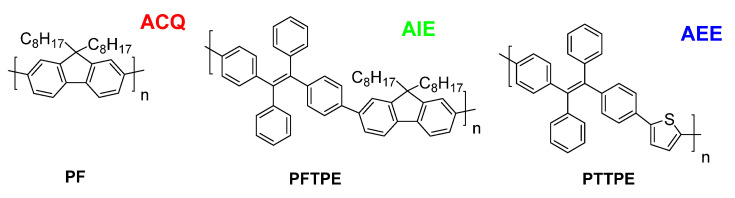
Structures of linear conjugated polymers **PF**, **PFTPE**, and **PTTPE**.

To study the relationship between the quenching mechanism of polymer sensors and the sensitivity/selectivity of the detection of nitro-containing analytes, three linear polymers were synthesized with tetraphenylethane (**TPE**), fluorene, and thiophene moieties, namely, an aggregation-caused quenching (ACQ)-active polymer (PF), an aggregation-induced emission (AIE)-active polymer (**PFTPE**), and an aggregation-enhanced emission (AEE)-active polymer (**PTTPE**) (Figure 32) [47]. Then, polyurethane foams modified with three polymers, **PU-PF**, **PU-PFTPE**, and **PU-PTTPE**, were prepared as carriers using the ultrasonic processing method.

A comparative study of polymer sensors on polyurethane foam with a three-dimensional mesh structure for the detection of explosives was carried out. It was shown that significant quenching of fluorescence was observed with the introduction of TNP solutions. All **PU-PF**, **PU-PFTPE**, and **PU-PTTPE** sensors showed excellent sensitivity to TNP solutions at various concentrations. Typically, the ACQ phenomenon results in reduced emission in the aggregate or solid form of fluorescent samples, which reduces their potential for practical applications [48]. However, the ACQ **PU-PF** polymer was shown to provide better sensory performance than polymers with AIE or AEE characteristics. Thus, the highest TNP extinguishing efficiency for **PU-PF** was 96.2%, while it was 93.5% for **PU-PFTPE** and 86.7% for **PU-PTTPE**. The probable explanation for the efficiency lies in the modification of the fluorene domain and the choice of a suitable carrier.

Although fluorene-containing chemosensors have ACQ properties due to their planarity, the active methylene (C-9) position of the fluorene core results in easy functional modification, making it suitable for use in fluorescent materials and sensors for detecting analytes [49]. For example, three conjugated polymers based on C9-functionalized fluorene domains (**CP1**, **CP2**, and **CP3**) were found to be highly sensitive for the fluorescence detection and colorimetric detection of PA in an aquatic environment [26] (Figure 2, Figure 3 and Figure 4).

**Figure 33:**
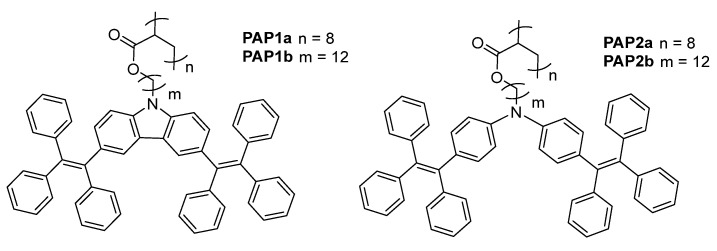
Structures of linear polymers **PAP1a–b** and **PAP2a–b**.

A number of articles present typical linear AIE polymer sensors based on **TPE** luminogens for the selective detection of 2,4,6-trinitrotoluene in solution and the gas phase. For example, Xu et al. obtained and studied four linear polyacrylate polymers, **PAP1a–b** and **PAP2a–b**, containing two AIE luminogens as side units, namely, 3,6-bis(1,2,2-triphenylvinyl)carbazole (**BTPC**) and bis(4-(1,2,2-triphenylvinyl)phenyl)-amine (**BTPPA**), in high yields via a free radical polymerization reaction in the presence of an azobisisobutyronitrile catalyst. The polymer structures are shown in Figure 33 [50]. The spectrofluorometric analysis of polymer nanoaggregates in THF/water showed that **BTPPA**-based polymers gave a more sensitive fluorescent response to nitroaromatic compounds, including 2,4,6-trinitrotoluene, with a maximum K_SV_ value of 12870 M^−1^ for **PAP2b**, than **BTPC**-based polymers.

**Figure 34:**
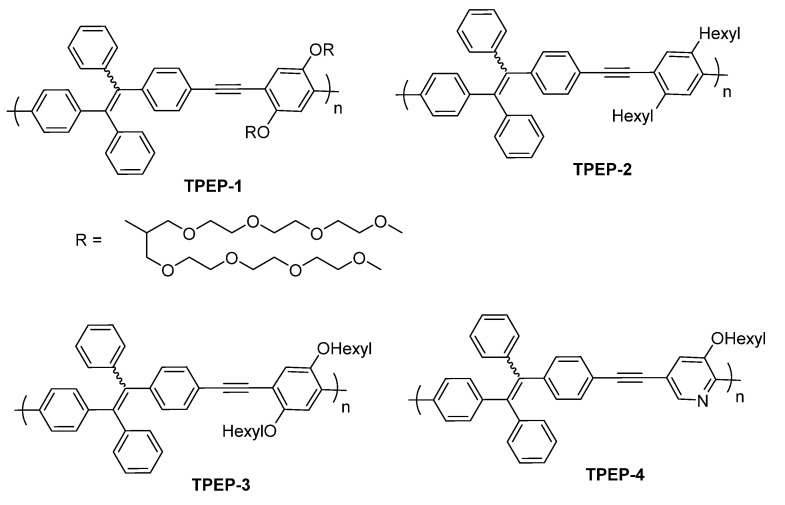
Structures of **TPEPs**.

Tetraphenylethene-based aryleneethynylene-type polymers (**TPEPs**) were synthesized through the Sonogashira coupling of diethynyl-**TPE** and three diiodobenzene struts carrying branched oligoethylene glycol, hexyl, or hexyloxy side chains (Figure 34). The presence of aggregates in a mixture of THF/water (5:95) was proved using a scanning electron microscope. The **TPEPs** were tested as a sensor array for 14 different nitroaromatic analytes and displayed fingerprint fluorescence-quenching responses. Four polymeric **TPEPs** detected and distinguished nitroarenes by quenching their fluorescence in the aqueous phase with a K_SV_ greater than 104 M^−1^ and a LOD below the ppm level for TNT [51]. 

**Figure 35:**
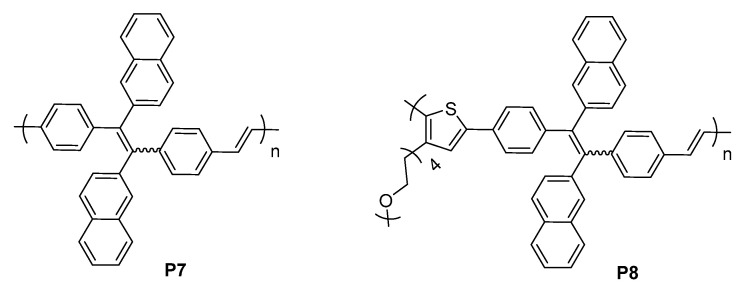
Molecular structures of **DNDPE**-based conjugated polymers.

More recently, two novel linear di(naphthalen-2-yl)-1,2-diphenylethene-based (**DNDPE**) conjugated polymers, **P7–P8**, were developed and studied (Figure 35) [52]. They possessed AEE activity due to the non-standard **DNDPE**-luminogen, good solubility, and high photostability. The emission of polymeric nanoparticles P1 in THF/water mixtures (*f*_w_ = 90 vol%) and P8 in THF/water mixtures (*f*_w_ = 80 vol%) was quenched with PA. Moreover, probe **P8** demonstrated higher sensitivity than **P7**, with K_SV_ = 9.8 × 10^5^ M^−1^.

**Figure 36:**
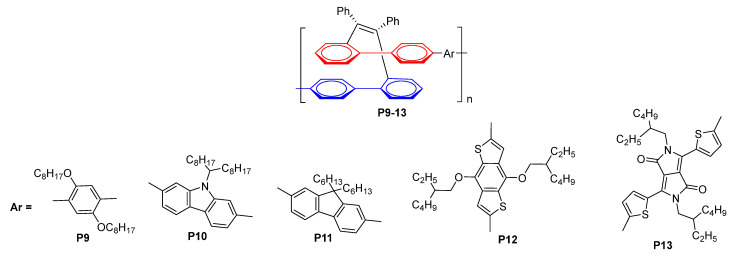
Structures of through-space conjugated polymers **P9–13**.

Through-bond conjugated polymers have been widely studied and have shown great potential in various applications due to their conductive or luminescence properties, while through-space conjugated polymers are less explored [53]. New fluorescent through-space conjugated polymers **P9–13** combining various aromatic moieties were synthesized via Pd-catalyzed Suzuki/Stille coupling reactions on the basis of the intermediate dibrom-substituted monomer (Z)-o-DBr-**BPTPE** derived from folded tetraphenylethene (**TPE**) [54]. The resulting polymers, **P9**, **P10**, and **P11**, exhibited higher fluorescence quantum yields in aggregates and films than in solutions, thus exhibiting aggregation-enhanced emission (AEE) properties. The emission peaks in THF solutions and films of **P12** and **P13** were red-shifted to 516 and 678 nm, respectively. These differences in **P12** and **P13** are attributed to the strong π–π interactions and/or dipole–dipole interactions in the aggregated state (Figure 36).

The effect of the super-amplification of emission by nanoaggregates of polymers in an aqueous medium upon the detection of TNP was proved by comparing the quenching constants in THF/water mixtures and THF. For example, the quenching constant of **P1** in the THF/water mixture (*f*_w_ = 80 vol%) was up to 552,000 M^−1^, which is much higher than that in the THF solution (78,200 M^−1^).

#### 3.2.2. AIE-Active Porous Conjugated Polymers

The integration of typical AIE luminogens into a porous organic network provides additional advantages for the detection of analytes of various natures, such as extended π-delocalization compared to a conjugated network of linear polymer sensors, which leads to efficient exciton migration. In addition, the porous structure of polymer sensors promotes the efficient diffusion of analytes, enabling specific molecular interactions within pores and allowing the design of sensors with highly sensitive and selective detection of analytes [55]. 

**Scheme 5:**
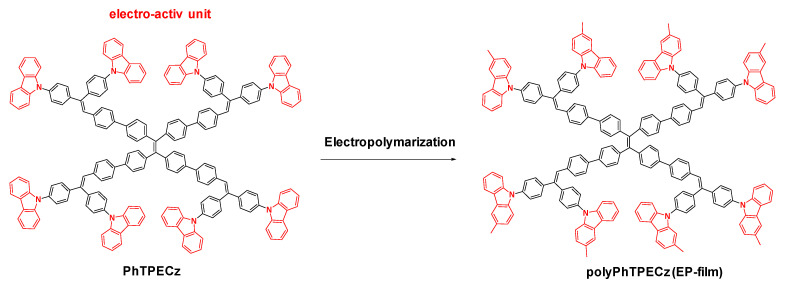
Schematic diagram of electropolymerization to form EP film (red carbazole connected to ext carbazole unit).

**Figure 37:**
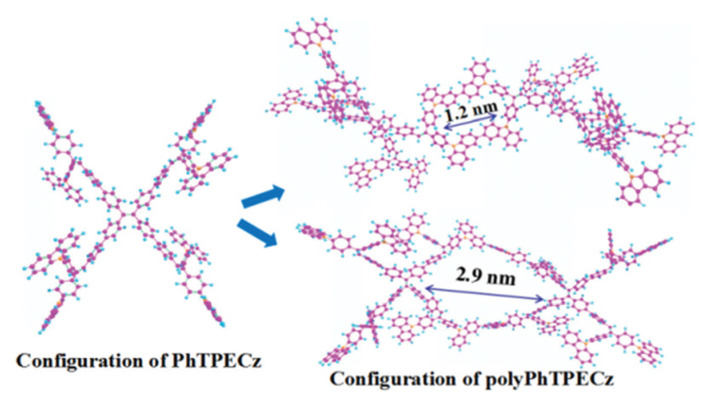
**PhTPECz** and **polyPhTPECz** (optimized structures) and diameter of pores in **polyTPECz**. Reproduced with permission from reference [56]. Copyright © Royal Society of Chemistry 2021.

Special attention from scientists is drawn to porous polymer films used as sensors for volatile organic pollutant compounds, most often obtained in situ by electropolymerization (EP). For example, polymeric porous films (EP films) were obtained in situ by electropolymerization (EP) of a new luminescent AIE-active monomer, **PhTPECz**, consisting of terphenylethylene units as the central core and eight electroactive carbazole fragments (Scheme 5, Figure 37) [56]. Based on the results of transmission electron microscopy, it was shown that for two possible twist conformations, the micropores of the resulting films were ~1.2 and 2.9 nm. The sensing performance of these EP films was optimized by controlling the thin-film thickness by adjusting the electrochemical parameters. 

Thus, due to the bright yellow-green fluorescence and crosslinked porous texture, the films showed excellent sensory properties with the possibility of their repeated use in the processing of various NACs, namely, TNP, DNP, NP, TNT, NT, and NB. Thus, due to the bright yellow-green fluorescence and porous texture, the films showed excellent sensory properties with the possibility of their repeated use in the processing of various NACs, namely, TNP, DNP, NP, TNT, NT, and NB. For sensing TNP, the LOD of the **polyPhTPECz** EP films was 3.8 nM, and the K_SV_ was calculated to be 1.63 × 10^5^ M^−1^ in an aqueous solution. Additionally, 73% of the fluorescence emission of the film was quenched within 180 s when exposed to TNP vapor. The fluorescence-quenching mechanism for TNP was a combination of electron transfer and resonance energy transfer.

**Scheme 6:**
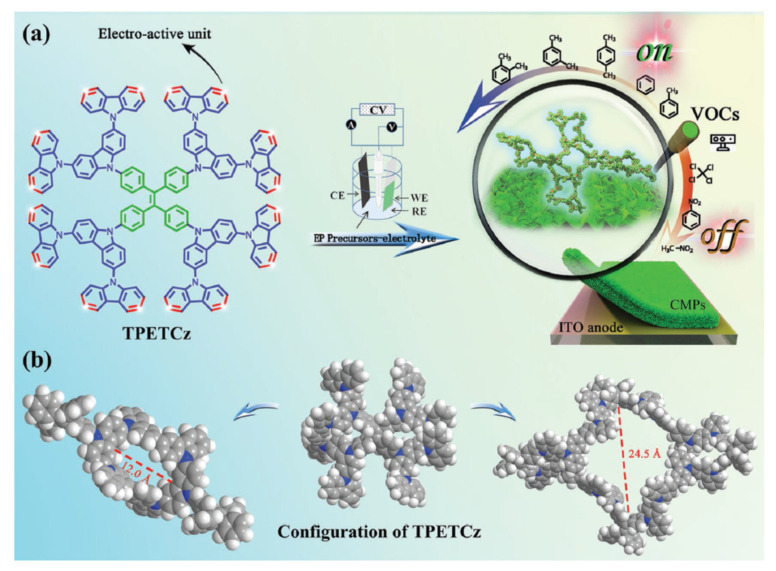
(**a**) Formation of EP film through electropolymerization; (**b**) optimized structures of **TPETCz** and the diameters of their pores. Reproduced with permission from reference [57]. Copyright © Wiley 2020.

Similarly, a porous polymeric EP film with a high specific surface area for analyte diffusion and pore diameters of 1.2 and 2.45 nm in two possible configurations was constructed and studied based on a new dendrimer characterized by its central tetraphenylethylene “core” with an aggregation-induced emission effect and its highly electroactive “branches” (Scheme 6) [57]. Films have been shown to exhibit an on–off fluorescence response to 18 types of volatile organic compounds (VOCs). Significantly, a 2D sensing array was established through **CMP** films and their spin-coated monomer films, and 18 types of VOC vapors were precisely distinguished by linear discriminant analysis.

Currently, conjugated mesoporous polymers/oligomers with pore sizes of 2 to 50 nm are attracting increasing attention as sensors due to their high surface area and porosity, low density, high stability, tunable pores and skeletons, and ease of functionalization, which leads to increased quantum yields and energy transfer efficiency for the ultra-sensitive detection of explosives [58]. 

**Figure 38:**
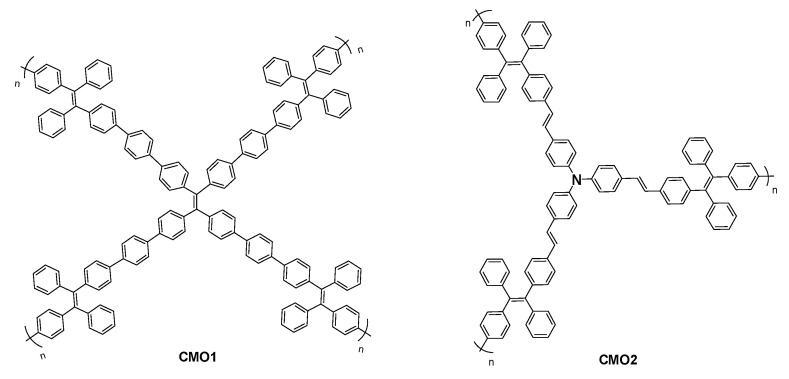
Structures of AIEE-active **CMO1** and **CMO2**.

**Figure 39:**
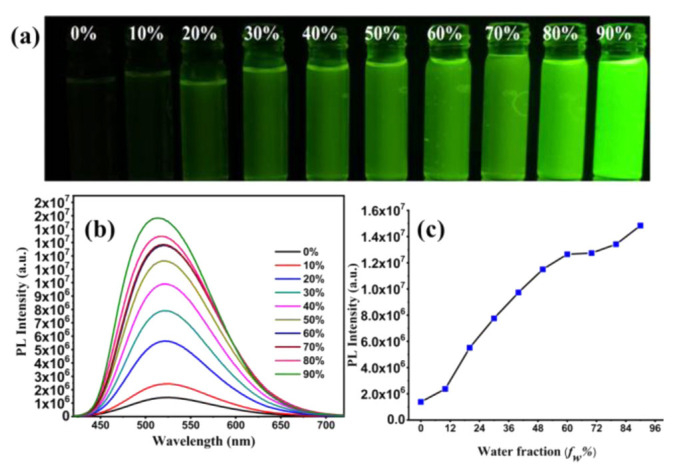
(**a**) Photos of CMO1 in mixtures of THF–water solution under UV light. (**b**) PL spectra of **CMO1** in THF–water mixtures; (**c**) changes in PL intensity of **CMO1** with variations in water fraction. Reproduced with permission from reference [59]. Copyright © ACS Publications 2020.

Thus, AIEE-active mesoporous conjugated oligomers **CMO1** and **CMO2** have been developed (Figure 38) [59]. The integration of typical AIE building blocks, such as tetraphenylethene and triphenylamine (TPA), into **CMO** scaffolds endowed them with AIEE characteristics. Namely, **CMOs** emit weakly in solution, while enhanced and strong emission is observed in the solid state. A 90% aggregated solution formed in a THF/water mixture showed the highest quantum yield for the oligomers and was used to detect TNP (Figure 39). The best results were shown by the **CMO1** oligomer, with K_SV_ of 2.6 × 10^6^ M^−1^ for TNP and 2.3 × 10^3^ M^−1^ for TNT.

**Figure 40:**
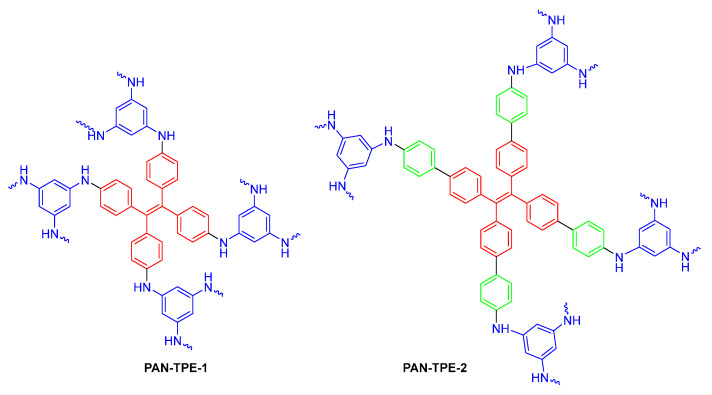
Structures of AIE-active fluorescent polymers.

Pesticide detection with fluorescent porous organic polymers is a new technology developed in 2018 [60]. Thus, two hyper-crosslinked fluorescent porous polymers, **PAN-TPE-1** and **PAN-TPE-2**, were synthesized via the polycondensation reaction of two AIE-active aromatic tetraaldehydes with pores ranging from micropores (1.4–1.9 nm) to mesopores (3.8–26.2 nm) (Figure 40) [61]. The obtained porous polymer sensors showed high fluorescence sensitivity to trace amounts of pesticides in the aquatic environment, as well as excellent discrimination of imidacloprid (IDP) from other pesticides with a value of K_SV_ of 53,745 M^−1^ and a LOD of 28 ppb for **PAN-TPE-1**, which is far lower than the WHO-allowed residue limit in common fruits and vegetables.

#### 3.2.3. AIE-Active Hyperbranched Conjugated Polymers 

**Scheme 7:**
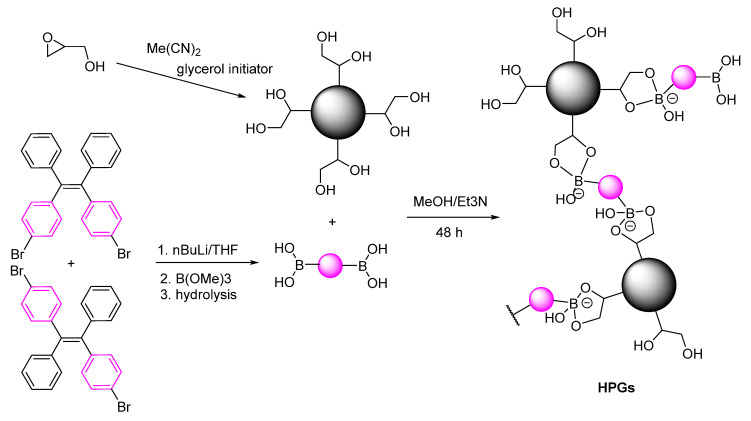
Synthesis of nanosized AIE-active **HPG-TPE** aggregates that exhibit high sensitivity for the detection of nitroaromatic explosives.

AIE-active hyperbranched conjugated polymers are mainly used for the high-sensitivity detection of TNP in aqueous media. This is due not only to the fact that polymer nanoaggregates in an aqueous system have a high fluorescent quantum efficiency due to AIE characteristics but also to the fact that a loose packing of spherical structures forms a large number of intermolecular holes that can bind a larger number of TNP molecules, which leads to higher detection sensitivity [62]. For example, N. Kalva et al. used aggregation-induced emission (AIE)-active hyperbranched polyglycidols (**HPGs**) to design a solid-state sensor for detecting nitroaromatic explosives. The tetraphenylethylene moieties were conjugated to the periphery of **HPGs** in a single step using dynamic boronate ester crosslinkers (Scheme 7) [63]. According to dynamic light scattering and transmission electron microscopic analyses, the self-assembled nanosized aggregates were stable in a THF/H_2_O mixture. The fluorescence of the aggregates was quenched dramatically by different NACs, including nitromethane, 4-nitrophenol, 4-nitrotoluene, 2,6-dinitrophenol, and 2,4,6-trinitrophenol. The nanoaggregates showed remarkable sensitivity to TNP, with a Stern–Volmer constant (K_sv_) of 2.27 × 10^4^ M^−1^ and a LOD of 40 ppb.

**Scheme 8:**
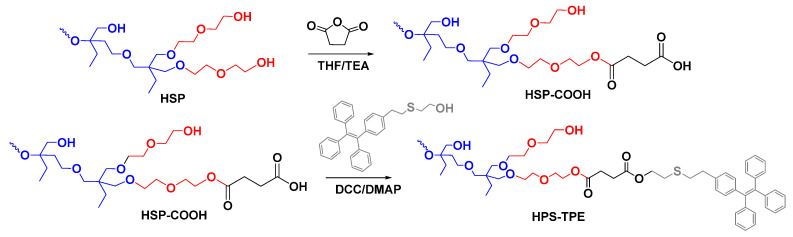
The synthesis of **HPS-TPE** via esterification reaction from **TPE-OH** and **HSP-COOH** produced by carboxylation of **HSP**.

**Scheme 9:**
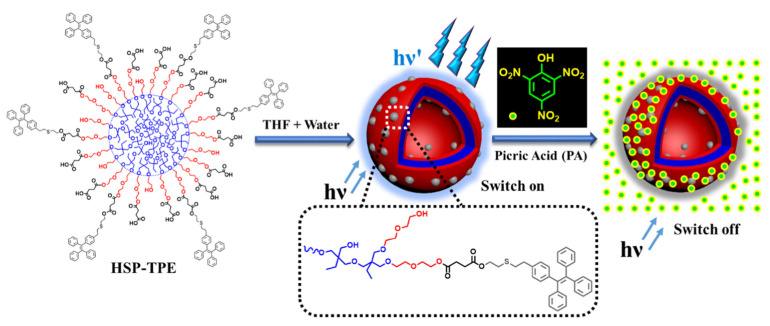
Diagram of copolymer **HSP-TPE** self-assembly and sensing behavior aggregating toward TNF. In **HPS-TPE**, the blue, red, and gray colors represent the hydrophobic core, hydrophilic arms, and the probe, respectively. Reproduced with permission from reference [62]. Copyright © Elsevier 2020.

F. Nabeel et al. reported a hyperbranched **HPS-TPE** polymer prepared through the esterification reaction of poly(3-ethyl-3-oxetanemethanol)-star-poly(ethylene oxide) (**HSP-COOH**) with **TPE**-containing thioethanol (i.e., 2-((4-(1,2,2 triphenylvinyl)phenethyl)thio)ethanol (**TPE-OH**) with high yields (Scheme 8) [62]. In an aqueous solution, the copolymer self-assembles to form aggregates. The aggregates display maximum photoluminescence (PL) in a THF/water (3:7 *v*/*v*) mixture. The introduction of **TPE** units into the polymer made it a highly sensitive AIE probe for picric acid with a detection limit as low as 20 ppb and a quenching constant of 2.4 × 10^4^ M^−1^ (Scheme 9).

**Scheme 10:**
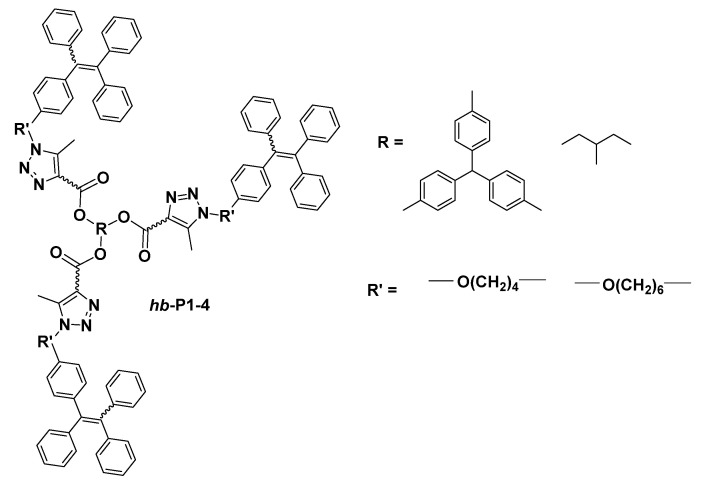
Synthesis of ***hb*-P1–4** by click polymerizations of tributynoates **1** and diazides **2**.

In 2020, four hyperbranched poly(methyltriazolylcarboxylate)s (***hb*-P1–4**) with high molecular weights (Mw up to 2.4 × 10^4^) and regioregularities (up to 83.9%) were obtained via a click polymerization reaction of alkyne monomers with tetraphenylethene-containing diazides (Scheme 10) [64]. The obtained ***hb*-P1–4** were characterized by good solubility, film-forming ability, and thermal stability. Their nanoaggregates (*f*_W_ = 90% in a mixture of THF/water) were studied as chemosensors for the detection of TNF in an aqueous solution. In particular, the Stern–Volmer plots of ***hb*-P1–4** were almost linearly correlated with the TNP concentration below 20 µg mL^−1^, with K_SV_ of 14,290, 14,300, 24,670, and 11,900 M^−1^, respectively.

### 3.3. Donor–Acceptor Architecture of Conjugated Polymers

For conjugated polymer sensors, the introduction of electron-donor groups is thought to enhance the interaction between electron-rich polymers and electron-deficient explosives, resulting in higher sensitivity in both the gas phase and solution, so most of them are based on donor-only structures [65,66,67]. Various conjugated polymers have been extensively studied as thin-film sensor materials due to their extremely high sensitivity, which can be explained based on the molecular wire effect [68]. Owing to the PET fluorescence-quenching process, the main disadvantage of **CP**-based sensors is the problem of selectivity in a complex environment containing various electron acceptors. For example, discrimination between TNT and PA is still a challenge because both of them are extremely strong electron acceptors. In practical applications, selectivity is critical to successful detection, so a lot of effort has been put into this area.

Thus, Wang et al. synthesized the donor–acceptor polymer **P14** and two donor-only polymers **P15** and **P16** to study the dependence of sensitivity and selectivity in the detection of explosives on the structure of the polymer [69]. It was expected that the donor–acceptor structure of the polymer would first lead to a significant intrachain charge transfer from donor groups (diethylamine phenyl) to acceptor groups (2,1,3-benzothiadiazole, BT) and to the appearance of a dipole moment. Second, the LUMO would also mainly localize on the BT units, as occurs in the case of many D-A polymers [70]. 

**Figure 41:**
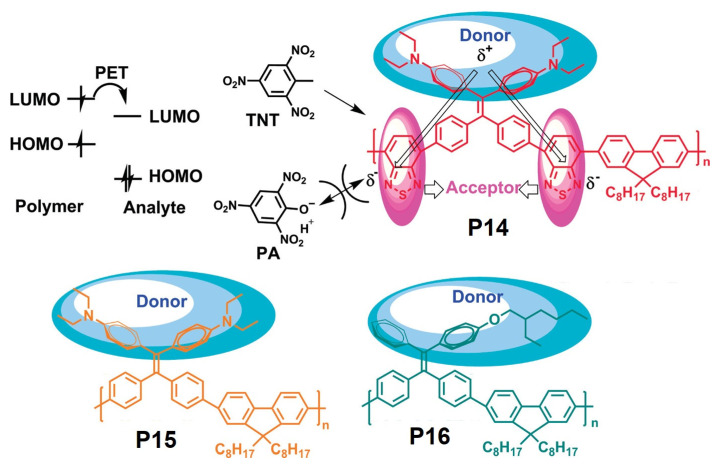
**P14**–**P16** electron transfer fluorescence quenching process and structures. Reproduced with permission from reference [69]. Copyright © ACS Publications 2011.

According to the authors, because PA can easily form a negatively charged anion in an aqueous solution, an electrostatic repulsion interaction between the BT unit and PA will block the efficient electron transfer from the LUMO of **P14** to PA, as shown in Figure 41, which will not happen in the case of TNT. Therefore, P14 can selectively detect TNT instead of PA in an aqueous solution. On the other hand, the reference polymers **P15** and **P16** with only donor groups should behave like most **CP** sensors with higher selectivity toward PA. Although the emission of polymers in the dissolved state was very weak, centrifuged films, due to the AIE activity of the polymers, showed bright emission and were investigated as sensors for detecting a mixture of picric acid and TNT. The **P14** sensor film selectively detected TNT instead of TNP in an aqueous solution with a K_SV_ of 1.2 × 10^5^ M^−1^. The **P16** sensor film selectively detected TNP with a K_SV_ of 2.8 × 10^4^ M ^−1^ and a LOD of 2 ppb. In addition, it was also shown that the emission of **P14** and **P16** films can be effectively quenched by TNT and DNT vapors. These quenching effects of both TNT and TNP on **P14** and **P16** films were found to be reversible.

**Figure 42:**
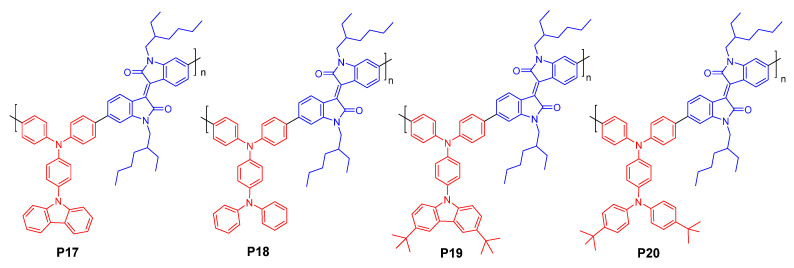
Structures of donor–acceptor conjugated polymers **P17–P20**.

In another study, four D-A-type conjugated copolymers, **P17–20**, based on isoindigo as an acceptor unit with four different triphenylamine (TPA) donor derivatives were prepared (Figure 42) [71]. Quenching the emission of the **P17** sensor upon the addition of picric acid gave rise to a new emission peak, whereas this was not observed when quenching with TNT. The appearance of this new emission peak means that **P17** not only detects TNT and TNP but also selectively detects TNP. The authors suggested that, on the one hand, the addition of TNP results in the protonation of the TPA group, which leads to the quenching of the emission of the sensor; on the other hand, it is likely that a complex with the polymer is formed, which generates a new emission peak and chemical shifts.

**Figure 43:**
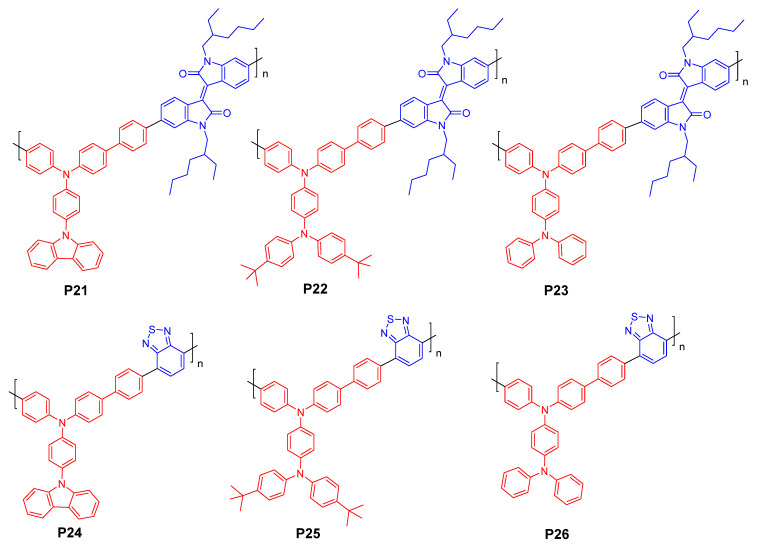
Structures of donor–acceptor conjugated polymers **P21–P26**.

Similar research was published in 2021. Thus, six donor–acceptor conjugated polymers were developed based on triarylamine derivatives as donor units with an isoindigo derivative or benzothiadiazole as acceptor units (Figure 43) [72]. These polymers are characterized by good solubility, film-forming properties in common organic solvents, and high luminescence quantum efficiency (up to 42.9%). In addition to showing electrochromic and photoelectric sensor properties, the films also proved to be highly sensitive probes for the detection of 2,4,6-trinitrophenol (K_SV_ = 45,790 M^−1^) and can meet the requirements of multifunctional materials.

### 3.4. Polymer Sensors Based on Cluster Luminogens 

In some cases, polymers are constructed from nonconjugated monomeric structures by using C-C, C-O, or C-N bonds in polymers to form aggregates or solid forms, which exhibit strong photoluminescence due to so-called “clusterization-triggered emission” [73]. Based on the theory of through-space conjugation (TSC), arising from n–σ*, n–π*, and π–π* orbital overlaps, as well as hydrogen bonding and other weak interactions, the effect of clusterization-triggered emission (CTE effect) can be applied for various applications, for example, in the areas of process monitoring, structural visualization, sensors, and probes [74]. In particular, due to the high content of n- or π-electrons, polymers based on clusteroluminogens are considered promising new candidates for the detection of nitro-explosives.

**Scheme 11:**
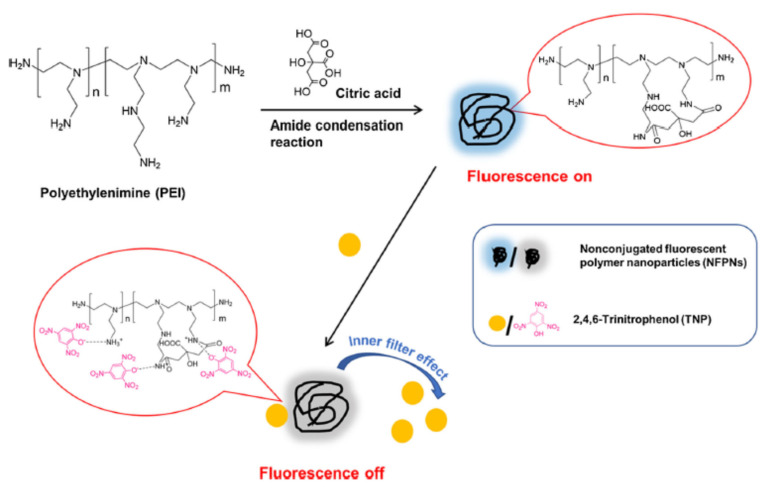
Synthesis of fluorescent polymer nanoparticles (**NFPNs**) from branched polyethylenimine (PEI) and citric acid through an amide condensation reaction and the quenching mechanism of **NFPN** emission by TNP. Reproduced with permission from reference [75]. Copyright © Springer Nature 2020.

For example, very recently, water-soluble fluorescent nonconjugated polymer nanoparticles (NFPNs) containing numerous n-electrons were prepared from branched polyethylenimine (PEI) and citric acid through an amide condensation reaction in the aqueous phase (Scheme 11) [75]. Numerous active amino groups of polyethyleneimine were linked by citric acid and formed the highly crosslinked **NFPNs**. The nanoparticles exhibited stable bright fluorescence, which was quenched dramatically in the presence of trace concentrations of PA. Thus, in an aqueous solution, upon excitation at 360 nm, ca. 40% of the emission of **NFPNs** at 450 nm was quenched upon the addition of PA, while in the presence of other interfering species/quenchers, no noticeable fluorescence changes were observed. The authors ascribed such selectivity to the inner-filter effect (IFE) and the formation of self-assembled non-fluorescent Meisenheimer complexes between PA and **NFPN** side groups on the surface. The calibration plot of log(F_0_/F) vs. PA concentration shows a linear relationship (R^2^ = 0.999) for PA concentrations in the range of 0.5–150 μM. The detection limit for PA was 0.7 μM. The assay was successfully used to determine PA in spiked lake-water samples, and the recoveries were 96.6–102.7%.

**Scheme 12:**
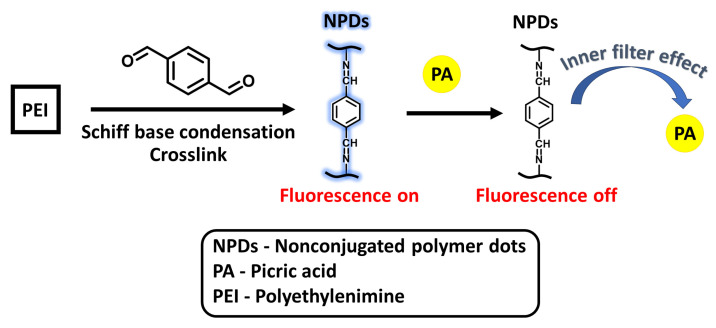
Schematic diagram of the method used to fabricate **NPD** nanoprobes for determining PA using an on–off method.

In a similar manner, nonconjugated polymer dots (**NPDs**) were prepared from PEI and 1,4-phthalaldehyde by means of the formation of Schiff bases in combination with self-assembly (Scheme 12) [76]. These **NPDs** have been successfully used as novel, fast, and sensitive fluorescent probes for PA detection. Thus, **NPD** fluorescence was effectively quenched by PA via the inner-filter effect (IFE) because the absorption spectrum of the analyte and the excitation spectrum of **NPDs** overlapped. The proposed method gave a high degree of precision, good accuracy, and good sensitivity when used to quantitatively analyze PA in water samples. The PA recoveries (101.63–103.15%) and relative standard deviations (2.07–2.16%) were acceptable.

### 3.5. Iptycene-Based Fluorescent Conjugated Polymer Sensors

Iptycene-based conjugated polymer sensors represent a unique combination of two concepts: the concept of the amplified fluorescent conjugated polymer, the so-called “molecular wire” [77], and the concept of interstitial space (free volume) around the molecules of iptycenes, giving them the ability to form supramolecular architectures with high porosity [78]. 

Common polymers, such as polyethylene and polypropylene, are electrical insulators, as they do not have free charge carriers in their backbones. However, *trans*-polyacetylene showed electrical conductivity, which gave rise to conjugated polymers (**CP**s) as semiconductive materials with promising properties from an applied point of view [79]. **CP**s include a wide range of structural classes: *trans*-polyacetylenes, poly(*p*-phenylene)s, poly(*p*-phenylene-vinylene)s, poly(phenylene-ethynylene)s (PPE), poly(*p*-phenylene-sulfide)s, polypyrroles, polythiophenes, poly(3,4-ethylenedioxythiophene)s, polyanilines, and polyfluorenes [79], some of which are fluorescent, which determines their use as sensors as well [80].

Such a possibility was demonstrated for the first time by Zhou and Swager in 1995 using **CP** PPE cyclophanic receptor systems and paraquat as a fluorescence quencher, and the “molecular wire” model was proposed [81,82].

**Figure 44:**
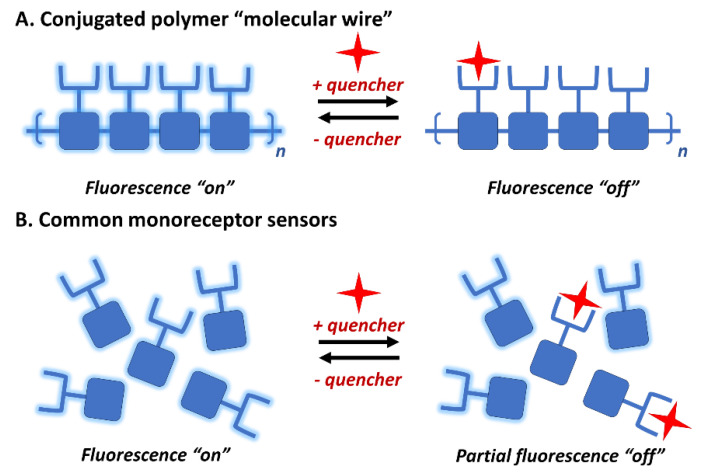
Difference between “molecular wire” polyreceptor system (**A**) and common monoreceptor one (**B**).

In this model, as the authors suggested, receptor units are “wired in series” throughout the fluorescent conjugated polymer, forming a polyreceptor system, which operates on the principle of “*unus pro omnibus, omnes pro uno*” (“one for all, all for one”), making the whole polymer behave as a single molecule. Upon excitation, facile energy migration across the polymer backbone occurs, sampling multiple receptor sites and inducing emission. However, this fluorescence would be significantly quenched if the exciton encounters even a single receptor site occupied by a quencher (Figure 44A). Such a situation, in which a few receptor sites occupied by quencher molecules lead to the almost complete quenching of the whole polymer, is called the amplification of the signal, and such systems were named amplifying fluorescent polymers (AFPs). Classical monoreceptor sensors, typical for isolated small fluorophore molecules, do not have signal amplification like this; thus, the degree of their fluorescence quenching is proportional to the number of receptor sites occupied by the quencher (Figure 44B). Therefore, a higher concentration of the quencher is required for a monoreceptor system to obtain a degree of fluorescence quenching equal to that of the AFP polyreceptor system.

Nevertheless, as one might guess, “molecular wire” AFP systems have their own limitations related to molecular weight (M_n_). Indeed, it was shown that the degree of signal amplification increased at low M_n_ as the diffusion length of the excitation outnumbers the length of the molecule, and then it becomes insensitive at M_n_ above ~65,000–100,000, where the size of the polymer exceeds the diffusion length of the excitation [80,81,82]. Given that the latter is determined by the excited-state lifetime and the excitation’s diffusion rate, to gain the maximum signal amplification degree, a combination of long-lived excitation states, excitations with high mobilities, high-efficiency energy transfer, and high molecular weight is required. For example, it was shown that in molecular structures in which energy transfer is hindered, like in meta-conjugated substituted PPE, the ability to amplify fluorescence quenching is decreased [82]. 

Another similar case is the lowered sensitivity of AFPs in solution due to the isolation of polymeric chains from each other. It causes excitons to walk in a one-dimensional way, which is ineffective because excitons revisit the same receptor sites several times as a result. Also, in the solution state, the competitive solvation of AFPs and quencher molecules and their rates of diffusion should be taken into account as additional limitations. In addition, carrying out fluorescence-quenching experiments in the solution state requires meticulous solution preparation and the usage of toxic solvents [80].

As a one-time solution to the above-mentioned problems, the usage of AFP sensors in the form of films instead of solutions was proposed. This allows the sample preparation stage to be eliminated, as the film is a ready-to-use sensor device, which is particularly convenient for operators in field conditions. Also, there was hope that the limitations imposed by the diffusion rate, which is linked to the process of sensor and quencher solvation, would be reduced. But the most important advantage of films over solutions lies in their two- or three-dimensional structure. It eliminates the one-sided walking behavior of excitons, which is obligatory in the solution state, and allows hopping from one chain to nearby ones. Such multidimensionality of potential routes decreases the possibility of the exciton revisiting the same receptor sites and increases the likelihood of it meeting a site occupied by a quencher, which further advances the amplification of the signal [78,83]. 

For example, in poly(*p*-phenylene ethynylene) thin films prepared by Langmuir–Blodgett deposition, an increase in energy transfer efficiency accompanying an increase in the number of layers up to 16 layers was observed. The phenomenological model used for the evaluation of energy transfer efficiency revealed a high rate of energy transport between different layers (>6 × 10^11^ s^−1^) and a diffusion length exceeding 100 Å in the Z-direction [5]. 

But just as solution-state AFP sensor usage has its own problems and limitations, the same can be said for the solid-state one: (1) The planar geometry of the above-mentioned polymers favors π-stacking interactions and results in aggregation. (2) Such interactions, in turn, create local energy minima on the potential energy surface of the film. With the addition of intra- and interchain exciton migration, a large amount of the initial excitation energy is funneled to aggregation sites in AFP thin films. Also, these low-energy trap sites restrict the extent of exciton migration [80]. (3) Aggregation is accompanied by the well-known phenomenon called “aggregation-caused quenching” (ACQ) [84], expressed as a lower fluorescence intensity compared to the same polymer in the non-aggregated state. (4) Weak emission, in turn, means the low sensitivity of the sensor polymer, as higher concentrations of the quencher are required to give a reliable response. 

Despite the advantages of such systems for enhanced fluorescence-quenching efficiency, the initial **CP** sensors lacked the visualization of recognition events and selectivity.

**Figure 45:**
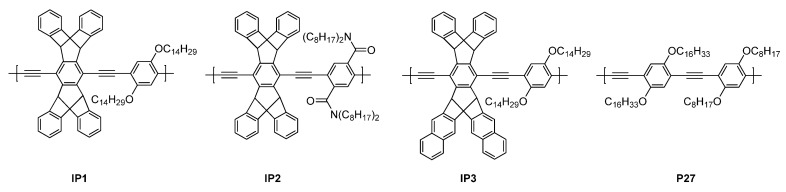
Structures of Yang and Swager’s original iptycene-based conjugated polymers.

To overcome the listed disadvantages and pitfalls of classical **CP**-AFP sensors, especially the films, and to increase their selectivity, the introduction of sterically bulky pendants into the structure was suggested. Triptycene-containing polymers have been known since 1968 [85]; thus, in 1998, Yang and Swager designed new **CP**-AFP sensors but based on a pentiptycene moiety (**IP1–3**) (Figure 45) [65,66].

**Figure 46:**
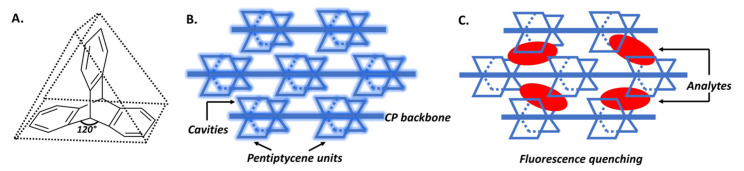
Internal free volume and dihedral angles of iptycene (**A**); concept of “sponge”-like porous structure of iptycene-based polymers (**B**) and their fluorescence quenching after contact with analytes (**C**).

Iptycene turned out to be the most attractive candidate, as it has a unique three-dimensional structure and properties. The dihedral angles between the aromatic rings of its bicyclo [2.2.2]octatriene bridgehead system are equal to 120 °C, resulting in a rigid three-bladed geometry (Figure 46A), which hampers π-stacking and other intra- and intermolecular interactions and makes molecules resistant to aggregation [86]. This results in (1) higher fluorescence intensity and quantum yields, as ACQ does not happen (with iptycene-based **IP1–3** having greater fluorescence quantum yields compared to **P27** in the solid state); (2) improved solubility in organic solvents (as **IP1** is ~100-fold more soluble than **P27** in chloroform); (3) higher spectroscopy stability and reproducibility; (4) the enhanced stability of the polymer films to thermal actions, solvent treatment, photobleaching, and degradation, since the enthalpic driving force for the reorganization of the polymer chains is smaller when there is no direct contact [66]; (5) increased energy transfer efficiency through polymer layers due to the prevention of energy waste upon exciton migration [80].

Furthermore, such voluminous pendants stack up in the polymer film in such a way that voids are formed, named “internal free volume” (Figure 46A). Consequently, it increases the porosity of the film, turning it into a kind of “sponge” (Figure 46B). The cavities in the polymeric “sponge” serve as molecular-scale channels, which facilitate the penetration of analytes and their adjustment to receptor sites (Figure 46C).

It is also worth mentioning that the clefts and cavities of iptycenes afford a venue and direct the course for supramolecular interactions and additionally enable size-based selectivity in some cases [87].

Also, as for selectivity, iptycenes are electron-rich, and therefore, in the presence of electron-deficient analytes (e.g., nitroaromatic explosives, their precursors, and their derivatives), the fluorescence quenching of iptycene-based **CP**-AFPs occurs due to noncovalent interactions, i.e., photoinduced electron transfer (PET) [66].

Based on the above, first, **CP**-AFP pentiptycene-based sensors **IP1–3** (planar electron-rich polymer **4** was synthesized as a reference compound) were developed by Yang and Swager in 1998, as was mentioned earlier, and their sensory response to electron-acceptor compounds—nitroaromatic ones and quinones—were studied. Structure-activity relationships were investigated to understand what it takes to create effective and sensitive fluorescence “turn-off” sensors for the detection of explosives and some other analytes [65,67].

**Figure 47:**
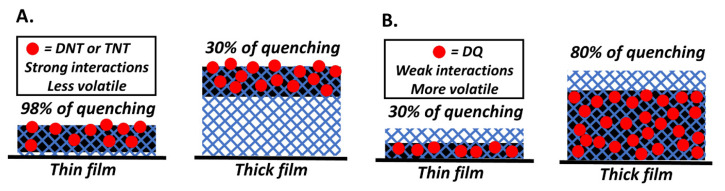
Dependence of quenching efficiency on film thickness and analyte nature (nitroaromatic compounds (**A**) or duroquinone (**B**)).

Fluorescence-quenching experiments in the solid state, specifically in films, showed that quenching depends on the analyte diffusion rate, which, in turn, correlates with sensor–analyte binding interactions, the vapor pressure of the analyte, and the film thickness. For nitroaromatic quenchers, the relationship between the film thickness and quenching efficiency was inversely proportional (Figure 47A); in thin films (25 Å), a greater quenching efficiency was obtained compared to thick (200 Å) ones (70% and 30% at 60 s for TNT, respectively). This happens because of the strong binding interactions between the sensor and nitroaromatic compounds, which slow down the diffusion of the latter through the film. For quinones (namely, duroquinone (DQ)), the opposite was observed because of weaker interactions (Figure 47B) [65,66]. And the dependence of the sensory response on the vapor pressure of the analyte manifests itself in the following: DNT, having a higher vapor pressure than TNT (18:1), caused more pronounced and faster fluorescence quenching (80% vs. 30 % after 10 s in thin films, respectively).

Now, if you think about it, the dependence of the quenching efficiency of **CP**-AFP sensors on the analyte diffusion rate runs somewhat counter to the concept of the “molecular wire”, as the latter should not depend on the analyte diffusion rate so much. If the “molecular wire” model were completely correct, then the occupation of receptor sites on the surface by the quencher should be enough for rapid, substantial fluorescence quenching. Indeed, in one of the later studies, a time-resolved quartz crystal microbalance with in situ fluorescence measurements confirmed that the fluorescence-quenching efficiency is mainly related to analyte diffusion, not exciton diffusion, as in real-time sensing, the diffusion of the vapor of the analyte–quencher into the film is driven by the swelling kinetics of the latter [88]. Thus, according to these findings, optimizing and improving analyte penetration should be one of the priorities when designing future sensors operating in the solid state. However, regardless of the real mechanism behind high quenching efficiency—the “molecular wire” or diffusion-dependent one—iptycene-based **CP**-AFP sensors proved themselves effective and promising for detecting nitro-containing substances, especially their vapors.

By virtue of the initial studies of iptycene-based polymer sensors carried out by Yang and Swager, several ways to improve sensitivity can be distinguished: (1) increasing the electron richness of the sensor, making it more “appealing” to electron-acceptor substances (for example, the electron-withdrawing character of amide substituents in polymer **IP2** leads to its decrease, which results in a reduction in sensor sensitivity to nitroaromatics [66]); (2) tuning the porosity or configuration (size/shape) of 3D cavities; (3) increasing the excited-state lifetime, which expands the range of through-bond exciton migration; (4) tuning sensor–analyte binding interactions (not too strong, not too weak); (5) increasing the fluorescence quantum yield via exciplex formation (due to excited energy delocalization) or the aggregation-induced emission (AIE) phenomenon [89], which also elevates the interchain exciton transport simultaneously.

Since isolated fused polycyclic aromatics have rigid structures and symmetrically forbidden/weakly allowed transitions, instead of “classic” PPE pentiptycene-containing polymers **IP1–3**, the dibenzochrysene-based π-conjugated analog **IP5** was created for the extension of the excited-state lifetime. As was supposed, due to the structural modification made, improved sensitivity toward TNT was observed [90].

The introduction of chiral alkyloxy side chains into PPEs gave polymer **IP6**, which aggregates into a 3D helical grid structure with longer exciton diffusion and conjugation lengths. Films of **IP6** exhibited a rapid and substantial sensory response: exposure to 10 ppb of TNT vapor led to 75% fluorescence quenching in 10 s. Moreover, the quenching efficiency of the **IP6** film depended very little on its thickness. These results indicated a great improvement in sensitivity and performance compared to **IP1–3** films [91].

**Figure 48:**
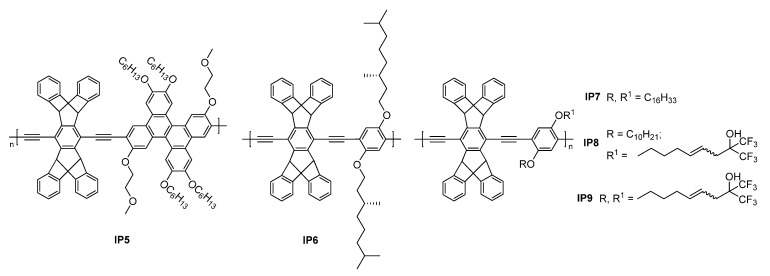
Structures of iptycene-based conjugated polymers (**IP5–IP9**).

Similar to **IP6**, but having pendant hexafluoro-2-propanol (HFIP) groups instead of alkyloxy side chains, derivatives **IP7–8** were synthesized (Figure 48) [92]. It was found that the introduction of strongly hydrogen-bond-donating pendants such as HFIP is more effective in terms of the detection of hydrogen-bond-accepting analytes (e.g., pyridine and 2,4-dichloropyrimidine) compared to weak hydrogen-bonding but strongly electron-deficient ones such as DNT (only 20–28% fluorescence quenching was observed upon exposure to DNT). So, it can be concluded that this modification strategy showed itself to not be successful in the design of sensitive sensory materials for the detection of explosives and their derivatives.

**Figure 49:**
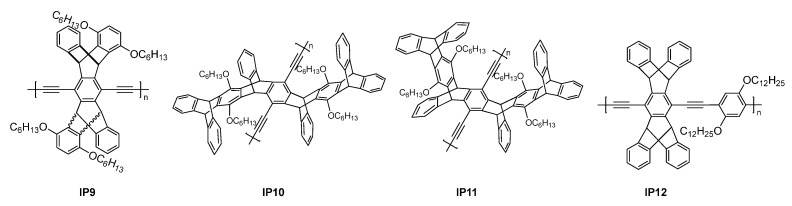
Structures of iptycene-based conjugated polymers **IP9–IP12**.

As was mentioned earlier, tuning the size/shape of 3D cavities, as well as binding interactions, should also be taken into account while designing new sensory materials. Thus, novel all-iptycene-based poly(p-phenylenebutadiynylene)s (PPD) **IP9–11** were synthesized (polymer **IP12**, similar to “classic” PPE polymers **IP1–3**, was obtained as the model compound) (Figure 49) [67]. A crucial point of such a structural modification lies in the fact that every *p*-phenylene unit within the conjugated backbone is built into the iptycene structure as the core unit in such a way that it lies at the bottom of the three-dimensional rigid “nest”. Consequently, direct “face-to-face” interactions between nitroaromatic molecules and these p-phenylene units are restricted, which is fundamentally different from the above-mentioned open-structure iptycene-based polymers **IP1**–**8**.

To evaluate the sensory properties of the obtained PPD polymers **IP9**–**11**, fluorescence-quenching experiments were carried out in the presence of nitroaromatic substances (DNT, TNT, *p*-nitrotoluene, and benzophenone) both in solution (in CHCl_3_) and in the solid state. Interestingly, among the four polymers, **IP10** exhibited the highest sensitivity: the Stern–Volmer static quenching constant values varied within 21–185 M^−1^ (for **IP9** and **IP11**, they varied within 13–166 M^−1^ and 1–64 M^−1^, respectively; for **IP12**, the quenching constant for DNT was equal to 26 M^−1^). For all polymers, the greatest sensory response was obtained in the presence of TNT, which can probably be attributed to the largest number of electron-withdrawing nitro-groups in its structure among all involved in the quenching experiment’s nitroaromatic compounds. Considering that **IP10** and **IP11** have similar electronic properties, the difference in their sensitivity can be attributed to the influence of the stereo-configurations of the iptycene moieties.

However, in the solid state, the opposite behavior was observed: films of PPDs **IP9**–**11** showed a much slower response to the presence of DNT and TNT compared to PPE **IP12**. Moreover, the fluorescence recovery rate after the cessation of contact with the vapor of the quencher was slow for **IP9** and took an extremely long time for **IP10**–**11**, indicating almost irreversible binding between iptycene cores and nitroaromatic molecules, which hinders the exit of the quencher from the cavity of the sensor.

In summary, two conclusions can be drawn: first, different factors play a role in sensing electron-deficient compounds by PPD sensor polymers in solution and in the solid state; second, tuning the quencher’s binding degree should be very fine and weighted, as too strong a retention of the analyte hampers its diffusion, which, in turn, negatively affects the sensor sensitivity. It is also especially important if the aim is to design recoverable sensory materials.

**Figure 50:**
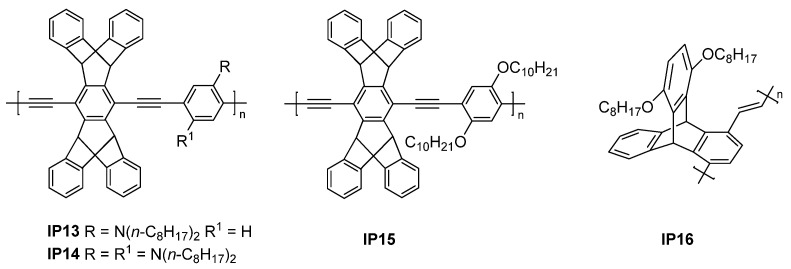
Structures of iptycene-based conjugated polymers **IP13–IP16**.

Another interesting design attempt is the creation of sensory polymers **13**–**14** (Figure 50) with greater photoreduction abilities for the detection of the taggant 2,3-dimethyl-2,3-dinitrobutane (DMNB), which is quite hard to detect due to its reduction potential of approximately –1.7 V and three-dimensional structure, eliminating the possibility of direct π-stacking with the sensor film [93]. The idea was to introduce amines into the structure, whose π-donation would reduce the ground-state oxidation potential. However, it turned out that it also lowers the band gap, simultaneously increasing the excited-state oxidation potential. Only the polymer **IP13** film gave a sensory response to DMDN, and it was very weak—around 4–5%.

It is worth mentioning that triptycene was also used in CP-AFPs as an aggregation-preventing unit, as well as an electron donor for electron-deficient compounds, such as explosives, despite the fact that triptycene is less effective than pentiptycene in the alleviation of ACQ [94].

The classic PPE polymer **IP15** was obtained together with the triptycene-based poly(phenylenevinylene) (PPV) one, **IP16**, both of which exhibited multiphoton-excited fluorescence that was sensitive to the presence of TNT (Figure 50) [95]. To compare their sensory abilities, two-photon- and three-photon-excited-fluorescence-quenching measurements were carried out by microtitration in solution. The pentiptycene-based polymer **IP15** showed a larger degree of two- and three-photon-excited-fluorescence quenching than the triptycene-based (**IP16**) one (quenching factor of ~5 vs. ~1.2 at millimolar concentrations, respectively). The results may indirectly indicate that pentiptycene is a more preferable unit for CP-AFP sensors than the triptycene one.

**Figure 51:**
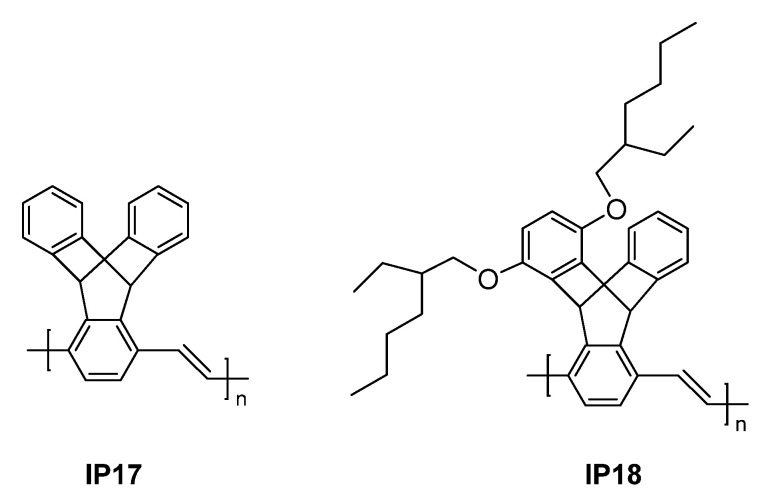
Structures of iptycene-based conjugated polymers **IP17–IP18**.

Another two triptycene-based PPVs—**IP17** and its derivative **IP18**, having O-alkyl-donating substituents—may serve as examples of the influence of structural modifications on sensory abilities (Figure 51) [96]. Electron-donor substituents lead to an increase in the energy of the highest occupied molecular orbital, with a subsequent decrease in the oxidation potential of polymer **IP18**. Also, they add bulkiness to the already-voluminous triptycene moiety, reducing the tendency to aggregate and increasing the overall electron richness of polymer **IP18**. Indeed, more pronounced fluorescence quenching in the solid state in the presence of DNT was observed for polymer **IP18** than for its analog **IP17**—55% vs. 37% after 60 s, respectively. Another advantage of polymer **IP18** is that it can be easily reactivated. The Stern–Volmer quenching constants, measured in solution, were equal to 12.8 M^−1^ and 28.4 M^−1^ for **IP17** and **IP18**, respectively, which is in accordance with the previously mentioned constant values for **IP9–12**.

**Figure 52:**
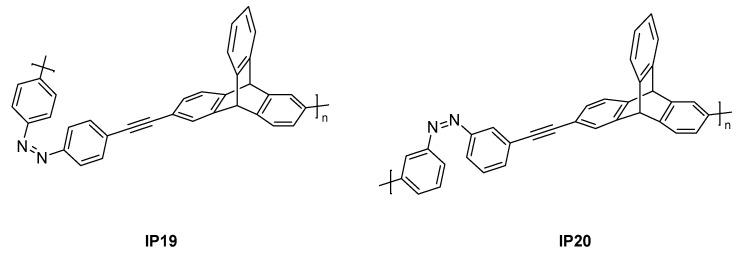
Structures of iptycene-based conjugated polymers **IP19–IP20**.

Other examples of using triptycene moieties for the creation of sensory materials are azobenzene-functionalized polymers **IP19**–**20** for the detection of picric acid (PA) (Figure 52) [97]. Facile interactions (hydrogen bonding, π−π, or dipole–dipole ones) of polymers **IP19**–**20** with PA were suggested due to the electron-rich character of the polymer backbones and the presence of nitrogen-rich azo (-N=N-) linkages in the polymer frameworks. Indeed, fluorescence quenching amounted to 78% for **IP19** and 57% for **IP20**, and the values of Stern–Volmer quenching constants varied within (2.4–5.3) × 10^5^ M^−1^, which are several orders of magnitude higher than those obtained for any of the above-mentioned polymers, indicating the high sensitivity of the obtained polymers to PA. Thus, the applied strategy of structural modification proved to be successful.

**Figure 53:**
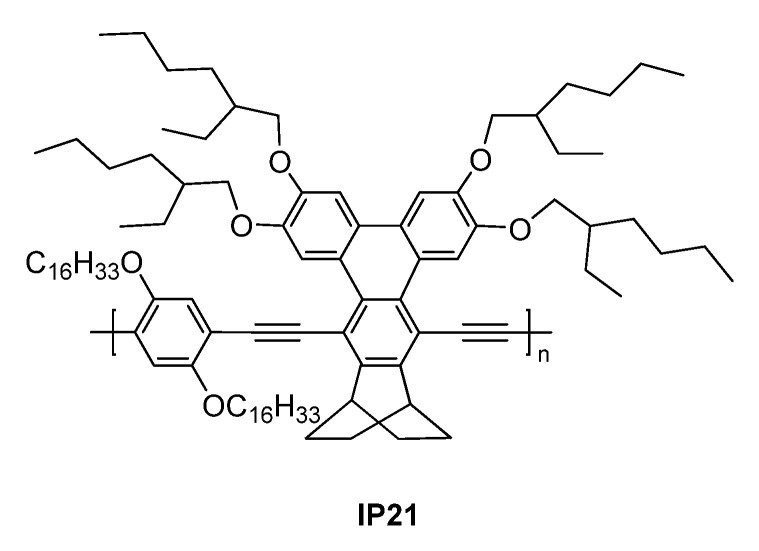
Structure of iptycene-based conjugated polymer **IP21**.

The peculiar compilation of design strategies for polymers **IP5** and **IP18**, namely, the incorporation of fused polycyclic aromatics and O-alkyl-donating substituents, yielded polymer **IP21**, in which the structure’s triphenylene core was integrated (Figure 53) [98]. It allowed an increase in bulkiness and electron-donating propensity at the same time. This, in turn, increased selectivity, since triphenylene has three-fold symmetric sites, which are complementary to the positive charges on TNT and DNT, and sensitivity to nitroaromatic explosives. Polymer **IP21** exhibited improved sensitivity to TNT vapor.

**Figure 54:**
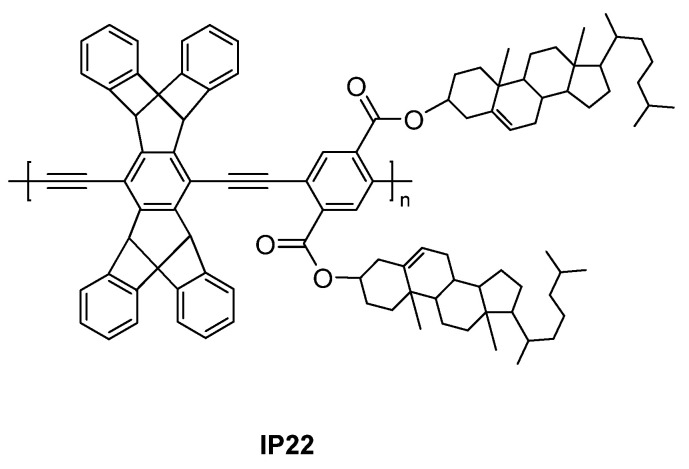
Structure of iptycene-based conjugated polymer **IP22**.

An unusual pentiptycene-based PPE sensor polymer, **IP22**, having cholesteryl ester substituents, was designed (Figure 54) [99]. Bulky pendant cholesteryl ester groups act as an additional measure to prevent aggregation and increase the porosity of the polymer film. In the solid state, fluorescence quenching of 51% after 60 s exposure to DNT vapor was observed for the thin film (2 nm). Undercoating with 7.5–8.5 nm thick APTES and blending with a non-fluorescent polymer added 18.5% (with 20 s exposure) and 18.7% (with 5 min exposure) to the fluorescence intensity decrease, respectively. Thus, polymer **IP22** has shown itself to be relatively efficient for the detection of DNT and capable of improvement.

**Figure 55:**
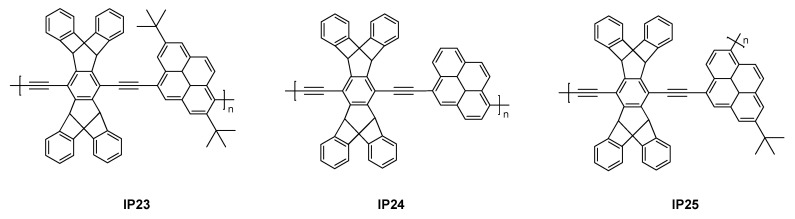
Structures of iptycene-based conjugated polymers **IP23**–**IP25**.

The outstanding photophysical properties of pyrene, for example, its high fluorescence quantum yield combined with long-lived excited states, electron-rich nature, and sensitivity to microenvironmental changes, are well known, which is why pyrene has a long history of being used as a basic and classic unit for the creation of sensitive sensory materials for the detection of explosives [100]. Thus, pentiptycene-based **CP**s **IP23**–**25**, containing pyrene or a *^t^*butyl-substituted derivative in the main chain, were created (Figure 55) [101]. It can be said that the same design strategies were applied in this case as for polymer **IP5**, having dibenzochrysene units, and polymer **IP21**, containing a triphenylene core with bulky substituents. Polymer films of **IP23**–**25** were able to detect TNT in both the vapor and aqueous states, which confirms the effectiveness and feasibility of the chosen design strategies. It should be said that polymer **IP25**, having mono *^t^*butyl-substituted pyrene, showed the best performance, which tells us about the need for balance in bulkiness. Fluorescence quenching of the **IP25** polymer film by TNT vapor was equal to 60.4% and 90% after 10 s and 30 s of exposure, respectively, which is higher than those exhibited by previously mentioned polymer sensors. Also, fluorescence quenching of 69.8% was observed for the **IP25** polymer film within 60 s of immersion in an aqueous solution of TNT (50 M). Moreover, sensor films of polymers **IP23**–**25** are recoverable after usage.

**Figure 56:**
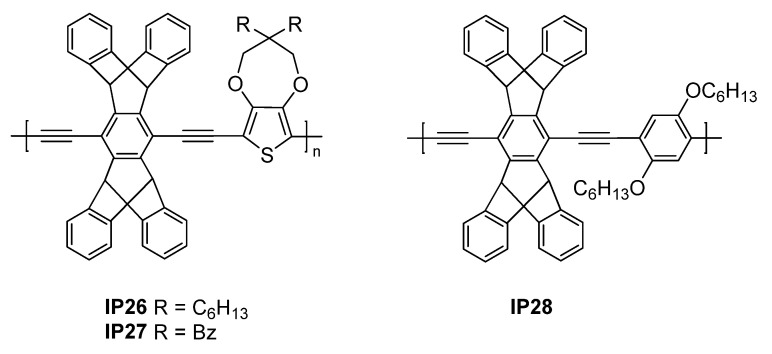
Structures of iptycene-based conjugated polymers **IP26–IP28**.

Series of poly(aryleneethynylene)s (PAEs) based on electron-rich dialkylated 3,4-propylenedioxythiophenes (ProDOT) and pentiptycene polymers **IP26**–**28** were synthesized as sensors for the detection of the explosive TNT (“classic” PPE polymer **IP28** was obtained as a model compound) (Figure 56) [102]. The fluorescence quantum yields of **IP26**–**27** are higher than those for wholly ProDOT-based PAE polymers due to the pentiptycene unit’s incorporation but lower than the yields of fully PAE polymers and PPE polymer IP**28**, thanks to the presence of sulfur, inducing the heavy-atom effect. However, the sensitivity of **IP26–27** to the presence of TNT in CHCl_3_ turned out to be on par with that of polymer **IP28** despite the lower quantum yields of the former. It is worth mentioning that a later organic composite combining poly 3-hexylthiophene, Cu (II) tetraphenylporphyrin, and **IP27** was produced and able to detect vapors of such explosives as 1,3,5-trinitro-1,3,5-triazacyclohexane (RDX), TNT, and dinitrobenzene by measuring the saturation current and conductance as the key parameters representing molecular the recognition event [103].

**Figure 57:**
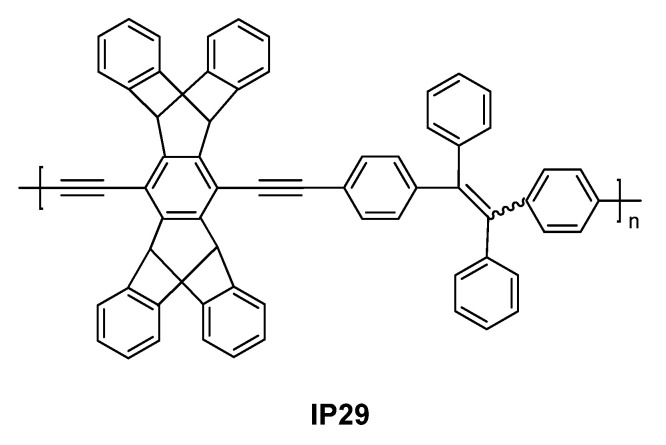
Structure of iptycene-based conjugated polymer **IP29**.

Since tetraphenylethene (TPE)-based polymers are aggregation-induced emission (AIE)-active, a versatile and sensitive pentiptycene-based TPE-containing sensor polymer, **IP29**, capable of detecting explosives (PA and dinitrotoluene) both in solution and in air was created (Figure 57) [104]. In aqueous organic media (in a H_2_O–THF mixture (9:1 (vol. %)) to be more precise), it forms highly emissive and sensitive nanoaggregates that detect nitroaromatic explosives at ppb levels: fluorescence quenching was equal to 24% with a PA concentration of 4.36 × 10^−8^ M and reached over 98% with a PA concentration of 4.71 × 10^−6^ M. In the solid state, 70% of the fluorescence of the thin film (4 nm) was quenched by DNT vapor after 30 s, whereas for the thick film (80 nm), quenching was only 37%; thus, thickness-dependent behavior of quenching was observed, which agrees with the previous data for model polymers **IP1–3**.

### 3.6. Pyrene-Containing Excimer-Based Polymer Sensors

It was mentioned earlier that pyrene has unique photophysical properties, which is why it has found widespread use in the design of sensory materials. The excimer emission of pyrene is of particular interest, as it is very sensitive to microenvironmental conditions such as temperature, pressure, or pH [105]; thus, sensors in which the formation of pyrene excimers takes place are highly desirable.

Many pyrene-based sensors have been created, including small-molecule ones [100,106] and polymer films [105], but the latter have advantages, as they are cheap, user-friendly, convenient to use on-site, reusable, usually organic-solvent-free (which is more eco-friendly), and, ideally, easy to prepare. There are several types of pyrene-containing polymer materials based on the type of pyrene introduction into the polymer structure: thin film with covalently bonded pyrene pendants [105] and physically encapsulated pyrene molecules in non-porous or porous materials [107,108]. Each type has its own advantages and disadvantages.

The production of thin polymer films with covalently attached pyrene or its derivative pendants usually prevents leakage and decreases excimer emission decay; however, it can result in weak excimer emission. Also, the covalent bonding of pyrene molecules is time-consuming, tedious, and costly and usually yields low amounts of the target product. On the other hand, they are preferable for the detection of analytes in solutions. Poor excimer emission can also be observed in the case of the physical encapsulation of pyrene molecules in non-porous films. Moreover, the dense polymer matrix hinders the diffusion of the analyte, reducing the sensitivity of the material. To reduce this effect, very thin films can be prepared, but they have low fluorescence intensity, which makes results obtained with their usage unreliable and unrepeatable. To enhance excimer formation and facilitate analyte diffusion through the polymer, a non-porous type of polymer can be designed, but the leakage issue must be kept in mind. In addition, it should be mentioned that the physical entrapment of the polymer in either non-porous or porous materials can lead to inhomogeneity and reduce the stability of the material, lowering its performance and the reproducibility of the results.

Despite the above-listed issues, design strategies have been polished over the years to keep only the positive properties of different materials while eliminating the negative ones, which allowed the production of effective sensory materials suitable for particular types of analytes/application fields. Several interesting examples are discussed below.

**Figure 58:**
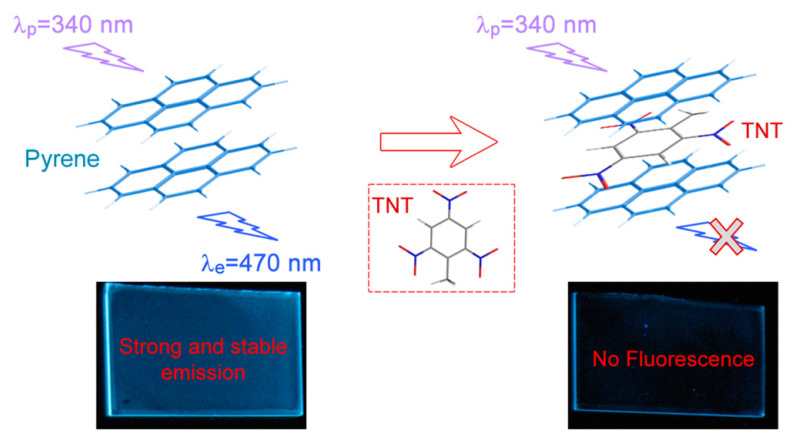
Schematic illustration of fluorescence-quenching-based TNT sensing with pyrene molecules. Reproduced with permission from reference [109]. Copyright © ACS Publications 2014.

Beyazkilic et al. prepared sensory mesoporous thin films via a facile template-free sol–gel method based on organically modified silica (ormosil) with entrapped pyrene molecules for the detection of nitroaromatic compounds, including explosive ones (Figure 58) [109]. The advantages of this material are its cheapness, its simplicity, and the lack of a need for any chemical modifications of pyrene. Also, porosity facilitates analyte penetration, which is especially useful for the detection of analytes in the form of vapor. Moreover, pyrene excimer emission was observed. Its intensity was higher in a porous film than in its non-porous counterpart, which is expected, as it is well known that pores promote excimer formation [110]. Furthermore, in porous films, it was stable for at least 2 months, whereas in non-porous films, pyrene excimer emission decayed within 2 h. The solid-state fluorescence-quenching efficiency of pyrene excimer emission by DNT, TNT, and nitrobenzene was equal to 39.2, 31.5, and 16.2% after 30 s of exposure, respectively. These results are lower than those obtained for iptycene-based polymer films; however, this quenching can be observed by the naked eye under a UV lamp, which is convenient for no-instrument, fast, on-site analyte detection. Other analytes (toluene, ammonium nitrate, benzene, benzoic acid, sodium hydroxide, 3,4-dihydroxybenzoic acid, methanol) caused no sensory response, which testifies in favor of film selectivity. Also, it is worth mentioning that the obtained sensory films can be recovered and used again at least five times by just immersing them in water.

**Figure 59:**
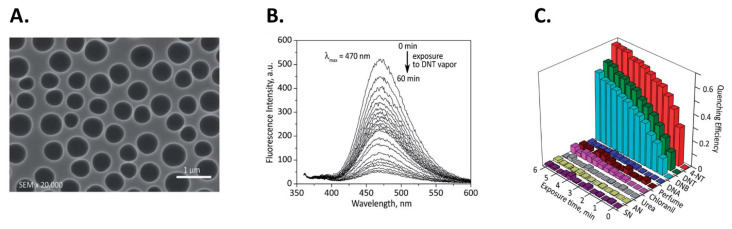
Nanoporous **Py–PS** film (SEM image) with an average pore size of ~400 nm (**A**); fluorescence intensity upon exposure to equilibrium DNT vapor (time-dependent) (**B**); fluorescence-quenching efficiency at the Py excimer peak upon exposure of the nanoporous **Py–PS** film (3D) to saturated vapors of various analytes (**C**). Reproduced with permission from reference [111]. Copyright © Royal Society of Chemistry 2014.

Another intrinsic example of pyrene-based porous sensory material is the self-assembled 3D nanoporous pyrene–polystyrene (**Py-PS**) film, which was prepared via the simple single dip coating of PS and Py solutions (Figure 59A) [111]. The authors managed to fine-tune its sensitivity by fine-tuning the morphology and by optimizing the degree of pyrene doping into the polymer matrix (via varying the pyrene–styrene molar ratios), which affects the electronic properties. The best-sensing film quenched the pyrene excimer emission intensity by 60% and 90% after 6 min and 30 min of exposure to saturated DNT vapor (Figure 59B); thus, the produced film is pretty sensitive. Also, the selectivity of the film was additionally proven by the absence of fluorescence quenching in the presence of other analytes, such as 3,5-dinitroaniline, perfume, chloranil, urea, ammonium nitrate, and sodium nitrite (Figure 59C).

**Figure 60:**
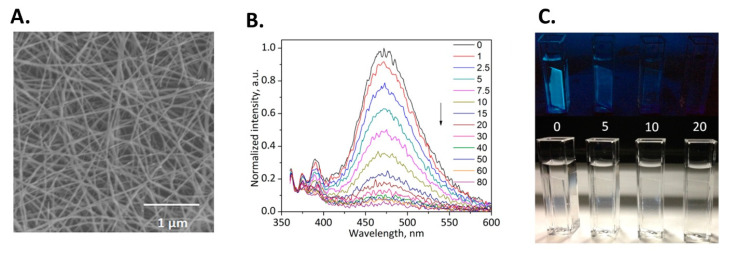
Nanofibrous **Py–PES** (SEM image) (**A**); fluorescence quenching (concentration-dependent) of Py–PES nanofiber film during addition of different concentrations (μM) of TNT in deionized water (**B**); photos of Py–PES fluorescent nanofiber films under UV light (254 nm, upper panel) and visible light (bottom panel) in the presence of various TNT concentrations (0, 5, 10, and 20 μM) (**C**). Reproduced with permission from reference [112]. Copyright © ACS Publications 2015.

As seen in the above-mentioned examples of iptycene- and pyrene-based porous films, this type of material is more suitable for the detection of analytes in the vapor state. However, for detection in an aqueous matrix, it was concluded by Sun et al. that sensors in the form of nanofibers are preferable, as nanofibers are cost-effective, having high porosity and a large surface area of approximately 1–2 orders of magnitude higher than those of continuous thin films, enhancing their sensitivity and response time, and it is also possible to produce fibers with low polymer concentrations and, in turn, small diameters, which are favorable for fluorescence sensing [112]. Thus, pyrene–polyethersulfone (**Py–PES**) nanofibers were prepared under optimized conditions via the electrospinning technique using mixed solvents (Figure 60A). **Py–PES** nanofibers proved themselves to be highly sensitive, as pronounced excimer emission quenching was observed in the presence of explosives, such as TNT, DNT, and 1,3,5-trinitroperhydro-1,3,5-triazine (RDX), in aqueous solutions (Figure 60B,C). The Stern–Volmer quenching constants were calculated to be 1.80 × 105 M^−1^, 7.52 × 104 M^−1^, and 3.26 × 104 M^−1^, respectively, with limits of detection of 23, 160, 400, and 980 nM, respectively. In addition, other analytes, namely, ethanol, acetonitrile, benzonitrile, chlorobenzene, and toluene, caused almost no changes in fluorescence intensity. Moreover, the immersion of the **Py–PES** nanofiber film in water for 48 h also did not affect the fluorescence intensity, which indicates the absence of leakage. As in the case of the previously mentioned pyrene-based materials, the **Py–PES** film is reusable.

**Figure 61:**
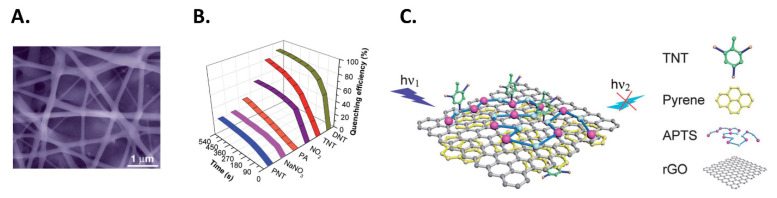
**PVP/pyrene/APTS/rGO** nanonets (SEM image) (**A**); quenching efficiencies of **PVP/pyrene/APTS/rGO** nanonets upon exposure to DNT, TNT, PA, NaNO_3_, and PNT subsaturated vapors and NO_2_ (50 ppb) at room temperature with respect to time (**B**); fluorescence-quenching mechanistic pathway of **PVP/pyrene/APTS/rGO** nanonets toward TNT (**C**). Reproduced with permission from reference [113]. Copyright © Royal Society of Chemistry 2014.

Another example of sensory pyrene-based nanofiber-like material was reported by Guo et al. [113]. Novel flexible polyvinylpyrrolidone/pyrene/3-aminopropyltriethoxysilane/reduced graphene oxide (**PVP/pyrene/APTS/rGO**) nanonets (Figure 61A) were produced by using a one-step electrospinning method for the detection of explosives. Almost every component synergistically plays a role in enhancing the sensitivity of the final product. The need for pyrene usage in designing sensors for the detection of electron-acceptor substances, such as explosives (e.g., TNT, DNT), is clear. APTS was chosen, as aromatic electron-deficient compounds can be absorbed by electron-rich amino groups of organic amines via the formation of charge-transfer complexes between amino groups and the aromatic rings. rGO serves as a charge transport bridge between pyrene and TNT to facilitate the fluorescence quenching of the former. Also, rGO can interact with TNT through co-facial π–π stacking, inducing efficient long-range energy migration (Figure 61C). As so many steps were taken to improve the performance of the sensory material, the high sensitivity of **PVP/pyrene/APTS/rGO** nanonets was not surprising, since this was expected. For example, the fluorescence-quenching (via excimer emission quenching) efficiency was 81% and 89% after 540 s of exposure to TNT and DNT vapors, respectively (Figure 61B). A sensory response to NO_2_, NaNO_3_, PA, and *para*-nitro toluene was also observed: the quenching efficiency was up to 53%, 18%, 34%, and 18% within 540 s of exposure, which speaks in favor of nanonets’ sensitivity to TNT and DNT.

**Figure 62:**
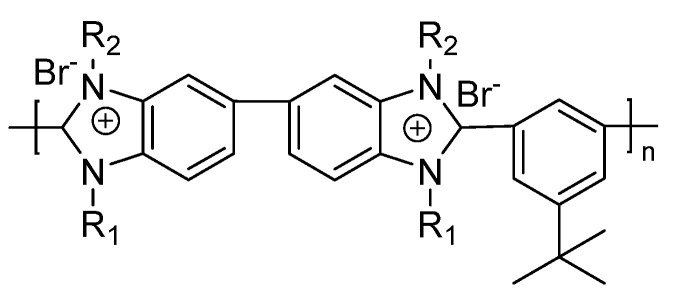
Structure of polymeric ionic liquids (PILs) made from polybenzimidazole (PBI-BuI).

Unusual sensory fluorescent film-forming polymeric ionic liquids (PILs) containing pyrene or anthracene moieties were made via the solution polycondensation of 3,3′-diaminobenzidine and 5-*tert*-butyl isophthalic acid in polyphosphoric acid (Figure 62) [114]. The produced films showed intense excimer emission and moderate solution-state and high solid-state sensitivity (for ~12 µm thin film) to common nitroaromatic compounds. In DMSO, the fluorescence intensity was reduced by 70%, 60%, and 46% in the presence of PA, TNT, and nitrobenzene, respectively, in the case of the pyrene-based material (**[DPyDBzPBI-BuI][Br]**), and for the anthracene-based one (**[DAnDBzPBI-BuI][Br]**), fluorescence quenching was a little bit lower—55%, 53%, and 44%, respectively—which can be attributed to the lower electron richness and the tendency toward excimer formation of the latter. The Stern–Volmer quenching constants varied within (1.78–4.15) × 10^4^ M^−1^ for **[DPyDBzPBI-BuI][Br]** and (1.59–2.03) × 10^4^ M^−1^ for **[DAnDBzPBI-BuI][Br]**. However, the excimer emission of the **[DPyDBzPBI-BuI][Br]** film was reduced by 87%, 76.8%, and 71.6% quenching after 500 s exposure to nitrobenzene, TNT, and PA, respectively, and 80.3%, 73.5%, and 67.5% of the fluorescence intensity of the **[DAnDBzPBI-BuI][Br]** film was quenched by nitrobenzene, TNT, and PA, respectively. It should be noted that for thick films (~40 µm), a decrease in sensitivity was observed: fluorescence was quenched only by 11% and 30% after 50 s and 300 s of exposure to nitrobenzene, respectively, but this phenomenon is not unusual. Both films possess selectivity, as they showed no sensory response to benzoquinone, toluene, THF, or NaNO_3_. An additional benefit is that the films can be renewed by washing with methanol after usage.

**Figure 63:**
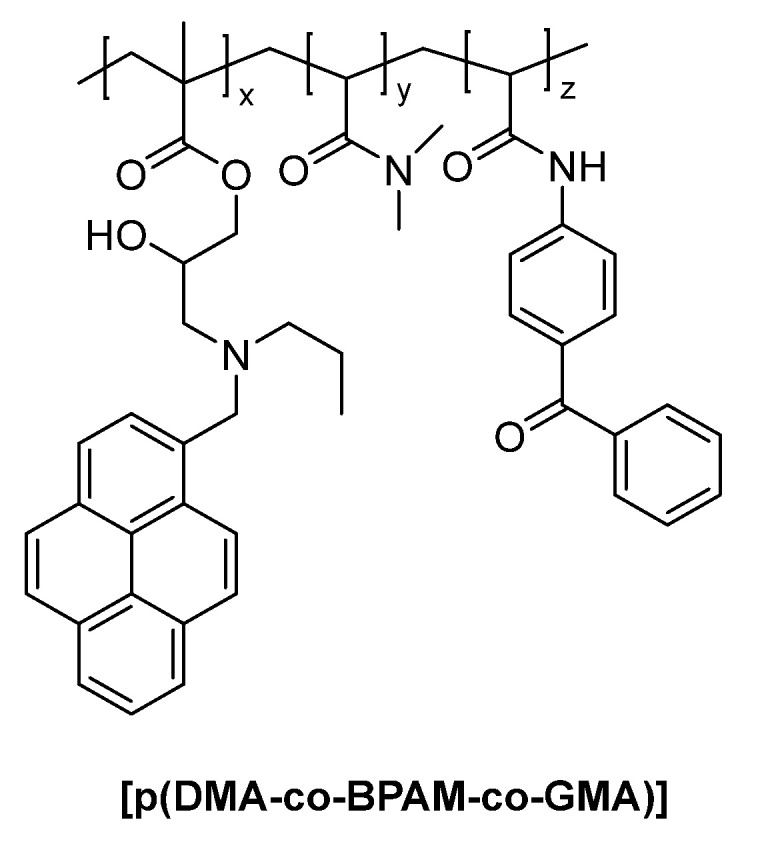
Structure of **[*p*(DMA-co-BPAM-co-GMA)]**.

Though “classic” thin polymeric films with covalently attached fluorophores have their own disadvantages, as was mentioned above, this type of material can still be useful if designed correctly and can find its own application field. One such example is Gupta and Lee’s thin polymeric film with pyrene pendants synthesized via the free radical copolymerization of dimethylacrylamide (DMA), benzophenone acrylamide (BPAM), and glycidyl methacrylate (GMA) with a feed ratio of 95:1:4 (Figure 63) [33]. The final product—**[p(DMA-co-BPAM-co-GMA)]**—was initially designed for the sensitive and selective detection of PA. Both pyrene units and their attachment to tertiary amine groups provided a fluorescence “turn-off” sensory response to the presence of PA, and its separation from other analytes (DNTs, nitrophenols, dimethylnitrobenzene, 4-nitrotoluene, various anions and cations, etc.) by electrostatic interactions between the positively charged polymer and picrate in water and crosslinkable benzophenone moieties requires immobilizing the film onto quartz slides. The proximity of pyrene moieties throughout the polymer backbone resulted in molecular aggregation and π−π stacking, which amplified fluorescence quenching by PA via an exciton-hopping process via intermolecular electronic coupling, facilitating long-range exciton diffusion. The limit of detection for PA was as small as 11.4 atto-grams (10^−14^ M), and the Stern–Volmer quenching constant was determined to be 7.75 × 10^4^ in water. Also, the recovery of fluorescence was possible for the polymer by washing it with just water, which is pretty convenient.

### 3.7. Optical Sensors Based on Porous Fluorescent Polymers: The Molecular Imprinting Technique

Molecularly imprinted polymers (**MIPs**) have received widespread attention due to the creation of selective artificial recognition cavities [115]. As probes, molecularly imprinted polymers are synthesized by the copolymerization of functional monomers and crosslinking agents in the presence of matrix molecules, which can be either explosives or their non-explosive structural analogs. By removing such matrices from the polymer substrate, cavities are created, resulting in the selective and accurate detection of explosives [116]. Despite the variety of different synthesis methods, there are currently no commercially available **MIP** sensors. Due to the change in the conformation of the binding sites during the drying of the polymer, **MIP** sensors are described in the literature only for detecting explosives in solution [116].

The infusion of carbon dots (**CDs**) with **MIPs** has been widely applied because of the high sensitivity of **CDs** and the high selectivity of **MIPs** [117]. A facile strategy to immobilize amino-carbon dots (**CDs**) onto mesoporous structured imprinted polymers for the highly sensitive and selective detection of TNT was developed, and a mesoporous structured sensor, **M-MIPs@CDs**, was obtained [118]. The selective sensing of TNT was guaranteed by the molecular imprinting technique and improved by two methods. One involved using mesoporous silica as an imprinting matrix, and the other entailed using fluorescent amino-**CDs** directly as a “functional monomer”. The thus-obtained sensory material exhibited excellent selectivity and sensitivity toward TNT, with a detection limit of 17 nM. The recycling process was sustainable for 10 cycles without an obvious efficiency decrease. The feasibility of the developed method with real samples was successfully evaluated through the analysis of TNT in soil and water samples, with satisfactory recoveries of 88.6–95.7%.

**Figure 64:**
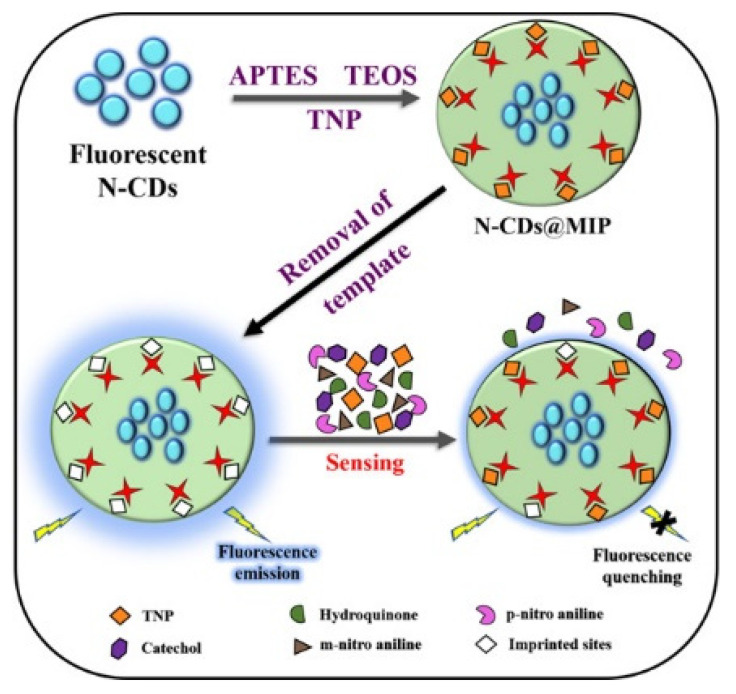
Schematic diagram of the fluorometric sensor **N-CDs@MIP** and quenching mechanism of the **N-CDs@MIP** emission by TNP. Reproduced with permission from reference [119]. Copyright © ACS Publications 2022.

In another example, a fluorometric sensor based on nitrogen-passivated carbon dots infused with a molecularly imprinted polymer (**N-CDs@MIP**) *via* a reverse microemulsion technique using 3-aminopropyltriethoxysilane (APTES) as a functional monomer, tetraethoxysilane (TEOS) as a crosslinker, and PA as a template was published (Figure 64) [119]. The removal of template molecules leads to the formation of a molecularly imprinted layer, and the **N-CDs@MIP** fluorescence response was quenched by PA. The developed fluorescence probe shows a fine linear range from 0.5 to 2.5 nM with a detection limit of 0.15 nM. The synthesized fluorescent probe was used to analyze PA in regular tap- and lake-water samples.

The influence of the nature/amount of the crosslinker, as well as the template–monomer ratio, on the fluorescence intensity and sensitivity of two fluorescent polymers, 7-hydroxy-4-methylcoumarin acrylate (HMC) and 2,6-bis-acrylamidopyridine (BAP), was studied by Apodaca and co-authors [120]. Ethylene glycol dimethacrylate (EGDMA), trimethylolpropane trimethacrylate (TRIM), and divinyl benzene (DV55) were used as crosslinkers. The target analytes were 1,4-dinitrotoluene (DNT), trinitrotoluene (TNT), and *N*-methyl-3-cyano-4-methoxy-2-pyridone (ricinine, neutral alkaloid). It was established that **HMC-MIP** displayed a fluorescence-quenching effect, whereas the binding of the template to **BAP-MIP** yielded an enhancement of the fluorescent signal. In addition, the **MIP** formulation incorporating 20 mmol EGDMA yields an **MIP** with an obvious change in the fluorescence response for the detection of chemical warfare agents. Such a formulation exhibits the potential of detecting TNT in the µM concentration range. The resulting imprinted polymers were assumed to possess structural rigidity brought about by the interaction of BAP and HMC with the target analyte, allowing the **MIP** to respond via a change in the fluorescence signal intensity upon binding to ricinine (an analog of ricin), DNT, TNT, and other analytes.

**Figure 65:**
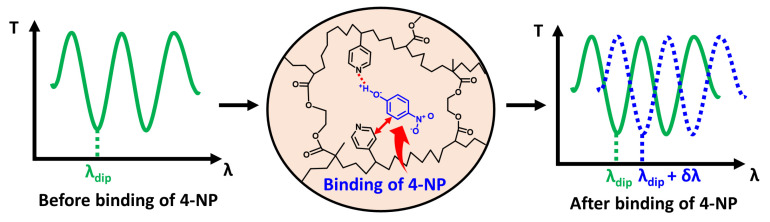
Schematic representation of working principle of the proposed sensor **MIP-NP**.

The imprinted-polymer-functionalized optical sensor **MIP-NP** based on ethylene glycol dimethacrylate (EGDMA) for hypersensitive and selective water pollutants in aqueous media, with a dynamic detection range of 10^−12^–10^−4^ M measured using a concatenated microfiber interferometer technique, was developed (Figure 65) [121]. In the present detection system, **COM** was used for the generation and retention of stable interference patterns, while the MIP was used as a receiving agent for 4-nitrophenol (4-NP). The sensitivity of the proposed sensor was measured at 6.14 × 10 ^11^ nm/M with a LOD of 1.628 fM.

### 3.8. Optical Sensors Based on Fluorescent Polymers Containing Macrocycles

Conjugated macrocycle polymers, as a combination of extended π-conjugated polymers with macrocycles, are an emerging class of organic porous materials [122]. Conjugated macrocycle polymers synthesized by integrating macrocycles such as crown ethers, porphyrins, phthalocyanines, pillararenes, calixarene, and others demonstrate many flexible modular design possibilities for specific structures and functions, particularly for the detection of nitro-containing analytes.

**Figure 66:**
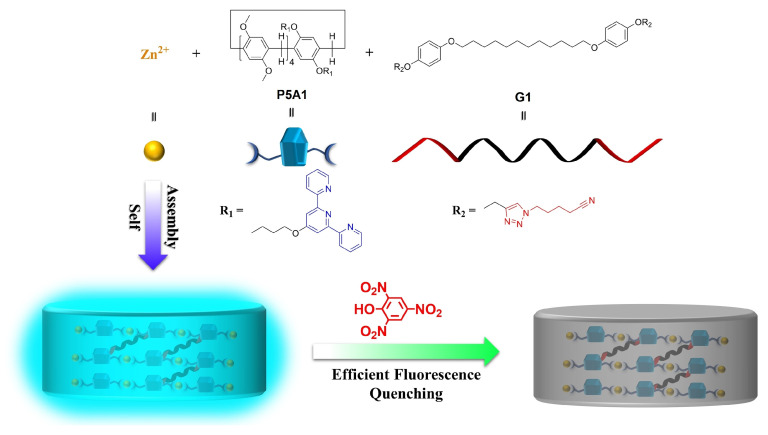
Schematic diagram of supramolecular organo-gel fabrication and picric acid sensing. Reproduced with permission from reference [123]. Copyright © Wiley 2021.

A “three-components” supramolecular assembly composed of 2,2′:6′,2″-terpyridine attached pillar[5]arene **P5A1**, a guest molecule containing 1,2,3-triazole-bearing CN-terminated alkyl chain **G**, and Zn^2+^ was fabricated (Figure 66) [123]. A supramolecular organo-gel was formed with a concentration of components amounting to 1 M, which acted as a sensor probe for nitrobenzene-based explosives. In particular, the limit of detection for picric acid was determined to be 1.66 × 10 ^−4^ M, with a fluorescent-quenching efficiency of 83.7%.

**Figure 67:**
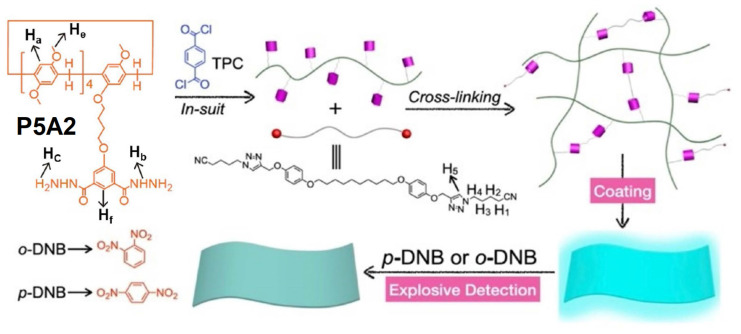
Pillar[5]arene-containing polymer structure and a neutral guest **G** bearing two cyano sites and two triazole sites. Schematic representation of crosslinked supramolecular polymer network formation with the property of explosive detection. Reproduced with permission from reference [124]. Copyright © Springer Nature 2022.

A linear supramolecular polymer obtained via the substitution reaction of the hydrazide groups on pillar[5]arene derivative **P5A2** with terephthaloylchloride (TPC) was used to construct a sensor for detecting nitro-explosives. Then, a supramolecular polymer network was successfully constructed via the introduction of a neutral guest **G** through pillar[5]arene-based host–guest interactions (Figure 67) [124]. The fluorescence intensity of the resulting film decreased dramatically upon the addition of nitro-compound explosives o-DNB and p-DNB. The limits of detection (LODs) of the probe for o-DNB and p-DNB were determined to be 7.90 × 10^−7^ M and 4.40 × 10^−7^ M, respectively.

**Figure 68:**
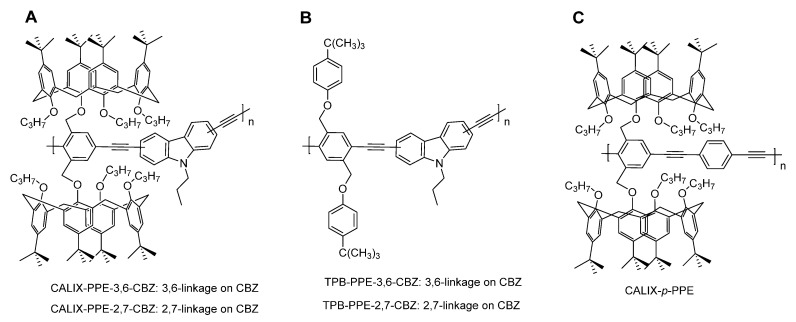
(**A**) Molecular structures of poly({2,5-bis-[5,11,17,23-tetrakis(1,1-dimethylethyl)-25-(oxymethyl)-26,27,28-tripropyloxycalix[4]arene]-p-phenylene}ethyny-lene-alt-3,6 or 2,7-carbazolyleneethynylene) (**CALIX-PPE-CBZs**); (**B**) poly[2,5-bis(p-tert-butylphenoxymethyl)-p-phenylene]ethynylene-alt-3,6 or 2,7-carbazolyleneethynylene (**TBP-PPE-CBZs**); (**C**) poly({2,5-bis-[5,11,17,23-tetrakis(1,1-dimethylethyl)-25-(oxymethyl)-26,27,28-tripropyloxycalix[4]arene]-p-phenylene}ethynylene-p-phenylene ethynylene) (**CALIX-p-PPE**).

The concept behind the assembly of calix[4]arenes with a carbazole-containing conjugated polymer (**CALIXPPE-CBZs**) [125] was used to construct a new conjugated calix[4]arene-containing polymer, **CALIX-p-PPE**, which had a *p*-phenylene ethynylene-type main chain [126]. Five sensory materials were studied for the detection of non-volatile nitroaliphatic liquid nitromethane (NM) and the explosive taggant 2,3-dimethyl-2,3-dinitrobutane (DMNB) in the vapor phase: two **CALIXPPE-CBZs** (Figure 68A), which differed in the structural arrangement of the diethynyl substitution (3,6- or 2,7-) pattern on the carbazole units; two non-calixarene-based polymers, **TBP-PPECBZs** (Figure 68B), with the same polymer backbone; and a **CALIX-*p*-PPE** (Figure 68C) [127].

Experimental studies and theoretical calculations confirmed the hypothesis that the calixarene hosts were the unique differentiating elements responsible for the overall enhanced sensing in the solid state. The most sensitive and selective polymers in the detection of NB and DMNB were carbazole-containing calix[4]arene-based **CP**s (**CALIX-PPE-CBZs**), which was ascribed to cooperativity effects developed between the calix[4]arene hosts and the phenylene ethynylene–carbazolylene main chains. In particular, the CALIX-PPE-3,6-CBZ film was the best sensory material for NM and DMNB and reached around 60% quenching efficiency after only 10 s of exposure.

Recently, the same authors published studies of past research on the best calix[4]arene-containing phenylene-alt-ethynylene-carbazolylene polymers (**Calix-PPE-CBZs**) for the detection of nitroaromatic explosive compounds in solution and in the vapor phase. Both fluorophores exhibit high sensitivity and selectivity for NAC detection. The quenching efficiencies in solution, assessed through static Stern–Volmer constants K_SV_, follow the order picric acid (PA) >> 2,4,6-trinitrotoluene (TNT) > 2,4-dinitrotoluene > (2,4-DNT) > nitrobenzene (NB) [128].

**Figure 69:**
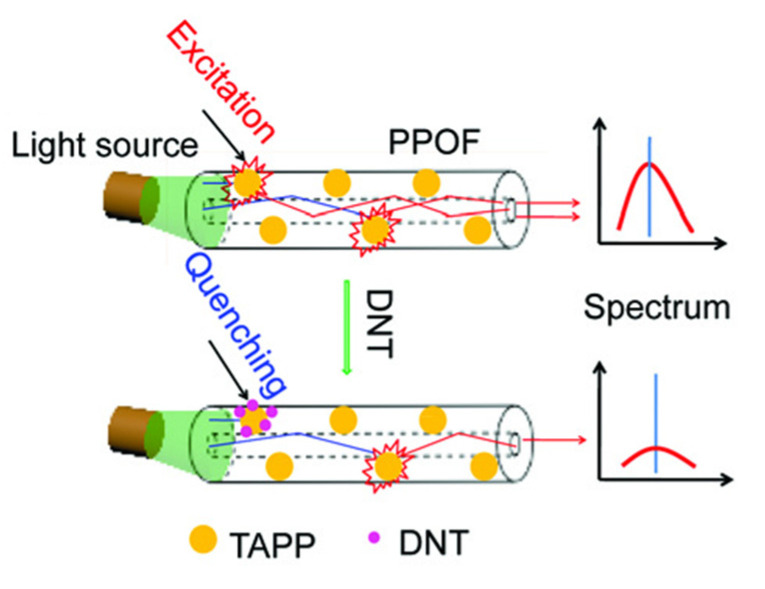
Schematic representation of the **PPOF** sensor’s working principle for monitoring DNT vapor. Reproduced with permission from reference [129]. Copyright © Royal Society of Chemistry 2020.

A novel kind of flexible 5,10,15,20-tetrakis(4-aminophenyl)porphyrin (TAPP)-doped polymer optical fiber (**PPOF**) for the rapid and accurate detection of trace 2,4-dinitrotoluene (DNT) in vapor was reported by Gu and co-authors [129]. When the **PPOF** was pumped with green laser light, the TAPP molecules were excited, and subsequently, the emitted fluorescence was transmitted along the **PPOF** and recorded by a spectrometer (Figure 69). It was experimentally demonstrated that the limit of detection and response time of the proposed **PPOF** could reach around 120 ppb and 3 min, with a fluorescence quenching efficiency of 80.5%.

### 3.9. Paper-Based Sensors

In view of sustainability and green concepts, paper-based electronics [130] and sensorics seem attractive for practical applications. Below, the most representative examples of paper-supported chemosensors for the detection of explosives are described.

**Figure 70:**
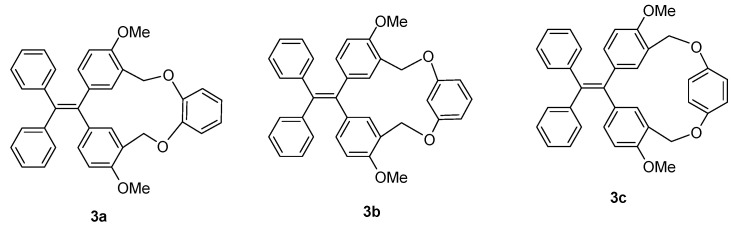
TPE-based chemosensors **3a–c**.

**Figure 71:**
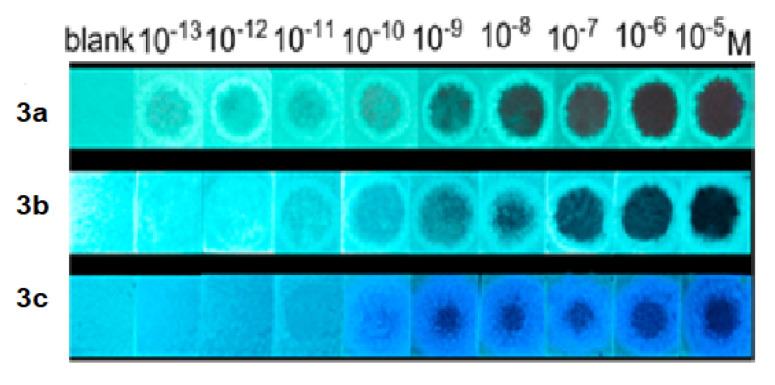
Chemosensor **3a–c**-impregnated paper strips in presence of different concentrations of TNT under UV light (365 nm). Reproduced with permission from reference [131]. Copyright © ACS Publications 2014.

Most paper-supported chemosensors are based on tri- or tetraphenylene fluorophores. Thus, Feng and co-authors [131] reported tetraphenyle **3a–c**-impregnated AIE sensory materials for the detection of methyl-substituted nitro-explosives (Figure 70). These materials exhibited a “Turn-off” response to TNT at a concentration as low as 1.0 × 10^−13^ M with a LOD of 0.2^−4^ ppb. The best response, with a LOD of 0.45 pg × cm^−3^, was observed for chemosensor **3a** (Figure 71), while chemosensors **3b–c** exhibited lower sensitivity.

**Figure 72:**
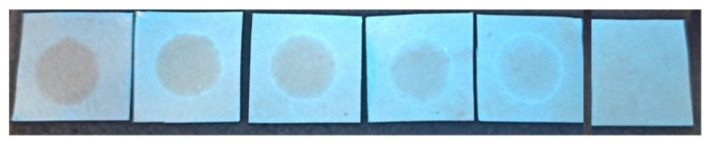
Chemosensor **2**-impregnated paper strips in presence of different concentrations of PA (up to 10 μM) under UV light (365 nm). Reproduced with permission from reference [63]. Copyright © Elsevier 2021.

In Section 3.2.3, hyperbranched polyglycidols, as highly fluorescent AIE fluorophores/chemosensors, are reported (Scheme 7) [63]. In aqueous solutions, these chemosensors formed nanoaggregates and exhibited a strong response to PA, with a Ksv of 2.27 × 10^4^ M^−1^ and a LOD of 40 ppb. When impregnated on the surfaces of paper strips, these chemosensors exhibited the intensive quenching of blue fluorescence in response to the presence of PA (0 to 10 μM) (Figure 72).

**Figure 73:**
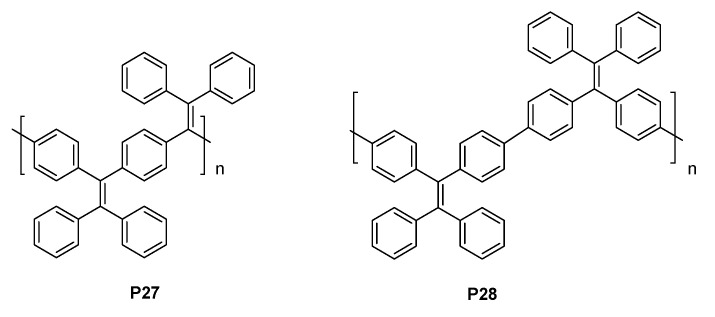
Structures of poly(triphenyl)- and poly(tetraplehyl) ethene polymers **P27–28**.

**Figure 74:**
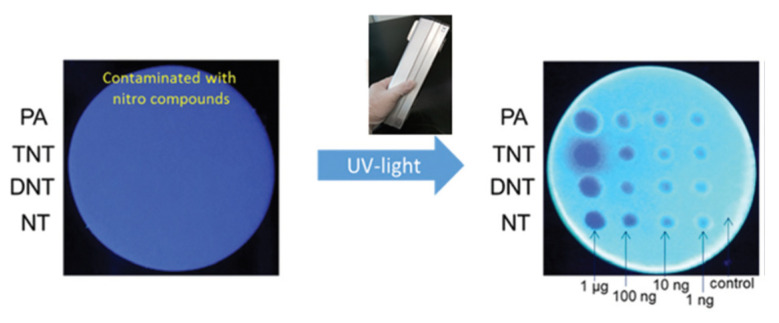
Detection limit of the paper sensor **P27** for nitro-compounds (PA, TNT, DNT, and NT). The images of the paper sensor are under UV light radiation (365 nm). Reproduced with permission from reference [132]. Copyright © Royal Society of Chemistry 2016.

Zhou and co-authors reported poly(triphenyl)- and poly(tetraplehyl) ethene polymers **P27**–**28** (Figure 73) as sensory materials for the “turn-off” detection of nitro-explosives [132]. As the first step, polymer films were fabricated from chemosensor **P27** by using drop coating (film thickness of 5.51 ± 0.36 µm), spin coating (film thickness of 2.00 ± 0.24 µm), and electrospinning (film thickness of 1.61 ± 0.06 µm) and from the electrospun chemosensor **P27** (film thickness 1.54 ± 0.16 µm). Due to the larger contact surface (S_BET_ value of 120 m^2^ g^−1^ for the electrospun film of chemosensor **P27** vs. S_BET_ = 6 m^2^ g^−1^ for the drop-casted film), after exposure to DNT vapor, fluorescence quenching of 68% in 2 min and 75% in 4 min was observed, while for TNT, 35% fluorescence quenching in 4.0 min and 69% in 20 min was detected. To extend the practical application, paper strips were fabricated from the polymer more responsive to nitro-explosives, polymer **P28**, by means of fabricating a suspension of nanoparticles, followed by their deposition on paper strips with a polymer surface concentration as low as 1.0 µg cm^−2^. And these strips were effective in detecting various nitro-compounds at a scale less than 1 ng (Figure 74).

**Figure 75:**
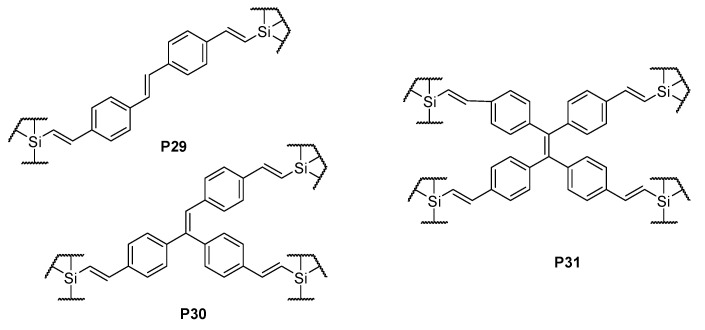
Structures of di-, tri- and tetraphenyletylen-based polymers **P29–31**.

Wang and co-authors reported [133] di-, tri-, and tetraphenyletylen-based polymers **P29**–**31** (Figure 75). According to the obtained results, the porosity of the obtained polymer strongly depends on the substitution degree of ethylene. Thus, compared with polymer **P29** (S_BET_ = 603 m^2^ g^−1^, total pore volume *V*_total_ = 1.01 cm^3^ g^−1^, micropore surface area *S*_micro_ = 60 m^2^ g^−1^, micropore volume *V*_micro_ = 0.02 cm^3^, *V*_micro_/*V*_total_ = 0/02), polymers **P30** and **P31** exhibited enhanced porosities, with *S*_BET_ of 747 (**P30**) and 848 m^2^ g^−1^ (**6**), *V*_total_ of 1.50 (**P30**) and 0.86 cm^3^ g^−1^ (**P29**), and higher microporous properties, with *S*_micro_ of up to 451 m^2^ g^−1^ (**P31**) and *V*_micro_ of 0.21 cm^3^ g^−1^ (**6**). In an ethanol solution, polymer **P30** exhibited dramatic fluorescence quenching, with Stern–Volmer constants (Ksv) of 494 M^−1^ (NT), 1930 M^−1^ (DNT), 21,980 M^−1^ (TNT), 638 M^−1^ (NP), 2100 M^−1^ (DNP), and 24,140 M^−1^ (PA).

**Figure 76:**
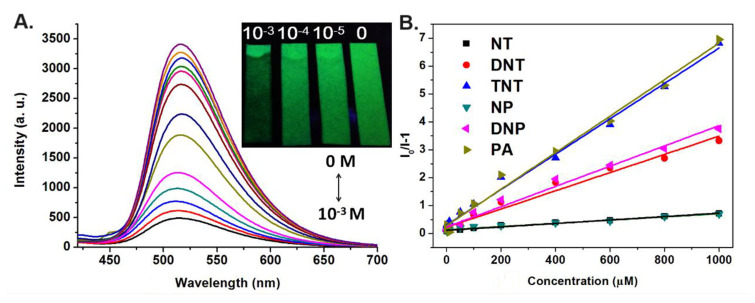
(**A**) Fluorescence-quenching spectra of paper sensors based on polymer **P30** with different concentrations of PA (0~1 × 10^−3^ mol/L) (λex = 365 nm); inset: photos of paper sensors under UV light (365 nm); (**B**) Stern–Volmer plots for paper sensors in the presence of NT, DNT, TNT, NB, DNP, and PA. Reproduced with permission from reference [133]. Copyright © Elsevier 2018.

As the next step, a fluorescent paper sensor was fabricated by means of the simple vacuum filtration of a **P30** dispersion in ethanol, followed by washing and natural drying (Figure 76). The resultant paper sensor was found to be sensitive to explosives in the solution, solid, and vapor phases, with a rapid response time of <10 s, by visually observing the fluorescence-quenching phenomenon. Moreover, this paper sensor was more sensitive to NT (Ksv = 578 M^−1^, LOD 57.3), DNT (Ksv = 3260 M^−1^, LOD = 43.39), and DNP (Ksv = 3630 M^−1^, LOD = 40.84), and it was fully recyclable with a desirable fluorescence recovery ratio, which was higher than 75% after 10 cycles of detection and recycling.

**Figure 77:**
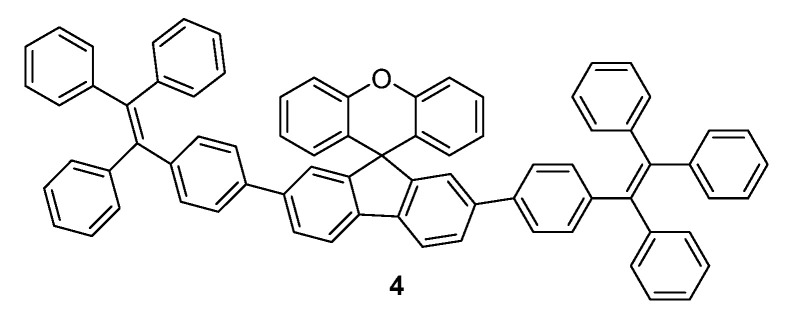
Structure of spirofuorene-9,9′-xantene **4**.

Liang and co-authors reported a paper-supported sensory material based on spirofuorene-9,9′-xantene **4** (Figure 77) that was highly fluorescent in the solid state [134]. This sensory material exhibited a quantitative fluorescence “Turn-off” response to PA in solution with a low detection limit of up to 0.12 nmol/cm^2^ (the response was analyzed by means of the variation in fluorescent images in grayscale). Simplicity and reliability, as well as good thermal stability at temperatures up to 100 °C, are the most attractive features of the reported material.

**Figure 78:**
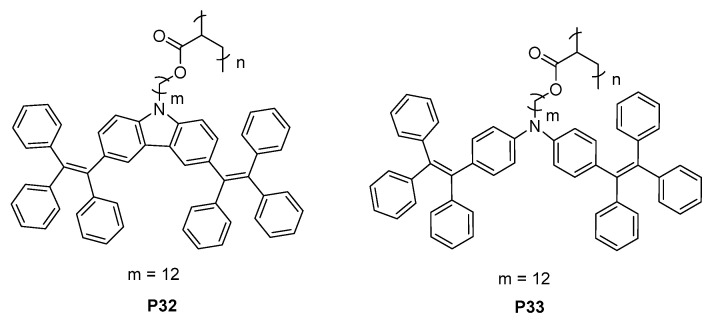
Polymers containing AIE-active fragments of 3,6-bis(1,2,2-triphenylvinyl)carbazole **P32** and bis(4-(1,2,2-triphenylvinyl)phenyl)amine **P33**.

A separate group of sensors for nitroanalytes immobilized on paper carriers comprises polyacrylates containing fluorescent end groups. Thus, Ming Hui Chua and colleagues described a number of polymers containing AIE-active fragments of 3,6-bis(1,2,2-triphenylvinyl)carbazole **P32** and bis(4-(1,2,2-triphenylvinyl)phenyl)amine **P33** linked to the polymer backbone via a flexible alkyl chain (Figure 78) [50]. Paper sensors were also made for the solid-state detection of explosives.

**Figure 79:**
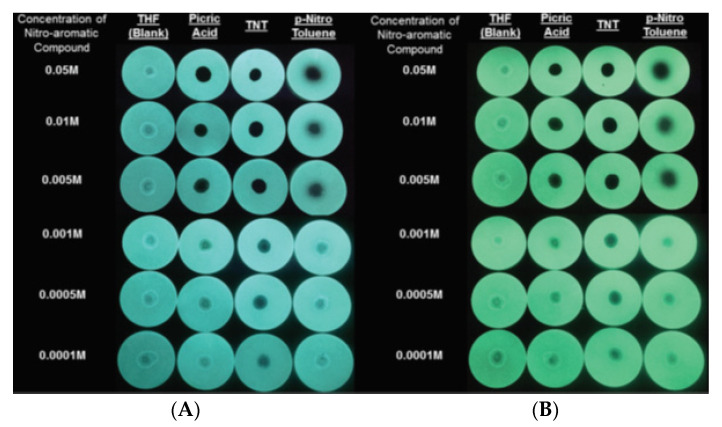
Fluorescence quenching of polymer-coated filter paper (**A**) **P32** and (**B**) **P33** at different concentrations of picric acid, TNT, and *p*-nitrotoluene. Reproduced with permission from reference [50]. Copyright © Wiley 2017.

Polymers **P32** and **P33** exhibited good performance for the detection of TNT with a LOD of 10^−4^ M, which was, however, lower than that for PA and 4-NT (Figure 79).

**Figure 80:**
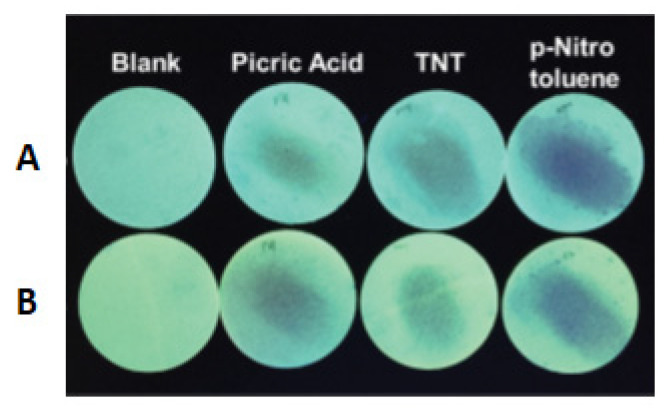
Fluorescence quenching of polymer-coated filter paper (**A**) **P32** and (**B**) **P33** with the dust of nitroaromatic explosives. Reproduced with permission from reference [50]. Copyright © Wiley 2017.

The authors also studied the ability of the above-mentioned paper-based sensors to detect dispersions of nitro-explosives in air (dust) by means of the direct contact of a nitro-explosive-contaminated gloved finger with the surface of polymer-coated filter paper probes. Fluorescence quenching was observed for coated filter paper probes **P32** and **P33** when exposed to a UV lamp (365 nm), as shown in Figure 80. At the same time, mechanical action on the sensor in the absence of nitro-explosive dust does not lead to the quenching of its fluorescence.

**Figure 81:**
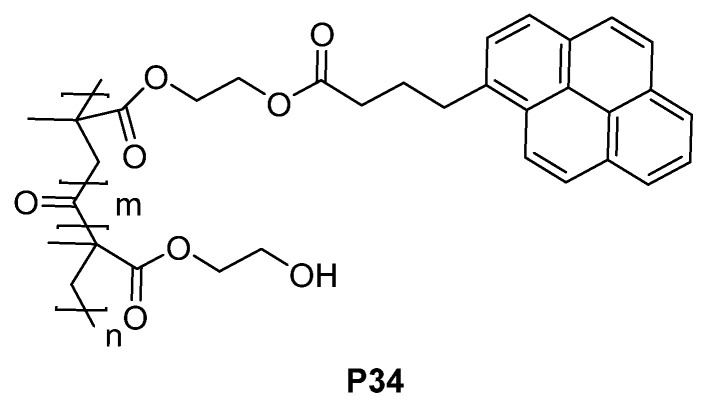
Polyacrylate-based polymer **P34**.

**Figure 82:**
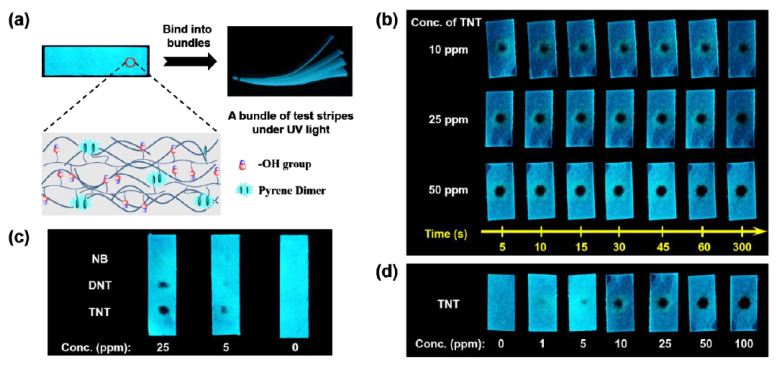
(**a**) Photographs of test strips based on polymer **8** on cellulose paper substrates taken in UV light at a wavelength of 365 nm, as well as their schematic representation; (**b**) fluorescence photographs of paper sensors based on polymer **P34** at various durations (5~300 s) after adding 5 µL of aqueous solutions of various concentrations to these paper sensors at room temperature; (**c**) comparison of fluorescence quenching of sensors based on polymer **P34** under the action of TNT, DNT, and NB; (**d**) fluorescence quenching of sensors based on polymer **P34** caused by aqueous solutions of TNT at various concentrations. Reproduced with permission from reference [135]. Copyright © ACS Publications 2017.

Another polyacrylate-based polymer, **P34**, was reported by Lu and co-authors (Figure 81) [135]. The paper-based sensory material was prepared by means of the direct impregnation of filter paper with a solution of P34. According to the authors, due to the presence of pyrene moieties and oxygen groups, the obtained paper-based material would provide both a good driving force for the transport of NACs inside the material and good interaction contact with pyrene moieties via π-π stacking to result in dramatic fluorescence quenching (Figure 82a).

Upon the treatment of a paper sensor with a solution of NACs, a “turn-off” response was observed after 5 s (in the case of TNT) or longer periods (in the case of DNT and NB) (Figure 82c). These results show that the developed paper sensors provide a much faster fluorescence response than their counterparts on quartz plates. It was also shown that the change in fluorescence was recorded by test strips at TNT concentrations up to 1 ppm (Figure 82d).

**Figure 83:**
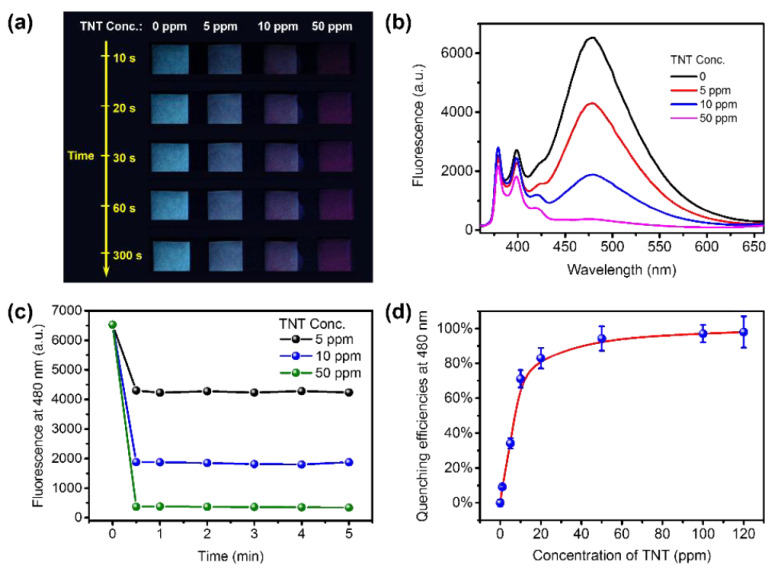
(**a**) Photographs of fluorescence quenching of paper sensors based on polymer **P34** for various durations (10~300 s) after the test paper was immersed in aqueous solutions of TNT at various concentrations at room temperature; (**b**) fluorescent titration spectra of paper sensors based on sensor 8 after treatment with aqueous solutions of TNT at various concentrations; (**c**) profiles of radiation intensity of paper sensors based on polymer **P34** as a function of time in the presence of TNT at various concentrations (5, 10, and 50 ppm); (**d**) extinguishing efficiency values of paper sensors at 480 nm as a function of TNT concentration in the range from 0 to 120 ppm. Reproduced with permission from reference [135]. Copyright © ACS Publications 2017.

In addition to organic solutions, the ability of the test strips to detect NACs in water was also demonstrated (Figure 83). Again, the best results were observed for TNT.

**Figure 84:**
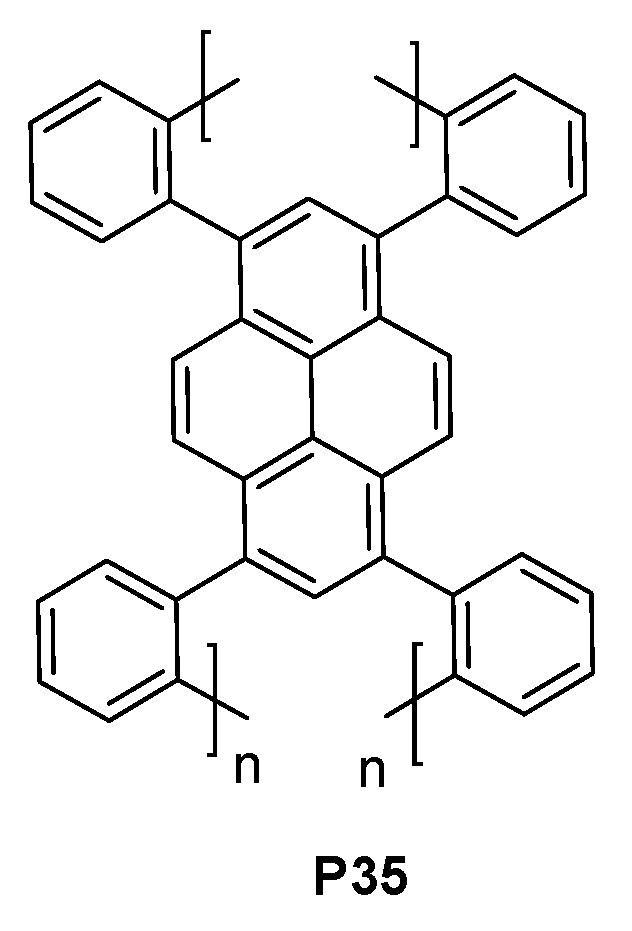
Pyrene-based porous aromatic polymer **P35**.

In 2022, the pyrene-based porous aromatic polymer **P35** was reported (Figure 84) [136].

**Figure 85:**
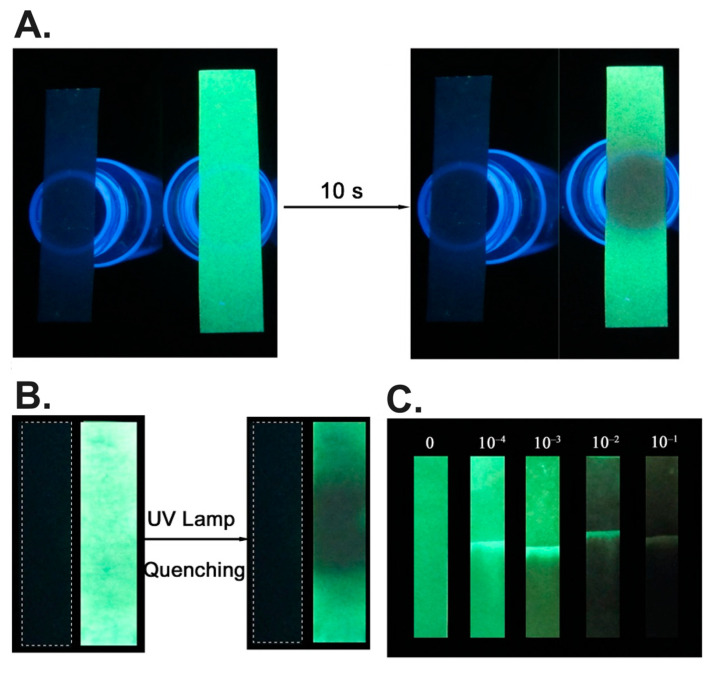
(**A**) Fluorescence photo of indicator paper with polymer **P35** after contact with gaseous nitrobenzene; (**B**) upon detection of nitrobenzene in the form of dust; (**C**) in the presence of various concentrations of liquid nitrobenzene. Reproduced with permission from reference [136]. Copyright © MDPI 2022.

**P35** exhibited strong blue fluorescence with a quantum yield of 18.31%, as well as excellent light and heat resistance. Due to the high-lying LUMO level, polymer **P35** exhibited a strong “turn-off” response to common NACs, with a detection limit (LOD) ≈ 1.47 × 10^−5^ M. When impregnated with **P35**, the paper-based sensor exhibited a visual response to nitrobenzene vapor within 10 s (Figure 85A), as well as nitrobenzene-contaminated dust (Figure 85B) and nitrobenzene solutions (Figure 85C).

**Figure 86:**
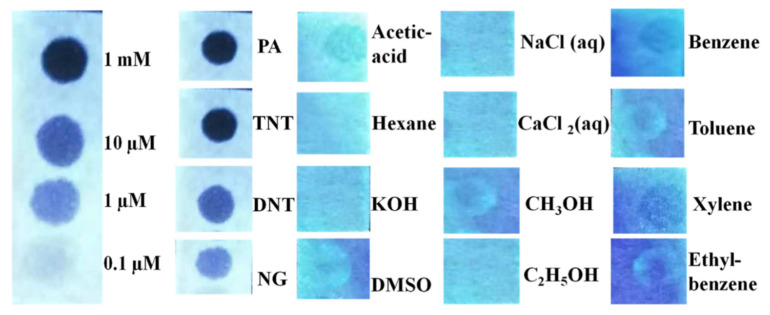
PA detection down to 0.1 µM with fluorescent paper strips (left) and strip selectivity for interfering compounds. Reproduced with permission from reference [137]. Copyright © Elsevier 2020.

Hussain and co-authors reported benzo[ghi]perylene- and coronene-based excimer probes for the selective sensing of NACs [137]. These two compounds are polyaromatic hydrocarbons (PAHs) and tend to form excimers/nanoaggregates, which was observed in an ethanol/water (1:4) mixture. In fluorescence spectra, monomer–excimer dual emissions were detected. As the next step, fluorescent paper strips were prepared by means of impregnating filter paper with solutions of the above-mentioned PAHs. As a result, the thus-obtained materials exhibited strong excimer quenching upon the addition of a drop of a PA solution at various concentrations (Figure 86). According to the authors, the observed detection range for PA varied from 0.1 to 120 µM PA (22.9 ppb–27.5 ppm). In addition, the authors tested for the presence of a response to non-explosive compounds, such as volatile organic solvents, including aromatics (toluene, xylene, and ethylbenzene), as well as acids and bases. As a result, it was shown that the fluorescence of the strips remained unchanged, which demonstrates the high selectivity of this test system for foreign substances.

**Figure 87:**
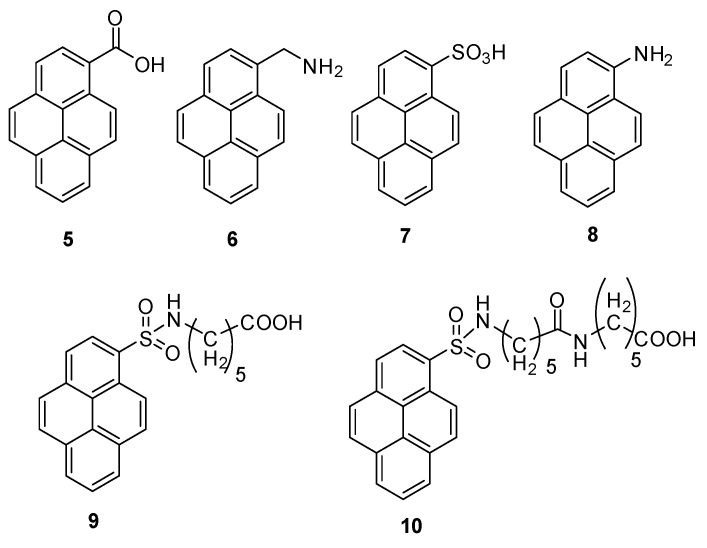
Pyrene derivatives **5–8** and micelle-like aggregates/preaggregates **9–10**.

We reported a series of pyrene derivatives **5**–**8**, as well as micelle-like aggregates/preaggregates **9**–**10** obtained from **7** by means of the introduction of surface-active hydrophilic “heads” onto the periphery of lipophilic “tails” (Figure 87) [138]. As a result, in aqueous solutions at a concentration of 10^−5^ M, the formation of highly fluorescent micelle-like aggregates/preaggregates was observed.

**Figure 88:**
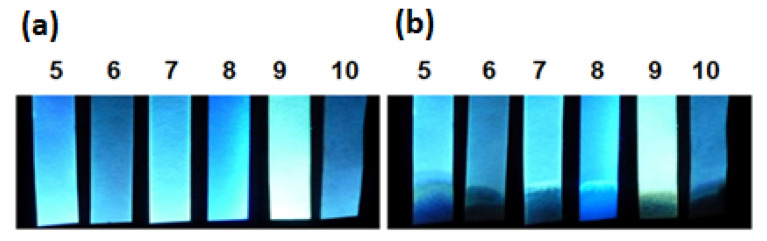
(**a**) Paper strips impregnated with aqueous solutions of sensors **5–10** in UV light; (**b**) the same paper strips after brief contact (10 s) with a solution of TNT in water (50 ppb) under UV light. Reproduced with permission from reference [138]. Copyright © Wiley 2016.

For all of the chemosensors, in the presence of NACs, improved fluorescence quenching was achieved with Stern–Volmer quenching constants close to 10^5^ M^−1^ and a detection limit of only 182 ppb, and the most hydrophilic probes, **9**–**10**, showed a higher response to 2,4-DNT than to TNT. As the next step, paper strips impregnated with 10^−5^ M solutions of **5**–**10** were fabricated. These paper strips were capable of the “turn-off” fluorescence detection of TNT, 2,4-DNT, and other nitro-explosives at a level of 50 ppb in water for 10 s (Figure 88).

**Figure 89:**
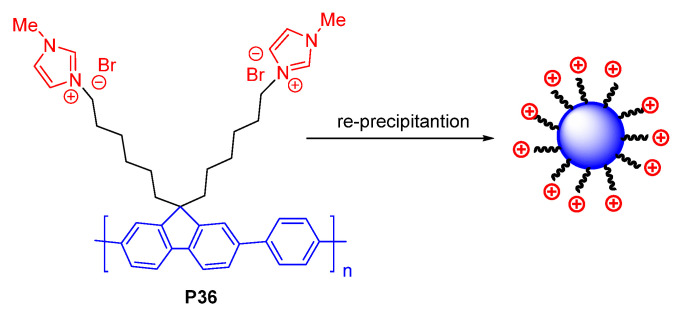
Structure of newly synthesized polymer **P36** and its corresponding nanoparticles.

In addition to PAHs, a significant number of examples of paper sensors for nitroanalytes based on fluorophores bearing residues of nitrogen-containing heterocycles are described in the literature. Thus, a group of researchers from India reported a polymer cationic sensor, poly(3,3′-((2-phenyl-9H-fluorene-9,9-diyl)bis(hexane-6, 1-diyl))bis(1-methyl-1*H*-imidazol-3-y)bromide), **P36**, for the subsequent preparation of nanoparticles (Figure 89) [38].

**Figure 90:**
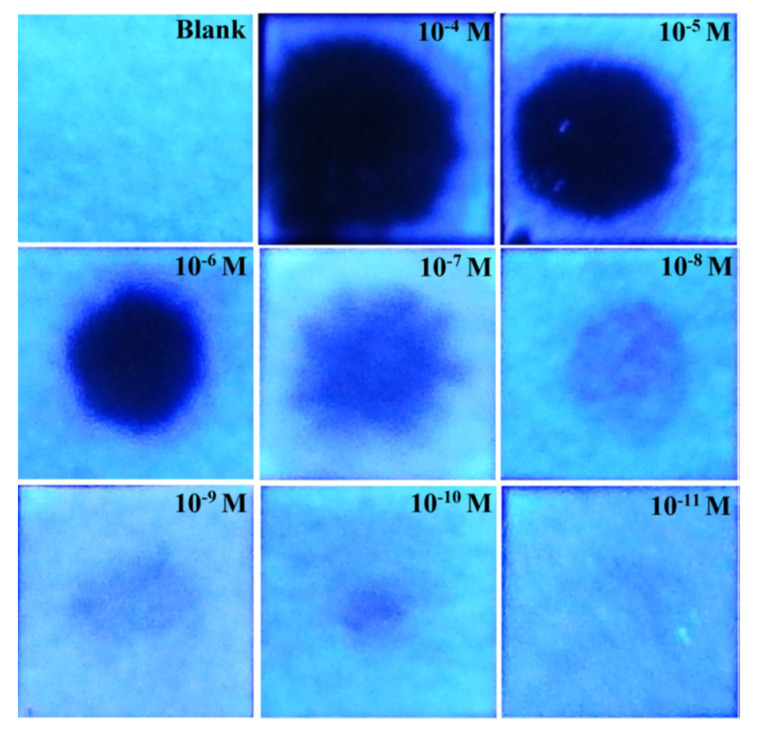
Photos of fluorescent test strips based on polyelectrolyte **P36** in UV light after adding 10 µL of various concentrations of PA. Reproduced with permission from reference [38]. Copyright © ACS Publications 2015.

The authors described experiments on the detection of PC vapor with the contact method using fluorescent paper strips based on the obtained polymer, **P36** (Figure 90). The ability of the sample to detect PC in vapor with a concentration up to 10^−11^ M and a detection limit of 22.9 fg/cm^2^ was shown, which makes it one of the best indicators among the sensors described in the literature. The authors attributed the observed oversensitivity mechanism of the PA detection probe to the “molecular wire effect” and electrostatic interactions, combined with PET and possibly RET mechanisms.

**Figure 91:**
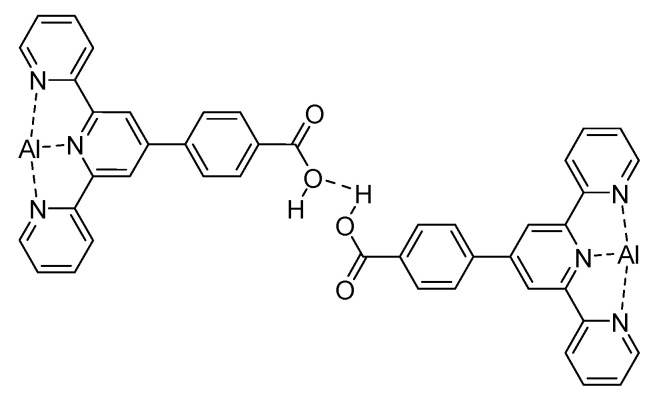
Complex of trivalent aluminum with 4-[2,2′:6′,2″-terpyridine]-4′-ylbenzoic acid.

Mao Xia Guo et al. reported a representative of the class of luminescent organometallic gels based on a complex of trivalent aluminum with 4-[2,2′:6′,2″-terpyridine]-4′-ylbenzoic acid (Figure 91) [139]. 

**Figure 92:**
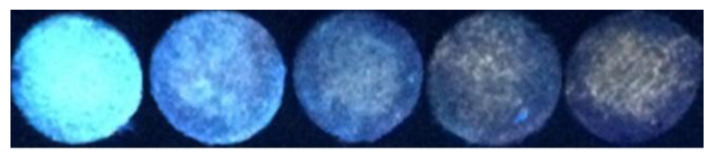
Paper-based sensory material for recognition of PA in solutions with varying concentrations (from left to right): control sample; 1.0 × 10^−5^; 5.0 × 10^−5^; 1.0 × 10^−4^; 2.0 × 10^−4^ M (λex = 365 nm). Reproduced with permission from [139]. Copyright © Elsevier 2017.

The authors reported the ability of the complex to selectively recognize hydroxyl nitroaromatic compounds, including those in mixtures with other analytes that do not contain OH groups. The quenching of the fluorescence of test paper sensors based on the complex at 467 nm by PA solutions with different concentrations is described. As shown in Figure 92, the fluorescence intensity of the test paper gradually decreased with an increase in the concentration of teardrop-shaped PA from 10^−5^ M.

**Figure 93:**
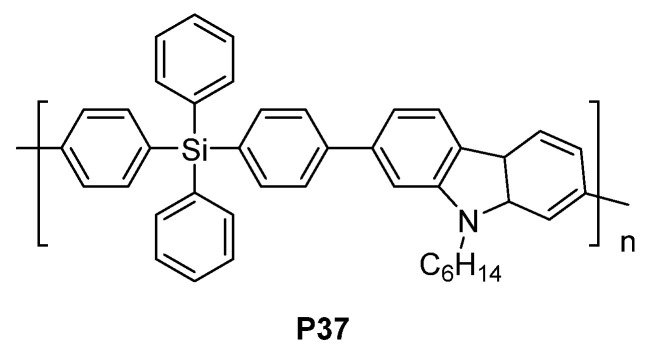
Copolymer **P37** based on *N*-hexylcarbazole and tetraphenylsilane.

Guo and colleagues described the copolymer **P37** based on *N*-hexylcarbazole and tetraphenylsilane for the detection of nitroaromatic compounds (Figure 93) [140]. In addition to fluorescent and sensory properties, among the advantages of the polymer, the authors emphasize the high glass transition temperature and good thermal stability of the resulting copolymer.

**Figure 94:**
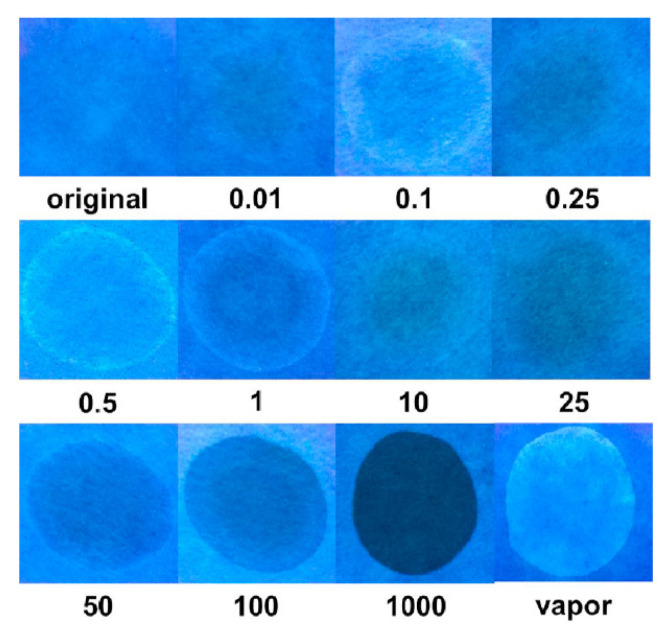
Paper strips based on copolymer before and after contact with THF solution (concentrations: 0.01, 0.1, 0.25, 0.5, 1, 10, 25, 50, 100, and 1000 µg/mL) and after storage over THF vapor for 25 min at 365 nm excitation. Reproduced with permission from reference [140]. Copyright © Elsevier 2021.

For potential in-field applications, **P37**-copolymer-based paper strips were fabricated for experiments on the detection of PA in solutions and vapors. According to Figure 94, with an increase in the concentration of PA in solution up to 1000 μg/mL, the progressive quenching of the fluorescence of the sample is observed. However, it is noted that apparent quenching is unambiguously recognizable at a concentration of 0.01 μg/mL PA in solution. In addition, the quenching of the image emission under the influence of TNP vapor for 25 min is shown.

**Figure 95:**
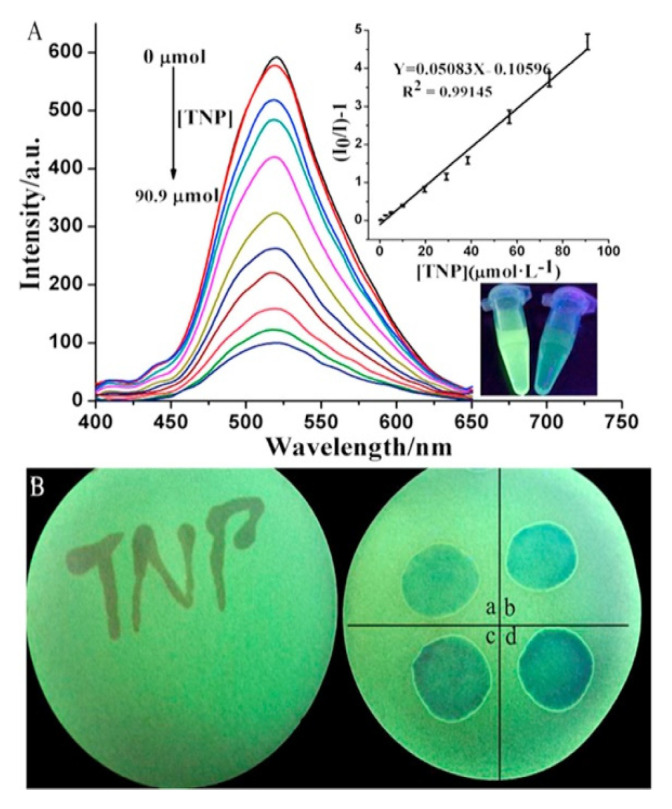
(**A**) Fluorescence quenching of the suspension of **Bhq-Al** in the presence of TNP; (**B**) fluorescence quenching of paper-based material upon deposition of TNP at various concentrations. Reproduced with permission from reference [141]. Copyright © Elsevier 2016.

In 2016, a fluorescent complex of Al^3+^ and bis(8-hydroxyquinoline) (**Bhq-Al**) was prepared and applied for the detection of PA (TNP) [141]. In a formamide solution, this complex formed a suspension with an emission peak at 519 nm upon excitation at 388 nm. In the presence of TNP (90.9 μM), the fluorescence intensity of **Bhq-Al** was reduced by 83.1%. As the next step, the paper-based sensor was prepared by means of the impregnation of filter paper with a solution of **Bhq-Al**, and it was shown that the quenching of the sensor emission was noticeable at a concentration of 2 μM TNP (Figure 95).

**Figure 96:**
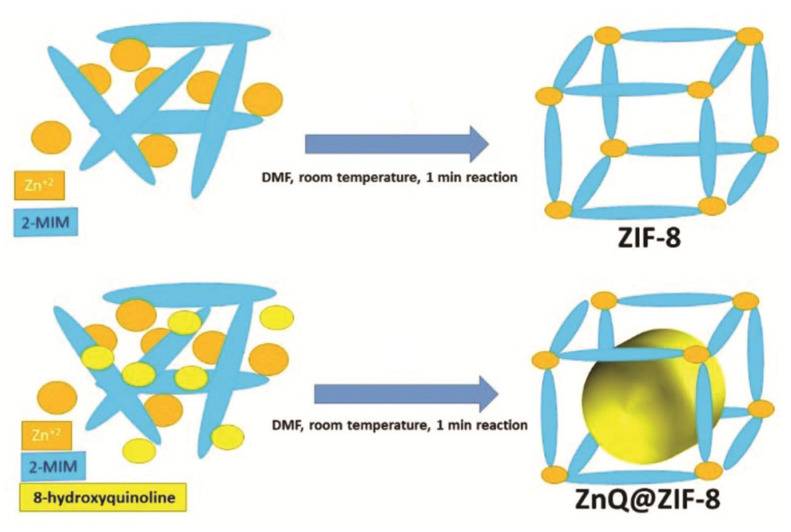
Schematic diagram illustrating the strategy of the in situ encapsulation of **ZnQ** guest in **ZIF-8** voids. Reproduced with permission from reference [142]. Copyright © Royal Society of Chemistry 2020.

A Canadian–Egyptian research team [142] developed a fluorescent organometallic framework based on zeolitic imidazole (**ZIF-8**) doped with fluorescent 8-hydroxyquinoline zinc (**ZnQ**) (Figure 96). In comparison with the fluorescence of **ZnQ** at ∼420 nm, the resulting **ZnQ@ZIF-8** exhibited red-shifted emission at ∼520 nm due to the encapsulation of **ZnQ** guests inside the **ZIF-8** matrix. As the next step, a paper-based sensor was prepared.

**Figure 97:**
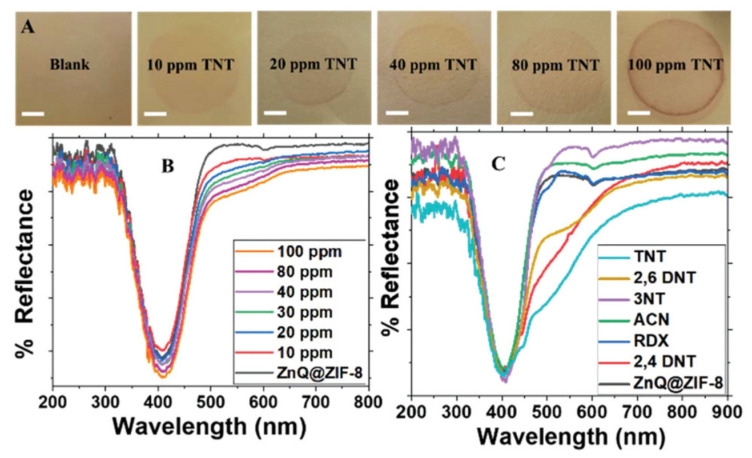
(**A**) Images of a paper sensor coated with **ZnQ@ZIF-8** in contact with solutions of TNT at various concentrations; (**B**) UV–visible reflectance spectra as a function of TNT concentration; (**C**) UV–visible reflectance spectra in contact with various nitroaromatic compounds. Reproduced with permission from reference [142]. Copyright © Royal Society of Chemistry 2020.

When applying various concentrations of TNT, the authors observed a visible color change from ivory to light red in about one minute, indicating an interaction between TNT and **ZnQ@ZIF-8**, as shown in Figure 97A.

**Figure 98:**
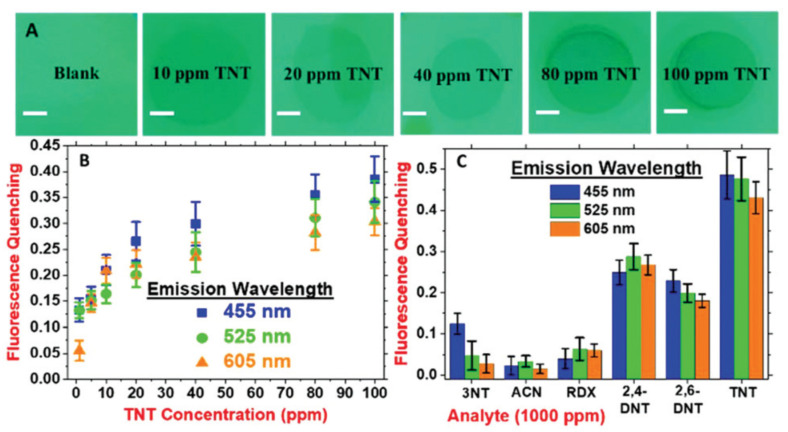
(**A**) Images of paper sensor coated with **ZnQ@ZIF-8** as a function of TNT concentration under UV irradiation (λ_ex_ = 365 nm); (**B**) fluorescence quenching at various wavelengths (λ_em_ = 455 nm, 525 nm, and 605 nm) for a sensor coated with **ZnQ@ZIF-8** depending on the TNT concentration from 1 to 100 ppm; (**C**) fluorescence quenching at different wavelengths (λ_em_ = 455 nm, 525 nm, and 605 nm) for a **ZnQ@ZIF-8**-coated sensor upon contact with various nitroaromatic compounds. Reproduced with permission from reference [142]. Copyright © Royal Society of Chemistry 2020.

This interaction proceeds between the electron-deficient aromatic nucleus of TNT and the electron-rich **ZnQ@ZIF-8** fragments, which contribute to the formation of a donor–acceptor electron transfer mechanism, which, consequently, causes a color change in the visible range. It is noted that the lowest concentration of TNT that causes a visible color change observed by the naked eye is 3 ppm. As shown in Figure 98B, as the concentration of TNT increases, the absorption of light with a wavelength of about 500 nm increases, resulting in a color change in the visible region. However, UV–visible reflectance measurements also showed no interaction after the addition of TNT, RDX, and ACN at low concentrations (1000 ppm), as shown in Figure 98C.

When exposed to TNT on a paper sensor coated with **ZnQ@ZIF-8**, fluorescence quenching is also observed upon irradiation with portable UV light (λ_ex_ = 365 nm) (Figure 98A). A decrease in bluish-green fluorescence was noted as the concentration of TNT increased from 10 to 100 ppm. The fluorescence intensities at different radiation wavelengths (λ_em_ = 455 nm, 525 nm, and 605 nm) obtained using a fluorescent microscope are shown in Figure 96b. It was noted that the maximum quenching of fluorescence always occurs in the blue emission mode at 455 nm.

The authors also found that the bluish-green fluorescence intensity was not significantly affected by the addition of other nitroaromatic compounds, including TNT, RDX, and ACN, as shown in Figure 98C. However, some changes in fluorescence quenching were noted with the addition of 2,4-DNT and 2,6-DNT, which means that high concentrations of TNT by-products are also detected with this paper sensor. This fact indirectly confirms the emission-quenching mechanism of the described nanomaterial.

**Figure 99:**
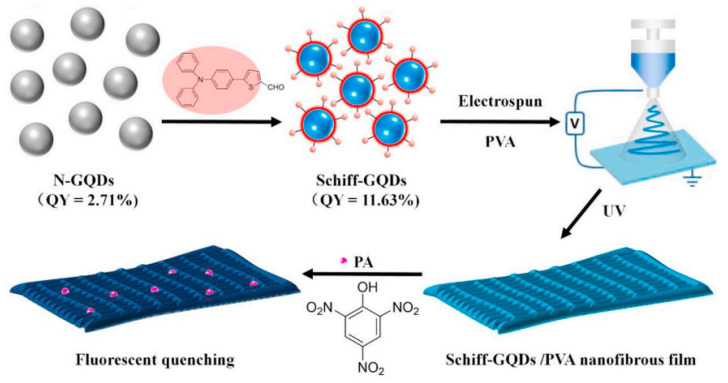
Schematic illustration of the sensitive detection of PA based on **Schiff-GQDs**. Reproduced with permission from reference [143]. Copyright © Elsevier 2021.

In 2021, a Schiff-base-functionalized graphene quantum dot nanocomposite PA sensor (**Schiff-GQD**) was reported (Figure 99) [143]. According to the authors, the fluorescence quantum yield of **Schiff-GQD** increased to as high as 11.63% (due to the AIE effect), which was higher than that of graphene oxide quantum dots (N-GQDs) (2.71%) and seven times higher than that of triphenylamino-substituted thiophene-2-carbaldehyde (1.51%). As the next step, an electrospun material, **Schiff-GQD**/PVA, was prepared, and the obtained material exhibited up to 82.5% at a PA concentration of 2.5 × 10^−4^ M.

**Figure 100:**
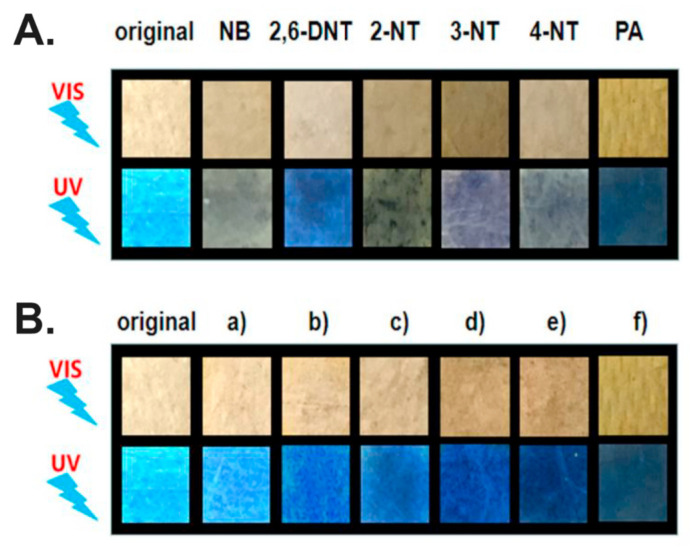
(**a A**) Visual matrix image of paper-based materials before and after the addition of explosives; (**B**) test strips based on sensor for visual determination of various concentrations of PA: initial 0 M, (**a**) 1.0 × 10^−6^ M, (**b**) 5.0 × 10^−6^ M, (**c**) 1.0 × 10^−5^ M, (**d**) 1 × 10^−4^ M, (**e**) 2 × 10^−4^ M, and (**f**) 4.4 × 10^−4^ M in visible and 365 nm UV illumination. Reproduced with permission from reference [143]. Copyright © Elsevier 2021.

More importantly, paper-based **Schiff-GQD** sensor materials were fabricated using a self-assembly method for the visual detection of PA by immobilization on filter paper (Figure 100a).

After contact with various PACs, the test paper showed various color changes, and in the presence of PA, intensive fluorescence and color changes were observed (Figure 100B). As the PA concentration gradually increases, the concentration of the fluorescent indicator paper gradually decreases. The color changes significantly under the influence of UV radiation, and the limit of detection, visible to the naked eye, reaches 11.45 ng/cm^2^.

**Figure 101:**
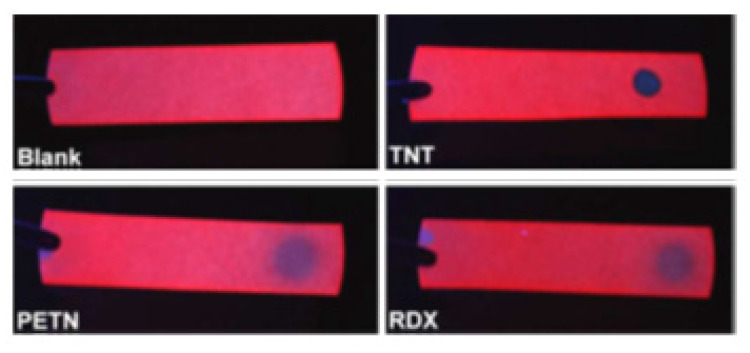
Photographs of luminescent silicon-nanomaterial-coated filter paper under a handheld UV lamp in the presence of solutions of various nitro-explosives. Reproduced with permission from reference [144]. Copyright © Royal Society of Chemistry 2014.

In rare cases, paper-based matrices can be doped with inorganic luminophores. Thus, in research by a Canadian–German team [144], a paper sensor based on luminescent silicon nanocrystals was reported. For improved selectivity, the surface was functionalized with dodecyl groups, which are sensitive to solutions of nitroanalytes, including RDX and PETN. The results of the experiments (Figure 101) described by the authors show that the sample is more effective at a concentration of 0.01 mM nitroanalytes, while no visible differences in the response were observed for any concentrations of RDX and PETN. In the case of DNT, however, a sharp increase in quenching is observed in the region surrounding the initial compound spot.

## 4. Conclusions and Future Perspectives

In the face of growing terroristic threats, one can see an intense interest in improving explosive detection. Of the many detection methods, fluorescence-based methods and materials are among the most promising tools for the detection of (nitro-)explosives due to their high sensitivity and selectivity, fast response time, simplicity, portability, and, in most cases, low cost. Therefore, fluorescence-based sensory materials can be easily and effectively integrated into sensing devices for remote (nitro-)explosive detection, for instance, the well-known Fido explosive detector [145], as well as other types of “electronic noses” [146,147]. In addition, some simpler devices and approaches can be introduced, for instance, mobile-phone-based explosive detection systems [148] or computer-vision-based algorithms [149].

Indeed, fluorescence-based conjugated polymers provide a fast and intensive response and good selectivity and sensitivity to the most common nitro-explosives, such as TNT, DNT, and PA, while in the case of aliphatic nitro-explosives, such as PETN, RDX, and others, these materials exhibit a low or no sensory response. The synthetic preparation of such polymers always requires multi-step synthesis, starting from specially designed monomers, and these issues make the mass production of sensory materials based on the above-mentioned polymers complicated and expensive. Another serious issue is the low durability and low thermo- and photostability of polymers. Finally, in the case of the detection of explosives in the vapor phase, the effectiveness of the sensory response depends strongly on the thickness of the polymer film and/or on the effective contact surface to provide efficient mass exchange. To overcome this issue, porous polymer materials can be introduced; for instance, iptycene-based or molecularly imprinted polymer-based aggregates or nanoparticles can be constructed, or electrospun materials can be used [150,151].

In terms of the mechanical properties of polymers, the impregnation of commercially available polymers with single-molecule sensors [152,153] or sensor arrays [154] or bio-/naturally occurring polymer-based materials can be used [155] as a way to increase the selectivity and sensory response of the polymer materials.

In the case of semiconductive organic polymers, bi-modular sensors for explosive detection can be designed, and these materials can detect explosives in dual modes. In this case, in addition to fluorescence “turn-off” detection, these polymers also show changes in electrochemical properties in the presence of explosives [156]. This results in improved sensing reliability, sensitivity, or selectivity due to the integration of two independent sensing technologies into one sensory material.

Fluorescence “turn-off” (fluorescence quenching) via PET, FRET, the “molecular wire” effect, or even the inner-filter effect is a common and the most understood method for fluorescence-based explosive detection. In this regard, PAH-containing fluorophores may be introduced into the sensory material to provide an excimer/excimer-like-based photoluminescence response for explosive detection [157,158]. In addition, the introduction of AIE-genic groups can be used to develop materials for the selective and sensitive detection of some explosives [24]. Finally, there is great promise in developing sensing systems for the detection of nitro-explosives based on a “turn-on” detection mechanism, where nitro-analytes will interrupt/destroy the quenching process, which results in dramatic fluorescence enhancement [159].

Finally, more detailed data are needed for a better interpretation/prediction of explosive-sensing mechanisms and the correlation of the connection between the nature of the sensing material, including its physical and mechanical properties, and the nature of its sensory response and sensing performance. So far, most explosive-sensing materials are made empirically and/or by a method of trial and error. The main reasons for this are the absence of both systematic literature analysis and theoretical methods for the better prediction of electronic and sensing performance for the proper material design.

## Abbreviations

ACQ	Aggregation-caused quenching
AFP	Amplifying fluorescent polymers
AIE	Aggregation-induced emission
CD	Carbon dots
CP	Conjugated polymer
DNP	Dinitrophenol
DNT	Dinitrotoluene
FRET	Förster resonance energy transfer
K_SV_	Stern–Volmer constant
LOD	Limits of detection
MIP	Molecularly imprinted polymer
NACs	Nitroaromatic compounds (nitroaromatics)
NB	Nitrobenzene
NT	Nitrotoluene
PA (or TNP)	Picric acid (or trinitrophenol)
PAH	Polycyclic aromatic hydrocarbon
PET	Photoinduced electron transfer
PETN	Pentaerythritol tetranitrate
RDX	Research Department eXplosive (hexagen)
RET	Resonance energy transfer
S_BET_	Surface of a porous solid material calculated according to Brunauer–Emmett–Teller theory
TNT	Trinitrotoluene
TPE	Tetraphenylethene
